# A comprehensive comparison of deep learning-based compound-target interaction prediction models to unveil guiding design principles

**DOI:** 10.1186/s13321-024-00913-1

**Published:** 2024-10-28

**Authors:** Sina Abdollahi, Darius P. Schaub, Madalena Barroso, Nora C. Laubach, Wiebke Hutwelker, Ulf Panzer, S.øren W. Gersting, Stefan Bonn

**Affiliations:** 1 Institute of Medical Systems Biology, University Medical Center Hamburg-Eppendorf, Hamburg, 20251 Germany; 2 III. Department of Medicine, University Medical Center Hamburg-Eppendorf, Hamburg, 20251 Germany; 3 University Children’s Research, UCR@Kinder-UKE, University Medical Center Hamburg-Eppendorf, Hamburg, 20251 Germany; 4 Hamburg Center for Translational Immunology (HCTI), University Medical Center Hamburg-Eppendorf, Hamburg, 20251 Germany; 5 Center for Biomedical AI, University Medical Center Hamburg-Eppendorf, Hamburg, 20251 Germany

**Keywords:** Drug-target interaction prediction, Deep learning, Gold-standard datasets, Mutated targets, Drug embeddings, Protein descriptors, Protein trainable embeddings

## Abstract

The evaluation of compound-target interactions (CTIs) is at the heart of drug discovery efforts. Given the substantial time and monetary costs of classical experimental screening, significant efforts have been dedicated to develop deep learning-based models that can accurately predict CTIs. A comprehensive comparison of these models on a large, curated CTI dataset is, however, still lacking. Here, we perform an in-depth comparison of 12 state-of-the-art deep learning architectures that use different protein and compound representations. The models were selected for their reported performance and architectures. To reliably compare model performance, we curated over 300 thousand binding and non-binding CTIs and established several gold-standard datasets of varying size and information. Based on our findings, DeepConv-DTI consistently outperforms other models in CTI prediction performance across the majority of datasets. It achieves an MCC of 0.6 or higher for most of the datasets and is one of the fastest models in training and inference. These results indicate that utilizing convolutional-based windows as in DeepConv-DTI to traverse trainable embeddings is a highly effective approach for capturing informative protein features. We also observed that physicochemical embeddings of targets increased model performance. We therefore modified DeepConv-DTI to include normalized physicochemical properties, which resulted in the overall best performing model Phys-DeepConv-DTI. This work highlights how the systematic evaluation of input features of compounds and targets, as well as their corresponding neural network architectures, can serve as a roadmap for the future development of improved CTI models.

**Scientific contribution**

This work features comprehensive CTI datasets to allow for the objective comparison and benchmarking of CTI prediction algorithms. Based on this dataset, we gained insights into which embeddings of compounds and targets and which deep learning-based algorithms perform best, providing a blueprint for the future development of CTI algorithms. Using the insights gained from this screen, we provide a novel CTI algorithm with state-of-the-art performance.

**Supplementary Information:**

The online version contains supplementary material available at 10.1186/s13321-024-00913-1.

## Introduction

A compound typically manifests its therapeutic effects by interacting with proteins, referred to as compound targets. An interaction between a compound and its target can regulate the target’s activity, either by enhancing or suppressing its function. A comprehensive understanding of the interaction between a compound and its target is imperative for effective drug development. Researchers investigate the structure and functionality of compounds and their respective targets to identify potential binding sites and facilitate the repurposing and development of compounds capable of interacting with them [1]. Additionally, predicting potential off-target interactions is vital for assessing the potential risks linked to a compound and formulating approaches to mitigate undesired side effects [2]. Due to the high cost associated with wet-lab experimental methods and the enormous number of potential compound-target interactions (CTIs), deep learning-based models have been developed to address the scale and complexity of the drug discovery process [3].

CTI prediction encounters several significant challenges, with some crucial ones outlined below: (i) Data sparsity: CTI prediction models depend extensively on labeled data for training. However, obtaining experimental data can be limited, particularly for less-explored targets or novel compounds. This data sparsity issue arises due to varying data reliability and quality, potentially introducing biases and errors in predictions. (ii) Promiscuity of compounds and proteins: Many proteins participate in multiple signaling pathways, and compounds often interact with multiple targets, leading to complex interactions that can be difficult to predict accurately. (iii) Availability of structural information: The accuracy of CTI prediction models is impacted by the availability of high-quality structural data on both compounds and targets. Lack of detailed structural information can hinder the understanding of binding mechanisms and interactions between compounds and their targets, affecting prediction reliability. (iv) Drug toxicity and adverse side effects: A thorough understanding of compound selectivity is vital for successful drug development and safe clinical application. To address these challenges, it is crucial to utilize informative features encompassing comprehensive information and encoding structural and sequential characteristics of compounds and targets. Incorporating such features into prediction models can enhance accuracy and reliability in CTI predictions.

CTI prediction models are typically stratified into four categories based on their input features: sequence-based, structure-based, sequence-structure-based (hybrid), and knowledge graph (KG)-based models [4]. Sequence-based models process compounds and targets as character sequences and are sometimes implemented using language-based models such as transformers [5, 6]. For each character, the representative vector can be chosen from various options, such as one-hot vectors, trainable embeddings, physicochemical properties, k-mers embeddings, and learned embeddings derived from a trained model [7, 8]. In some cases, subsequences of compounds and targets are used as input features. For example, MolTrans employs a sequential pattern approach to extract a hierarchical set of frequent subsequences from compounds and targets [9]. The structure-based models aim to extract features from the two- (or three-) dimensional structure of proteins or compounds. Based on the structural information, different models attempt to represent them in two main formats: graph-based representations, such as atom and residue interaction networks, or vector-based representations, such as drug fingerprints and protein descriptors. These models commonly employ graph-based neural networks, such as graph attention (GAT) layers or graph convolutional networks (GCNs), to facilitate this process [10, 11]. Hybrid models have been devised by fusing the sequential and structural information of proteins and compounds [12]. In some approaches, the sequence of a protein (or compound) and the 2D structure of a compound (or protein) are used as input features [13]. For example, Chen et al. introduced an encoder-decoder model that incorporates BERT-based embeddings for targets and the atom interaction network of compounds as input features [14]. The KG-based models have emerged by integrating diverse data sources into a unified framework for CTI prediction. These sources include protein-protein interactions, compound-compound interactions, and similarity networks [15, 16].

**Fig. 1:**
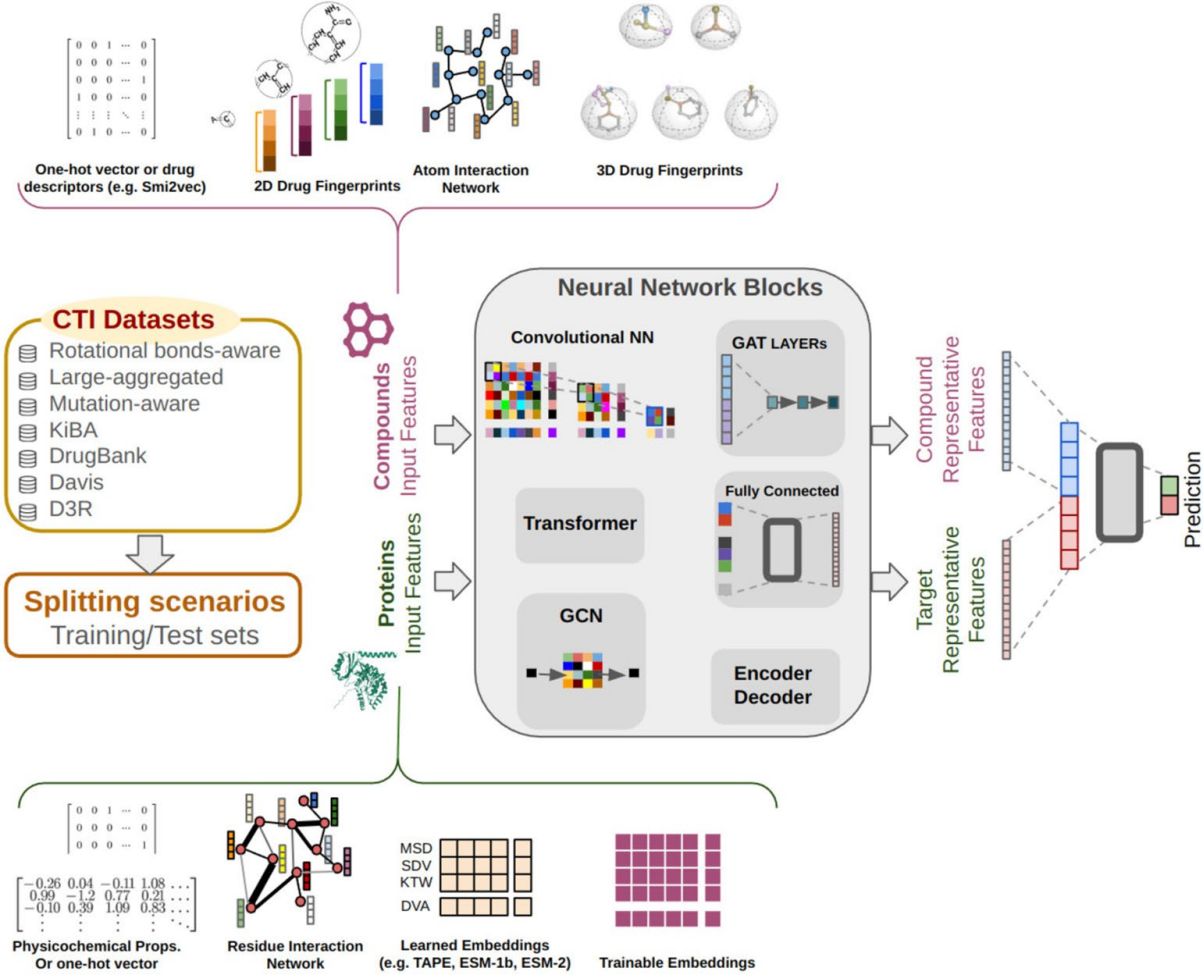
The overall workflow illustrating the three phases: creation of gold-standard datasets, extraction of various protein and compound input features, and the utilization of different neural network blocks to predict compound-target interactions

Recently, Guvenilir et al. [17] conducted a study on target featurization using two machine learning-based models, support vector machine (SVM) and random forest (RF). However, their comparison of target input features does not include 2D and 3D structure-based features of targets and compounds or deep learning-based representations thereof. Investigating the impact of various input feature types and model architectures on the performance of CTI prediction enables the identification of more effective representative embeddings and appropriate neural network blocks for proteins and compounds. Informative and representative features are expected to be capable of encoding valuable information about molecular structure, functional groups, and other relevant descriptors that influence CTI. Hence, it is anticipated that the distinctive feature vectors of two proteins or compounds that are similar to each other (with minor structural differences) will be clearly distinguished, and these dissimilarities will be encoded within the feature vectors themselves.

**Table 1:** The overview of the different models

Model	Input features of compounds	Input features of targets	Neural network blocks
2DFP-based	Morgan, MACCS, RDKit-2D, and AtomPair 2D Fingerprints	Physicochemical Properties	CNN$^{1}$ and FC$^{2}$
AlphaFoldGrAtts	Atoms’ interaction network + Chemical/Structural Properties of atoms	Residue interaction network + Physicochemical Properties	GATv2$^{3}$ and FC
BERT-based	Morgan, MACCS, RDKit-2D, and AtomPair Fingerprints	Learned Embeddings: BERT (768-d)	FC
DeepCAT [18]	One-hot vectors of SMILES	Physicochemical Properties	CNN and FC
DeepConv-DTI [8]	Morgan 2D Fingerprint	Trainable Embeddings	Multiple CNN and FC
DeepDTA [7]	Trainable Embeddings	Trainable Embeddings	CNN and FC
E3FP-based	E3FP 3D Fingerprint	Physicochemical Properties	CNN and FC
IIFDTI [5]	Atoms’ interaction network + Smi2Vec	Prot2Vec + Trainable Embeddings	Bidirectional encoder-decoder, GAT, and FC
PhyChemDG	Atoms’ interaction network + Chemical/Structural Properties of atoms	Physicochemical Properties	Transformer, GCN$^{4}$ ,and FC
PhyGrAtt	Atoms’ interaction network + Chemical/Structural Properties of atoms	Physicochemical Properties	GATv2, CNN, and FC
RF	One-hot vectors of SMILES	Physicochemical Properties	Random Forest
TransformerCPI [6]	Atoms’ interaction network + Chemical/Structural Properties of atoms	K-mers + Word2Vec	Transformer, GCN ,and FC
UniRep-based	Morgan, MACCS, RDKit-2D, and AtomPair Fingerprints	Learned Embeddings: UniRep (1900-d)	FC

$^{1}$ Convolutional Neural Network blocks

$^{2}$ Fully Connected Layers

$^{3}$ Graph Attention v2 Layers

$^{4}$ Graph Convolutional Network blocks

In pursuit of this goal, we assessed the performance of state-of-the-art sequence-based, structure-based, and hybrid models to investigate the capabilities and contributions of diverse input features and their respective neural network architectures. Given the nature of the KG-based models, they do not provide a precise delineation of the individual contribution of each input feature to the prediction performance. Consequently, we excluded them from further consideration.

Evaluating various input features and models necessitates the use of reliable and comprehensive datasets. In this study, we employed distinct datasets that varied from uniformly distributed proteins or compounds across the training and test sets (warm-start splitting scenario) to test sets that encompass unseen proteins or compounds (cold-start splitting scenario). Additionally, it is crucial to compare the performance of the models over both a large aggregated dataset and separate datasets of varying sizes. To further assess the models’ capability to capture informative features of targets, we curated a dataset comprising training data containing records of wild-type targets and test data containing records of mutant targets. Besides the mentioned datasets, we have also compiled an additional dataset that takes into account the presence of rotatable bonds in compounds. In essence, our interest lies in assessing how well the models perform when the dataset includes or excludes compounds with a significant number of rotatable bonds, particularly in terms of predicting interactions.

**Fig. 2:**
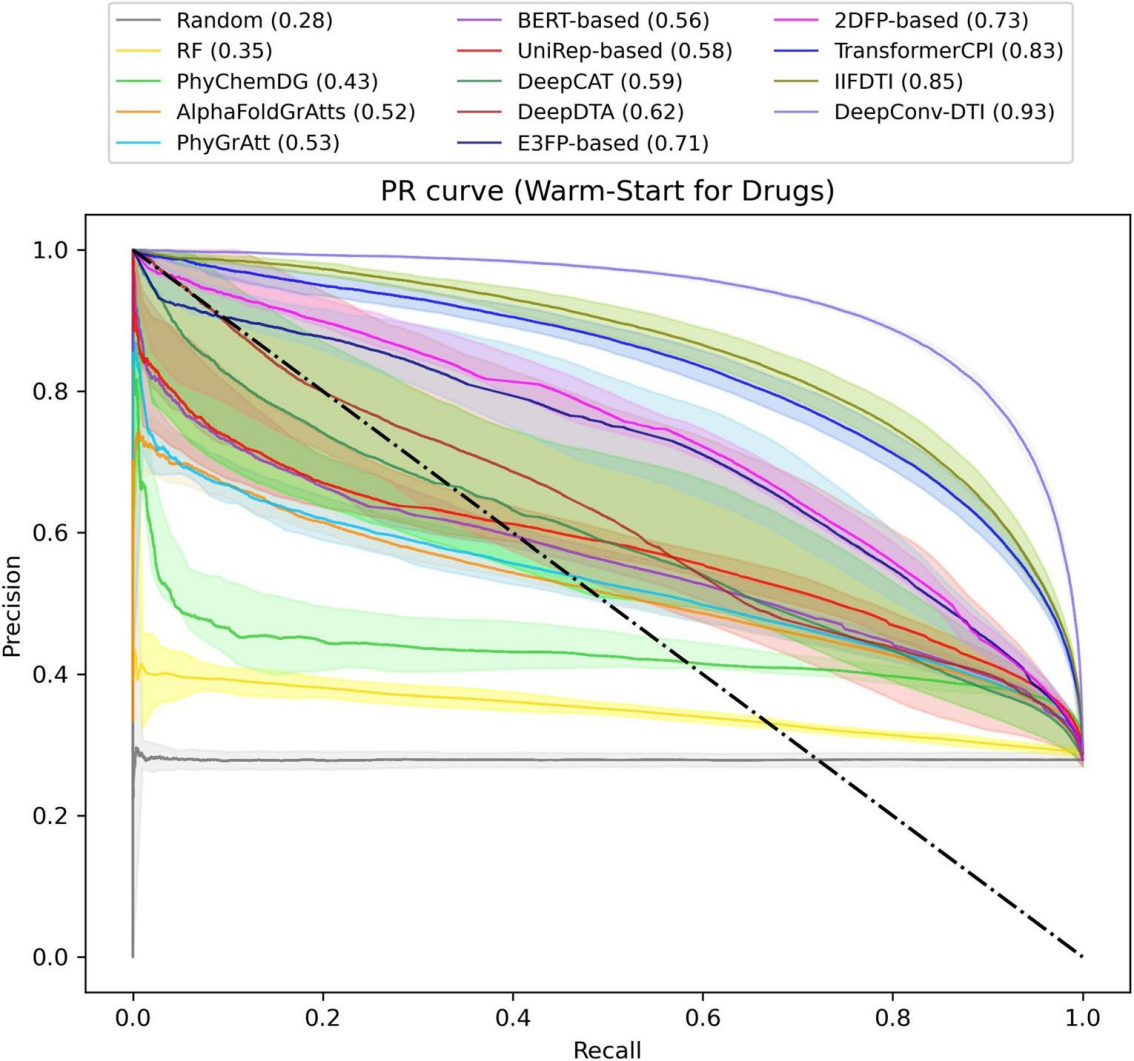
Comparison of PR curves for various models on the large aggregated dataset, split using the warm-start splitting scenarios for compounds

## Results

Figure 1 provides an overview of the general workflow of this study. The models utilize various types of neural network blocks designed to suit their respective input features (Table 1). Further details about the models are provided in  "Models" section. We assessed the models using five criteria: Accuracy, AUPR, AUROC, F1-score, and Matthews Correlation Coefficient (MCC). Given the datasets’ class imbalance, MCC is considered the most suitable criterion for model comparison.

### Performance evaluation on the large aggregated dataset

As explained in "Random Forest" section, the large aggregated dataset is formed through the consolidation of various datasets. Here, we employed two splitting scenarios, warm-start, and cold-start, both for compounds and targets. The warm-start splitting scenario for compounds involves dividing the dataset into training and test sets to evenly distribute the compounds across both sets. Consequently, the same compounds present in the training set are also included in the test set. In contrast, the cold-start splitting scenario for compounds mandates that the test set comprises compounds not found in the training set. Warm- and cold-start splits for targets follow the same logic as for compounds. Detailed information on the dataset splits can be found in "Evaluation approaches" section . To assess the models, we conducted 10-fold cross-validation. Supplementary Tables S1-S4 represent the composition of the training and test sets and the detailed information regarding the folds for the two splitting scenarios. As observed, the ratio of negative samples to positive samples in each fold ranged from 1.9 to 3 (Supplementary Fig. S3), and the average number of rotatable bonds in each fold ranged from 6.7 to 7.8.

In the warm-start scenario for compounds (Fig. 2 and supplementary Tables S5 and S6), DeepConv-DTI (MCC=0.79, AUPR=0.93) takes the top position with a significant lead over the second-ranking model, IIFDTI (MCC=0.68, AUPR=0.85). Subsequently, we observe a slight performance decline with models like TransformerCPI (MCC=0.65, AUPR=0.83), 2DFP-based (MCC=0.54, AUPR=0.73), and E3FP-based (MCC=0.53, AUPR=0.71), until we encounter a notable drop in performance with traditional models based on the SMILES notation of compounds, specifically DeepDTA (MCC=0.36, AUPR=0.62) and DeepCAT (MCC=0.30, AUPR=0.59). PhyGrAtt (MCC=0.28, AUPR=0.53), UniRep-based (MCC=0.28, AUPR=0.58), BERT-based (MCC=0.25, AUPR=0.56), AlphaFoldGrAtts (MCC=0.23, AUPR=0.52), PhyChemDG (MCC=0.21, AUPR=0.43), and RF (MCC=0.10, AUPR=0.35) models follow in subsequent ranks, respectively.

**Fig. 3:**
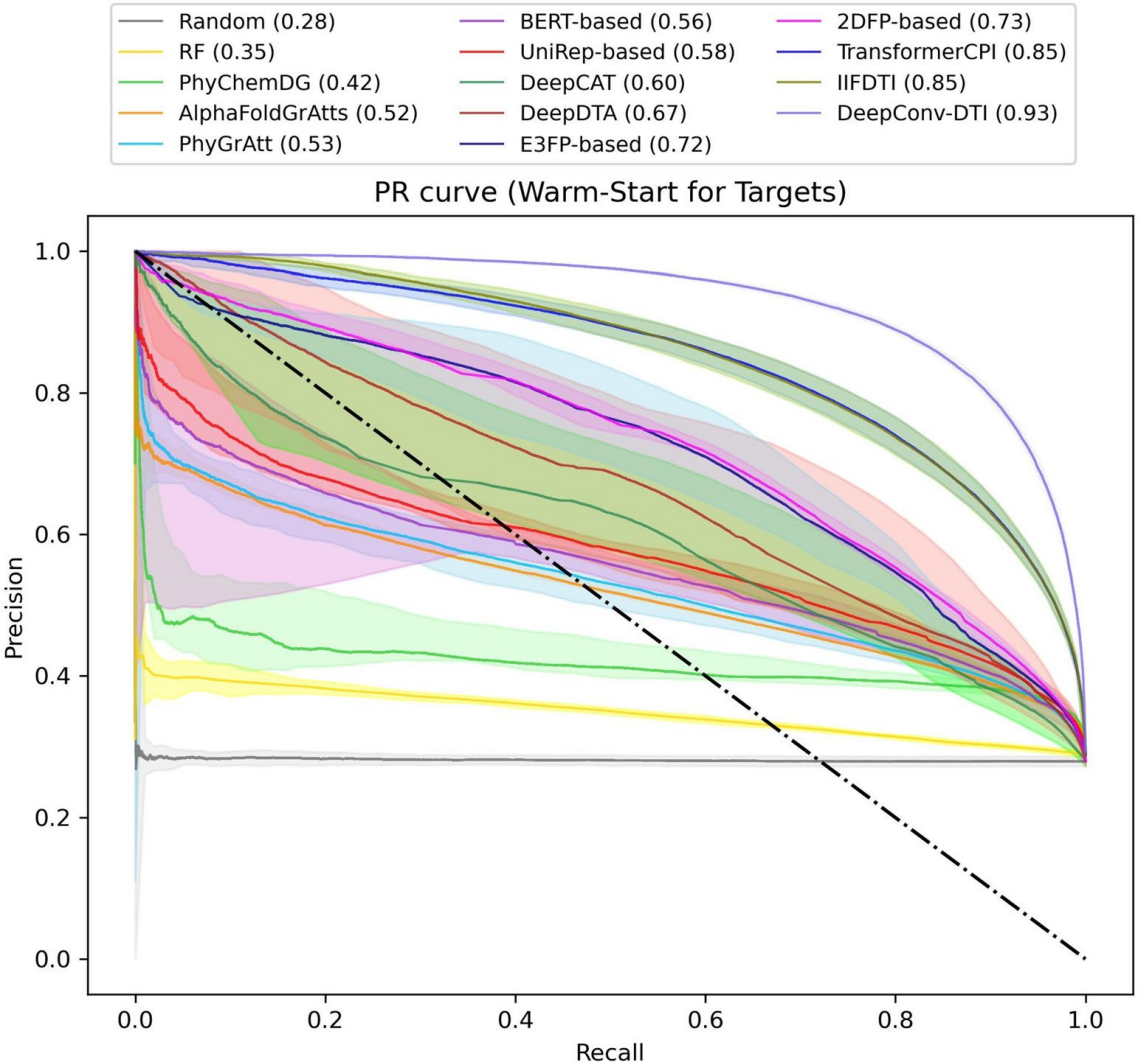
Comparison of PR curves for various models on the large aggregated dataset, split using the warm-start splitting scenarios for targets

In the warm-start scenario for targets (Fig. 3 and supplementary Tables S7 and S8), most of the models, including DeepConv-DTI, IIFDTI, 2DFP-based model, PhyGrAtt, E3FP-based model, PhyChemDG, and RF, exhibit similar performance to the warm-start scenario for compounds with slight differences. However, TransformerCPI (MCC=0.67, AUPR=0.85) and DeepDTA (MCC=0.44, AUPR=0.67), DeepCAT (MCC=0.38, AUPR=0.60), UniRep-based (MCC=0.30, AUPR=0.58), BERT-based (MCC=0.27, AUPR=0.56) demonstrate better performance compared to the warm-start scenario for compounds.

**Fig. 4:**
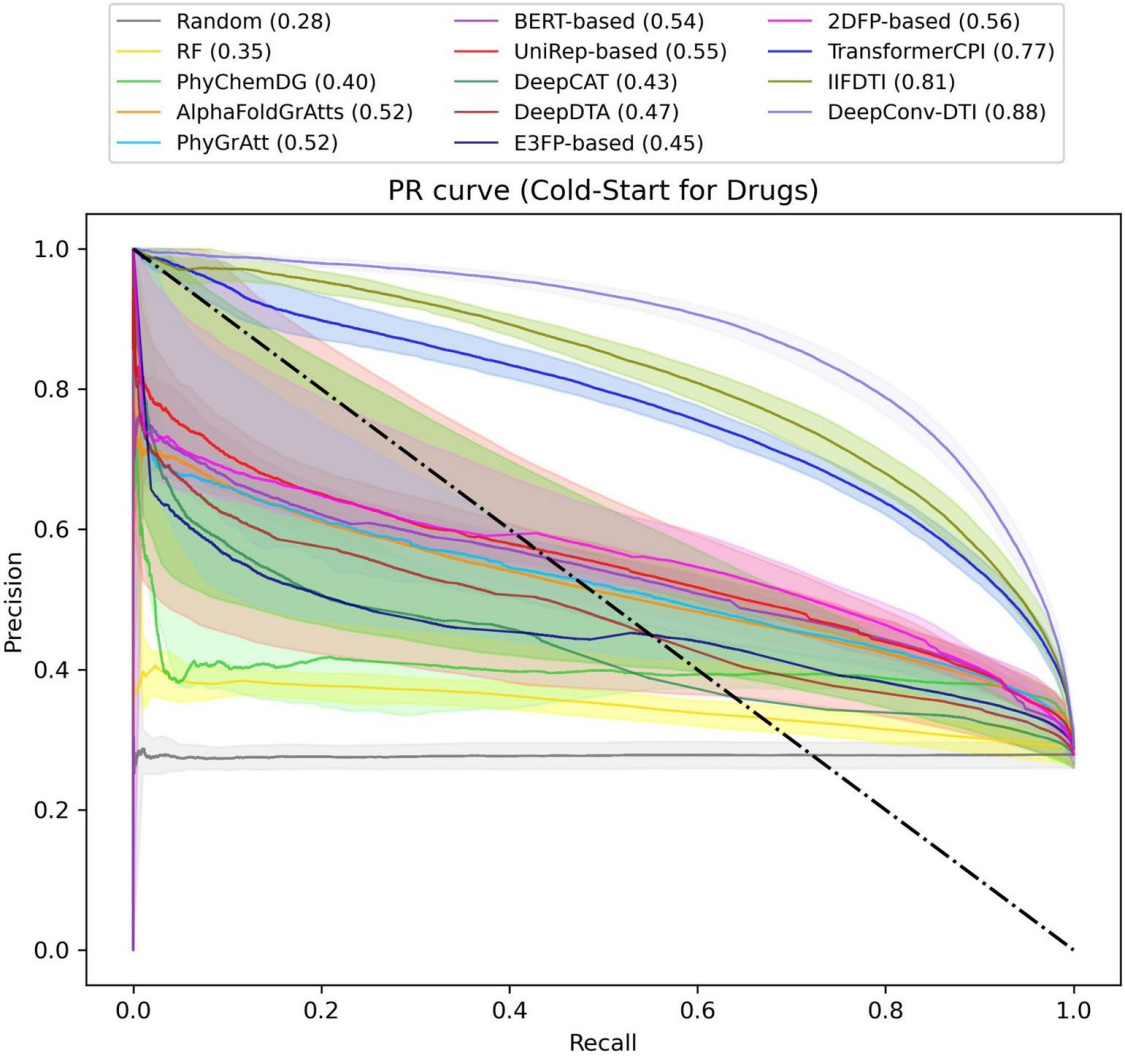
Comparison of PR curves for various models on the large aggregated dataset, split using the cold-start splitting scenarios for compounds

**Fig. 5:**
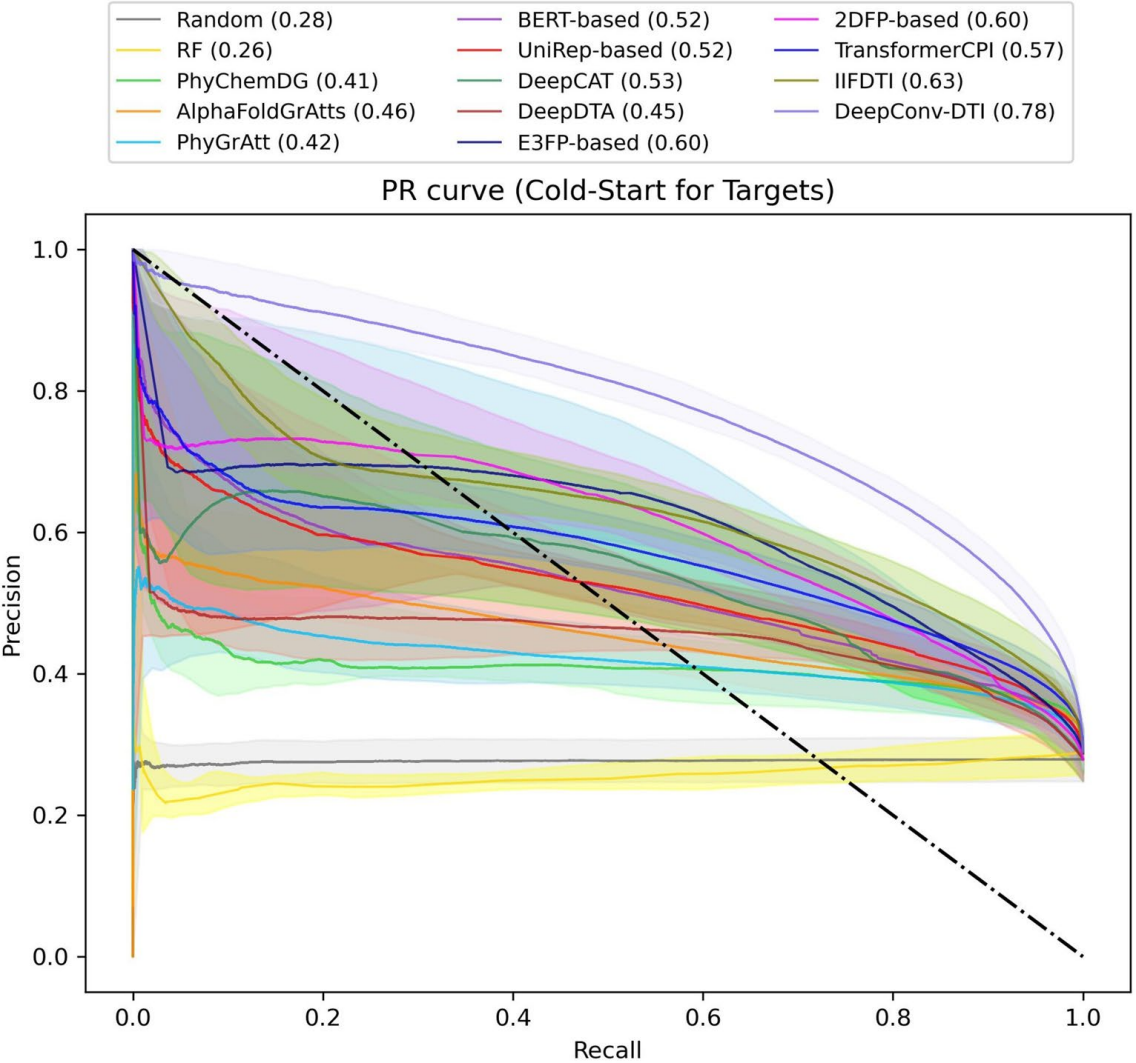
Comparison of PR curves for various models on the large aggregated dataset, split using the cold-start splitting scenarios for targets

In the cold-start scenario for compounds, as expected, there is a reduction in the performance of the various models compared to the warm-start splitting scenarios (Fig. 4 and supplementary Tables S11 and S12). However, many of the models, including DeepConv-DTI (MCC=0.71, AUPR=0.88), IIFDTI (MCC=0.62, AUPR=0.81), TransformerCPI (MCC=0.58, AUPR=0.77), UniRep-based (MCC=0.27, AUPR=0.55), BERT-based (MCC=0.26, AUPR=0.54), PhyGrAtt (MCC=0.25, AUPR=0.52), and AlphaFoldGrAtts (MCC=0.23, AUPR=0.52), perform better than in the case of a cold-start scenario for targets. Figure 5 highlights a significant performance disparity between the 2DFP-based (MCC=0.40, AUPR=0.56) and E3FP-based (MCC=0.26, AUPR=0.45) models. The E3FP-based model employs 3D drug fingerprints known as E3FP, taking multiple conformer vectors as input and extracting a representative compound vector through embedded convolutional layers.

The observed reduction in performance of the E3FP-based model in the cold-start scenario for compounds may be attributed to how conformers are extracted. In other words, the precision of conformer prediction plays a critical role in the creation of 3D drug fingerprints. This finding suggests that the representative embedding for compounds in the 2DFP-based model yields more informative results compared to the E3FP-based model. The analysis of the reduction disparity between warm-start and cold-start scenarios for compounds reveals significant decreases in performance for drug fingerprint-based models, with reductions of 49.3% and 26.3% for E3FP-based and 2DFP-based models, respectively. This is expected, as these models focus on the structure of compounds, making it more challenging to predict outcomes for unseen compounds. In contrast, protein structure-based models, such as PhyGrAtt and AlphaFoldGrAtts, show minimal reductions of 3.6% and 5%, respectively (Supplementary Fig. S4).

In the cold-start scenario for targets (Fig. 5 and supplementary Tables S9 and S10), it is evident that the performance of the transformer-based models, IIFDTI and TransformerCPI, and the structure-based models such as PhyGrAtt and AlphaFoldGrAtts experienced a significant decrease, 34.8%, 38.9%, 53.9%, and 44.5%, respectively, compared to the warm-start scenario for targets (Supplementary Fig. S5). This outcome is anticipated for AlphaFoldGrAtts and PhyGrAtt, as these models utilize the 3D structure of proteins, making it challenging for them to predict outcomes for unseen targets. In contrast, compound structure-based models, such as those utilizing 2DFP and E3FP, demonstrate greater generalizability, with reductions of 17.6% and 10.4%, respectively, and are less impacted by unseen targets. Surprisingly, the E3FP-based model (MCC=0.46, AUPR=0.60) secures the third position, surpassing TransformerCPI. Besides, the models based on language model learned embeddings, such as UniRep-based and BERT-based models, exhibit minimal reduction in performance. This observation supports the notion that the learned embeddings derived from language models encompass more informative protein features than other models, enabling them to maintain their predictive capabilities for compound binding with unseen amino acid sequences. This finding aligns with current research in the field [17]. Additionally, Chen et al. proposed a model that emphasizes the crucial role of using BERT-based embeddings in CTI prediction [14].

While we have several models based on trainable target embeddings, there exist multiple approaches to capture these features. The remarkable results obtained from DeepConv-DTI (MCC=0.59, AUPR=0.78) suggest that the global sliding feature capturing method provides more informative features compared to other models relying on trainable embeddings. DeepConv-DTI employs multiple windows of varying sizes to capture the characteristics of various possible adjacent amino acids by sliding the windows from the top to the bottom of the embeddings matrix.

For each scenario, to demonstrate that the models outperform random predictions, we assigned random values and calculated MCC and AUPR, resulting in values of 0.00 and 0.28, respectively. The results obtained from the PhyChemDG model emphasize the significance of using a transformer-encoder to capture informative features of the target. PhyChemDG is a variant of TransformerCPI, where we replace the transformer-encoder component of the target side with fully connected (FC) layers that receive input from the physicochemical properties. However, the results indicate that this modification leads to a decrease in MCC performance of 0.17 to 0.44. Supplementary Fig. S6-S9 show the AUROC of the models based on different splitting scenarios.

### Evaluating the target representation vectors

Apart from assessing the performance of the models on the aggregated dataset, we also evaluated the representations of various learned models in terms of target clustering. This was achieved by visualizing the targets in the dataset in a 2D space using t-distributed stochastic neighbor embedding (t-SNE) projection. To clearly distinguish between different types of targets we categorized them into two classes: enzymes and non-enzymes, represented by darker and lighter colors respectively (Supplementary Fig. S10–S15). Within the non-enzyme category, we further stratified targets into epigenetic regulators, ion channels, membrane receptors, transcription factors, and transporters. Enzyme targets were categorized into hydrolases, oxidoreductases, proteases, transferases, and other enzymes. As depicted in Supplementary Fig. S10–S15, the targets exhibit distinct clusters based on the representations of the UniRep-based and BERT-based models. Particularly noteworthy is the ability of the UniRep-based representations to form more refined clusters, like those of membrane receptors and transporters, compared to the BERT-based representations. This distinction might underlie the UniRep-based model’s superior performance compared to the BERT-based model over the large aggregated dataset. With regards to the physicochemical properties-based features derived from the DeepCAT and E3FP-based models, we still discern some clusters, particularly for two non-enzyme targets: ion channels and epigenetic regulators. Nevertheless, for the remaining targets, distinct clustering is absent. The t-SNE plots of target representations from the other models lack any discernible patterns and do not exhibit distinguishable clusters. Therefore, learned embeddings from BERT and UniRep exhibit distinctive features as representations for targets. This could contribute to the consistent performance of UniRep-based and BERT-based models within a specific range across both cold-start and warm-start scenarios.

### Performance evaluation of models on distinct datasets

**Fig. 6:**
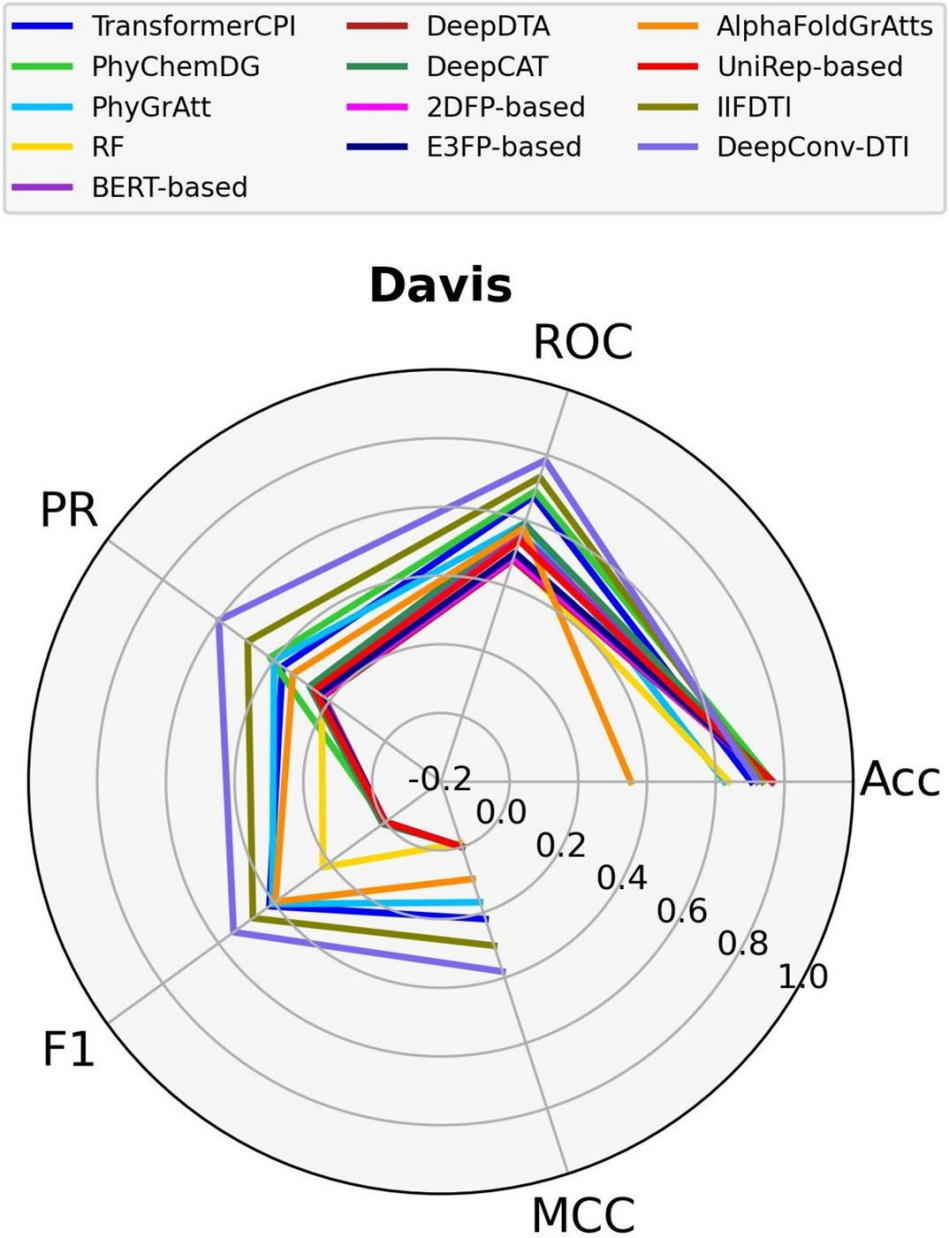
Comparison of models across the small (Davis) dataset

**Fig. 7:**
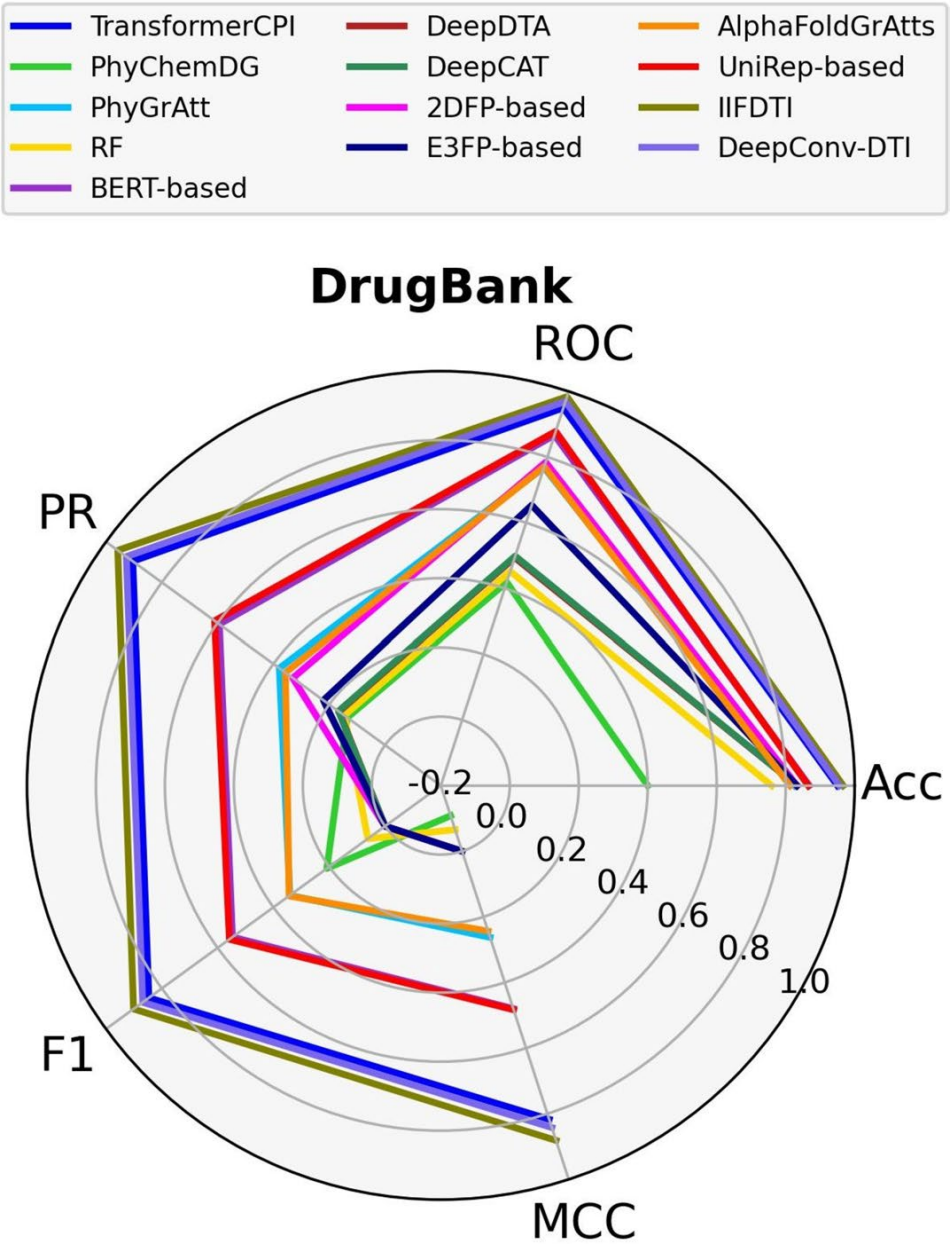
Comparison of models across the medium (DrugBank) dataset

Given that the various datasets employ distinct experimental methods to obtain the binding affinity value for each CTI, it is valuable to compare the models across separate datasets. In this study, we evaluate the performance of the models across three distinct datasets–Davis (small), DrugBank (medium), and KiBA (large)–considering factors such as the number of positive and negative CTIs, the number of involved proteins, the number of involved compounds, and the experimental methods used. The dataset sizes are determined by the number of positive and negative CTIs. Notably, the Davis dataset includes only 72 compounds and 442 proteins. In contrast, the DrugBank dataset features over 4,000 proteins and approximately 14,000 compounds. The KiBA dataset contains a high frequency of compounds (over 52,000) but only 467 proteins.. All three datasets are split based on a random cold-start splitting for compounds scenario. Detailed information about the datasets can be found in Supplementary Table S13, and the experimental methods are described in "Datasets" section. Figs. 6 and 7 display the performance of the models based on five different criteria: Accuracy, AUPR, AUROC, F1-score, and MCC on a spider plot.

In Fig. 6, it is evident that although we achieved promising results for the transformer-based models, IIFDTI (MCC=0.30, AUPR=0.50) and TransformerCPI (MCC=0.22, AUPR=0.37), DeepConv-DTI (MCC=0.38, AUPR=0.60), still outperforms both models on the Davis dataset. Additionally, the structure-based models, such as PhyGrAtt (MCC=0.17, AUPR=0.40) and AlphaFoldGrAtts (MCC=0.10, AUPR=0.33), which utilize GAT layers, secure the third position. While the other models may exhibit good accuracy, their MCC values are zero or, in some cases, negative. These results suggest that these models tend to predict the output labels of the majority class (0 in this case) on the Davis dataset.

As we move from the Davis dataset to the DrugBank dataset (Fig. 7), there is a significant improvement in the performance of transformer-based models, specifically IIFDTI (MCC=0.86, AUPR=0.96) and TransformerCPI (MCC=0.82, AUPR=0.90). We observed a similar improvement for the DeepConv-DTI model (MCC=0.84, AUPR=0.92). However, it drops from the first position to the second position among the models. Additionally, we observed enhancements in the performance of structure-based models, such as PhyGrAtt (MCC=0.26, AUPR=0.38) and AlphaFoldGrAtts (MCC=0.24, AUPR=0.36). Furthermore, the performance of learned embeddings-based models, namely UniRep-based (MCC=0.48, AUPR=0.61) and BERT-based (MCC=0.48, AUPR=0.60), emphasizes the importance of learned embeddings as target input features. These findings are consistent with the results reported by Guvenilir et al. [17] and Chen et al. [14].

**Fig. 8:**
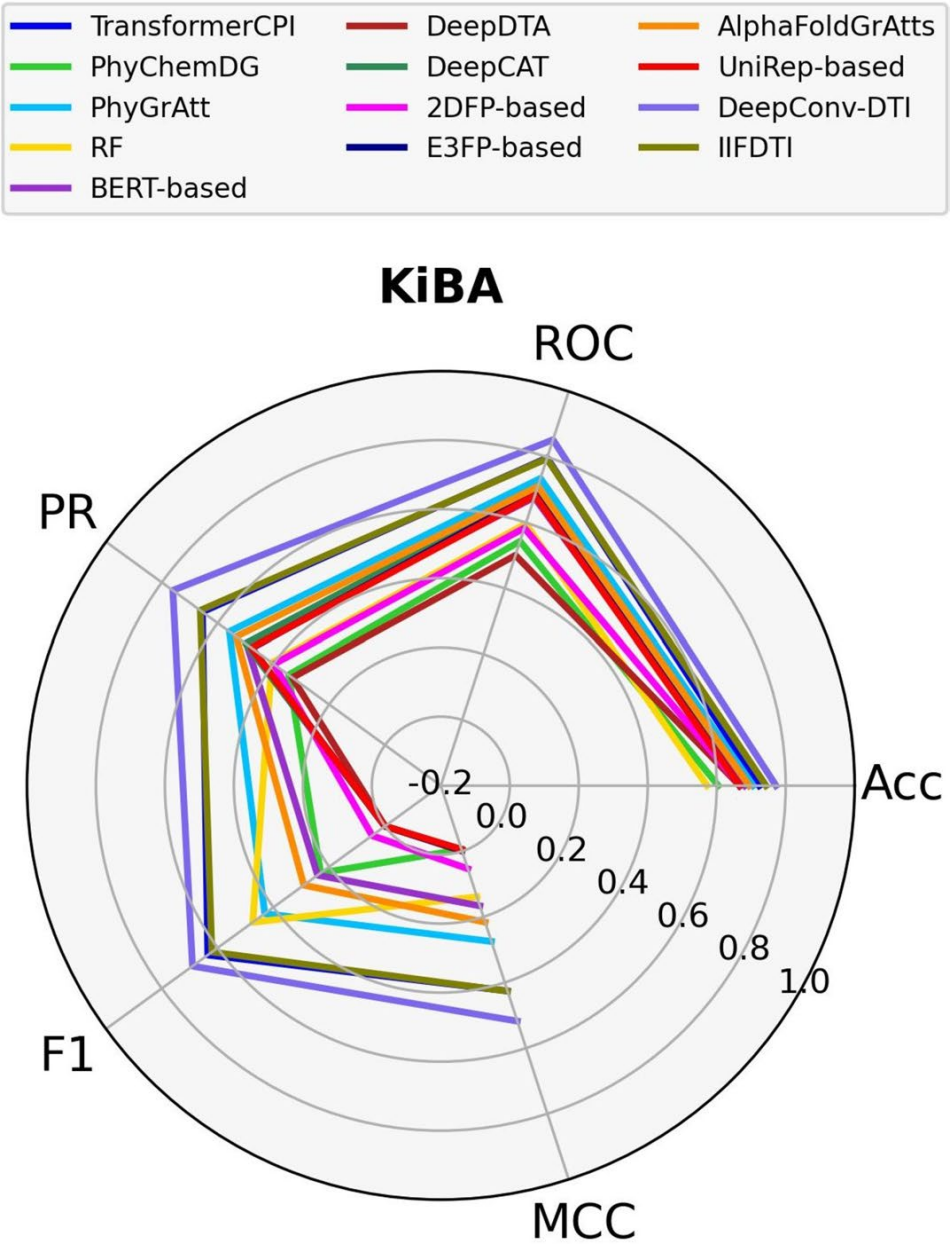
Comparison of models across the large (KiBA) dataset

In Fig. 8, the performance of the models on the KiBA dataset is illustrated. It is notable that certain models, including DeepConv-DTI (MCC=0.52, AUPR=0.76), IIFDTI (MCC=0.43, AUPR=0.66), TransformerCPI (MCC=0.42, AUPR=0.65) and BERT-based (MCC=0.17, AUPR=0.50), exhibit a reduction in performance compared to the DrugBank dataset. However, the performance of structure-based models, such as PhyGrAtt (MCC=0.28, AUPR=0.56) and AlphaFoldGrAtts (MCC=0.22, AUPR=0.53), remains relatively stable across all dataset sizes. Based on our observations, DeepConv-DTI consistently delivers outstanding results across three diverse datasets of varying sizes and quality. The performance values of the models for datasets of varying sizes are shown in supplementary Tables S14-S16.

### Training on wild-type targets and testing on mutated targets

We trained the models using a dataset that comprises wild-type targets and subsequently tested their performance on a separate dataset consisting of mutated targets.

**Fig. 9:**
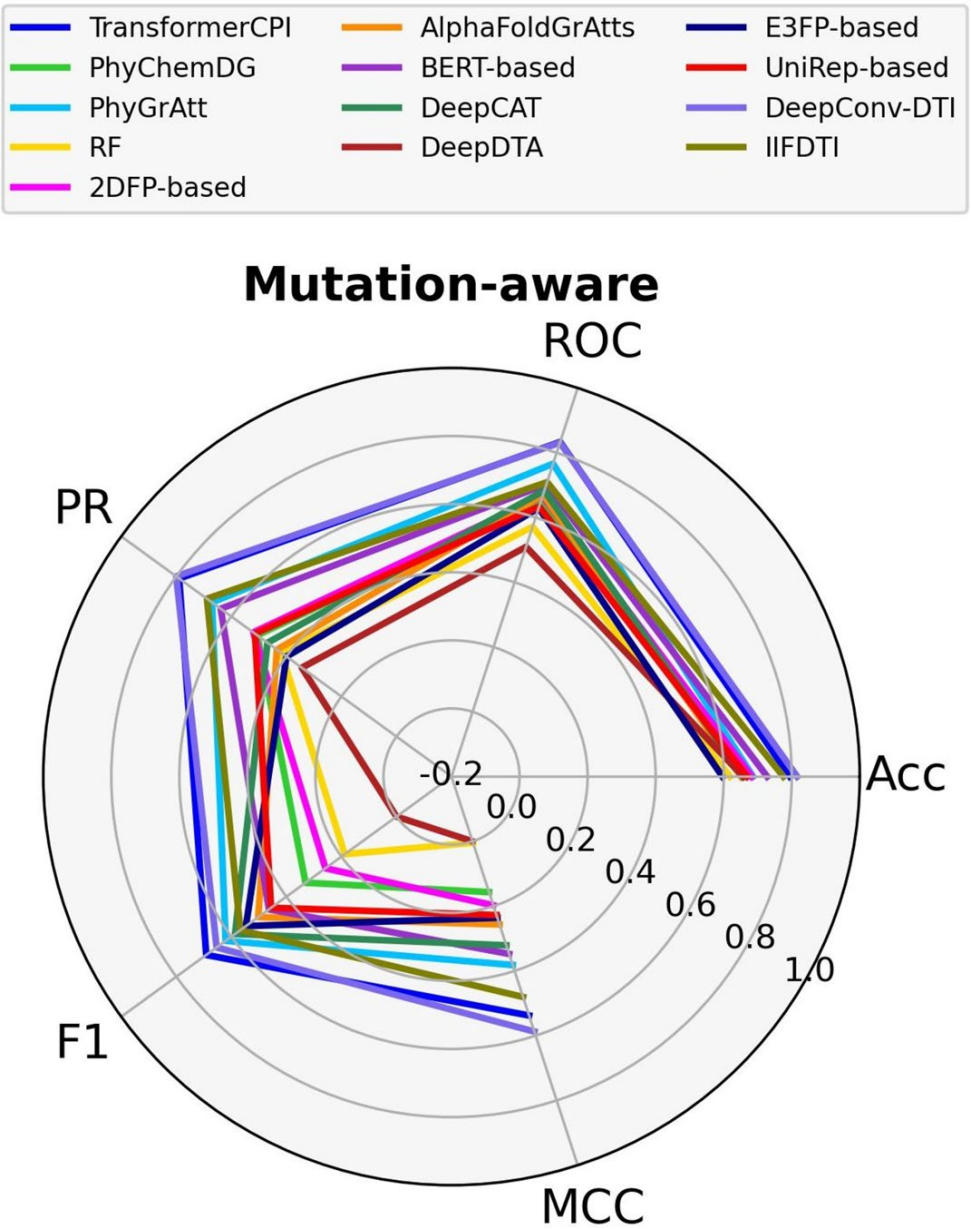
Comparison of models across the mutation-aware dataset

As shown in Fig. 9, the DeepConv-DTI model (MCC=0.59, AUPR=0.80), which employs global convolutional blocks, outperforms the other models. The transformer-based models with encoder-decoder blocks, such as TransformerCPI (MCC=0.54, AUPR=0.79) and IIFDTI (MCC=0.48, AUPR=0.69), exhibit the second-highest performance. As expected, the structure-based models, PhyGrAtt (MCC=0.38, AUPR=0.67) and AlphaFoldGrAtts (MCC=0.26, AUPR=0.43), consistently demonstrate promising results across the dataset.

In the fourth position, the learned embeddings-based models, BERT-based (MCC=0.35, AUPR=0.64) and UniRep-based (MCC=0.23, AUPR=0.51), exhibit good predictive performances. The models utilizing physicochemical properties, such as DeepCAT (MCC=0.32, AUPR=0.47), E3FP-based (MCC=0.24, AUPR=0.40), and 2DFP-based (MCC=0.20, AUPR=0.52), achieved satisfactory performances. It is noteworthy, however, that the models using atom interaction networks for compounds outperform the drug fingerprint-based models. On the other hand, the performance of the DeepConv-DTI model indicates that the influence of the compound descriptor can be mitigated through improved target feature extraction. The superiority of this model could be attributed to its ability to capture features globally rather than relying on local feature extraction. Lastly, comparing the performance of TransformerCPI with that of PhyChemDG (MCC=0.16, AUPR=0.50) highlights the superiority of the target transformer-encoder component in the TransformerCPI architecture. The performance values of the models for the mutation-aware dataset are shown in supplementary Table S17.

### The influence of rotatable bonds on CTI predictions

**Fig. 10:**
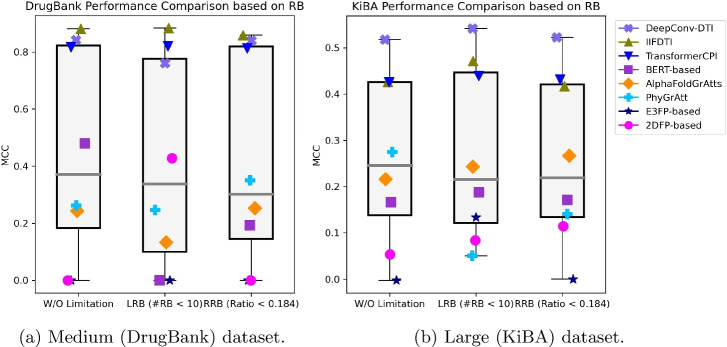
Performance comparison of eight models over DrugBank and KiBA datasets, under three filtering scenarios: (left) no limitations, (center) compounds with $\le 10$ rotatable bonds, and (right) compounds with rotatable bond ratio $\le 0.184$

In this section, we want to evaluate how the models perform when the dataset includes or excludes compounds with a considerable number of rotatable bonds. To evaluate the performance of the models under the condition of limited rotatable bonds, we utilized two datasets of different sizes: the medium DrugBank dataset and the large KiBA dataset (Supplementary Table S13). We restricted this comparison to the eight models that exhibited the best performances in the previous sections. In Fig. 10, the first two columns of the box plots illustrate the performance comparison of the models under two scenarios: without any limitation on the number of rotatable bonds (left boxplot) and with a limited number of rotatable bonds (middle boxplot), assessed by the MCC metric. Supplementary Tables S14 and S15 present five different performance metrics for various models on the rotatable bond-aware datasets.

When applying the limited rotatable bonds (LRB) criterion to the DrugBank dataset, we observed marginal improvements for TransformerCPI and substantial enhancements for the 2DFP-based model. However, for the remaining models, there were no notable changes, and in some cases, performance worsened.

In the KiBA dataset, the number of models showing slight improvements expanded to seven, specifically the 2DFP-based, E3FP-based, AlphaFoldGrAtts, BERT-based, TransformerCPI, IIFDTI, and DeepConv-DTI models. Conversely, the PhyGrAtt performance significantly declined.

Additionally, we assessed the models’ performance using a ratio-based rotatable bonds (RRB) criterion, as explained in "Evaluation approaches" section. The third column of Fig. 10a and b illustrates the performance of the models when using the RRB datasets.

When we applied the RRB criterion to the DrugBank dataset, we observed slight improvements in the performance of two graph attention-based models, PhyGrAtt and AlphaFoldGrAtts. However, for most of the models, there were no significant changes, and in some cases, performance even declined. This pattern persisted when applying RRB to the KiBA dataset. Much like the DrugBank dataset, the majority of models exhibited no significant changes when RRB was applied to the KiBA dataset. Notable improvements were observed for the 2DFP-based model and AlphaFoldGrAtts.

Overall, these results suggest that at least in our settings the number of rotatable bonds seems to have limited to no impact on model performance.

### The execution time of the models

In addition to the performance of the models, the execution time for the training and test phases is of utmost importance. Here, we trained the models on 259,117 samples, tested on 5,819 CTI positive and negative samples, and computed the execution time for each phase separately. The models were trained and tested on NVIDIA A100 SXM4 40 GB GPUs, and the execution times for training and inference are reported in Table 2.

**Table 2:** Execution time of the models

Rank	Models	One Epoch Time (seconds)	Inference Time (seconds)
1	BERT-based	148.7	0.53
2	UniRep-based	169.6	0.57
3	DeepConv-DTI	217.9	1.45
4	E3FP-based	326.4	0.53
5	DeepDTA	450.9	0.49
6	DeepCAT	455.6	0.49
7	PhyGrAtt	1464.3	2.37
8	AlphaFoldGrAtts	1633.4	2.91
9	2DFP-based	1723.2	14.7
10	IIFDTI	2427.2	54.0
11	PhyChemDG	23557.2	147.1
12	TransformerCPI	23561.8	148.0

The models are sorted based on the average execution time per epoch during the training phase, and the numbers are given in seconds. As depicted in Table 2, models utilizing pre-trained embeddings, such as the BERT-based and UniRep-based models, demonstrate the fastest performance. Convolutional neural network-based models, including DeepConv-DTI, E3FP-based, DeepDTA, and DeepCAT, exhibit swift training and inference. The third position is occupied by GAT-based models, such as PhyGrAtt and AlphaFoldGrAtts, which are constructed on graphs of residue (or atom) interactions. The models with the longest execution times are the transformer-based models, characterized by a high number of computational steps and parameters.

Overall, while the top three models, IIFDTI, TransformerCPI, and DeepConv-DTI, show competitive prediction performance, the execution time for training and inference decisively underscores the superiority of the DeepConv-DTI model.

**Fig. 11:**
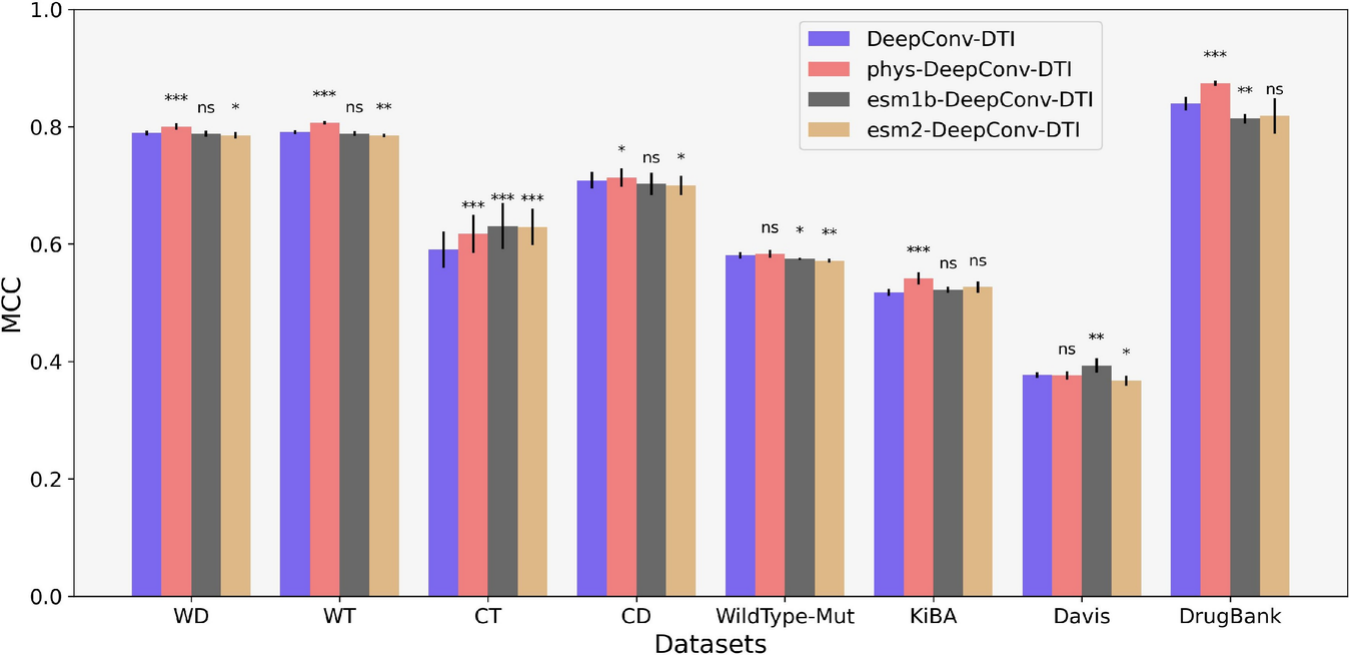
Comparison of the performance of the DeepConv-DTI model in four scenarios: initializing the trainable embeddings using normalized physicochemical properties (Phys-DeepConv-DTI), using randomly-initialized embeddings (the original version of DeepConv-DTI), utilizing ESM-1b and ESM-2 as protein representations (esm1b-DeepConv-DTI and esm2-DeepConv-DTI, respectively). The symbols above each bar indicate the p-value significance levels when comparing DeepConv-DTI with its other versions

### Performance improvement through pre-trained embeddings and physicochemical properties

In our previous evaluations, we demonstrated that DeepConv-DTI outperforms other models across most datasets. As detailed in "Methods" section, this model utilizes randomly-initialized trainable embeddings for each amino acid, which undergo training in the training phase. The trainable property of the embeddings aims to modify them to incorporate informative and representative features crucial for binding. Our prior results across the different target representations suggest, however, that an initialization based on physicochemical properties of the aminoacids or on reconstruction (e.g. BERT) should be beneficial. This prompted us to modify DeepConv-DTI to initialize the embeddings using normalized physicochemical properties of amino acids instead of random values obtained from a uniform distribution and named this model Phys-DeepConv-DTI. Additionally, we employed two recently published and widely used pre-trained embeddings, ESM-1b [19] and ESM2 [20], as protein representations for DeepConv-DTI, naming the models esm1b-DeepConv-DTI and esm2-DeepConv-DTI, respectively.

We independently trained and evaluated the different versions of the DeepConv-DTI model across each dataset ten times to obtain reliable performance measures. As illustrated in Fig. 11, Phys-Conv-DTI demonstrates significantly better performance than DeepConv-DTI and its other versions across most datasets in terms of MCC, including warm-start for compounds and targets, cold-start for compounds, as well as KiBA and DrugBank datasets. Conversely, the value of ESM-1b becomes particularly apparent when using the cold-start for targets dataset, indicating that ESM-1b is a more effective choice for predicting interactions between compounds and previously unseen targets. In summary, we were able to improve the best model based on our systematic evaluation of beneficial compound and target representations and network architectures. We believe this approach holds great promise for the further optimization of deep learning-based CTI prediction models.

### Comparison of models using the label reversal experiments

**Fig. 12:**
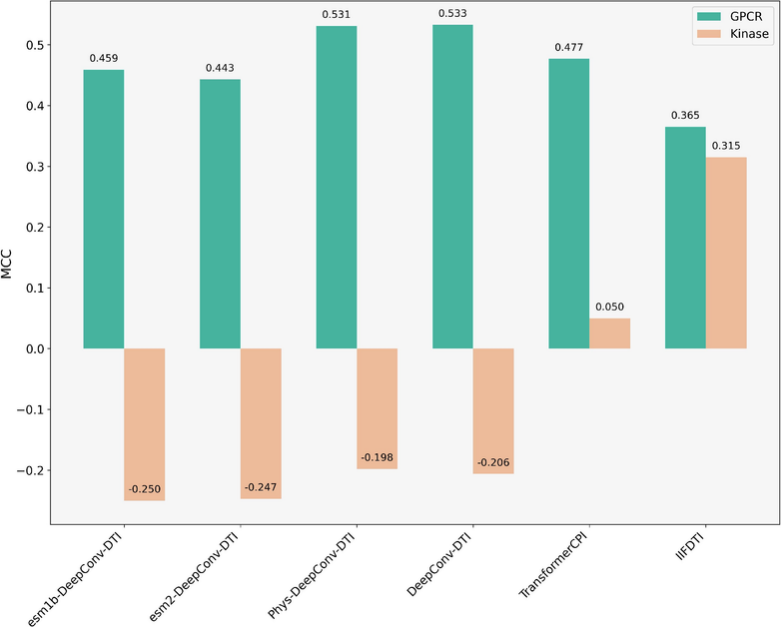
Comparison of models across the label reversal datasets

The hidden compound bias in a model occurs when it predicts the CTI based solely on compound-specific clues and patterns, rather than on the desired interaction features. This issue has been observed in two recent studies [21, 22], and Chen et al. [6] proposed a label reversal experiment to determine if a CTI prediction model faces a similar problem. They created two datasets, GPCR and Kinase, based on the GLASS [23] and KiBA databases, respectively. In the label reversal scenario, a compound present in one class in the training set appears exclusively in the opposite class in the test set. Here, we compare the top three models alongside various versions of DeepConv-DTI. Figure 12 and Supplementary Fig. S16 present the performance of the models on the label reversal dataset, measured by MCC and F1 score, respectively.

As shown in Fig. 12, DeepConv-DTI (MCC=0.533) demonstrates the best performance on the GPCR dataset, while IIFDTI (MCC = 0.315) outperforms other models on the Kinase dataset. The figure also demonstrates a significant decline in model performance on the Kinase dataset compared to the GPCR dataset, particularly for DeepConv-DTI and its variants. These models perform worse in predicting CTIs with reversed labels, yielding results below the random prediction baseline, which has an MCC of 0.0. Consequently, we analyzed the frequency of compounds contributing to negative or positive CTIs in the training and test sets of each dataset separately. We observed that in the Kinase dataset, a compound appears, on average, 44 times during the training phase with either a negative or positive label, but only 7 times in the test set with the opposite label. In contrast, in the GPCR dataset, each compound appears on average once or twice during the training phase and once in the test set (Supplementary Fig. S17).

When a model encounters a compound multiple times with a constant output label, its parameters are trained to predict that label more confidently when the same compound’s features appear in the test set. This makes it more difficult for the model to accurately predict a CTI containing the compound with a reversed label.

Interestingly, the two transformer-based models, TransformerCPI and IIFDTI, achieve better results on the Kinase dataset compared to the others. These findings suggest that transformer-based models have a greater ability to capture interaction features, rather than relying solely on compound features.

## Discussion

Although different models and input features exhibit varying performances across different datasets, the performance of each model may depend on specific structural, behavioral, and characteristic features of proteins and compounds. Consequently, these features can complement each other and enhance the overall performance of a new hybrid model. For example, the features captured by AlphaFold represent the target’s structure, while BERT-based or UniRep-based input features encode the amino acid sequence order. Additionally, the physicochemical properties of amino acids can describe their characteristics such as polarity, hydrophilicity, hydrophobicity. Notably, based on our findings, a transformer-based model utilized for both compounds and targets proves to be one of the most successful. Hence, a transformer-based architecture can effectively capture informative features from sequential data. On the other hand, the structure-based models that utilize AlphaFold to predict 3D structures and atoms’ interaction networks demonstrate consistent performance across datasets of various sizes.

One of the disadvantages of transformer-based models like TransformerCPI and IIFDTI is the extended training time required, especially for the large aggregated dataset. Based on our findings, these models can be efficiently trained and evaluated on small and medium datasets. However, regardless of the data-splitting scenario, the results on the larger datasets indicate that they do not surpass other models, especially convolutional-based models like DeepConv-DTI. While the transformer-based models achieve high performance in comparison to most models, they may not effectively capture global target features as comprehensively as the global convolutional-based model, DeepConv-DTI. Furthermore, in the case of compound descriptors, the representation by 2D atom interaction networks, which assigns a vector of chemical and structural properties to each atom, consistently achieves high performance compared to other representations.

Regarding the influence of the rotatable bonds, DeepConv-DTI, IIFDTI, and TransformerCPI display lower sensitivity to the rotational bonds of compounds. Conversely, the performance of the 2DFP-based model and models utilizing GAT layers, such as AlphaFoldGrAtts and PhyGrAtt, is notably affected by the presence of rotatable bonds. Regardless of the target input features, it’s important to emphasize that the method of capturing and refining these features can be more effective than the features themselves in predicting CTIs. Our study revealed that globally refining target features using simple convolutional blocks can be more effective than complex transformer-based models. Global feature capture encodes the characteristics of various groups of adjacent residues, leading to more precise predictions for mutated targets. Additionally, there are multiple CTI models, such as DeepConv-DTI, DeepDTA, and IIFDTI, that utilize randomly initialized trainable embeddings. According to our findings, the performance of future models employing trainable embeddings can be enhanced by initializing them using normalized physicochemical properties.

The performance of the models on label reversal datasets indicates that transformers, as a neural network architecture, are a strong choice due to their ability to capture interaction features rather than relying solely on compound patterns. In other words, transformers are well-suited to mitigating the issue of hidden compound bias. Transformer-based models employ a quadratic attention mechanism, which requires quadratic memory and can be challenging for longer sequences. One potential solution to this issue is the use of novel transformer-based models like BigBird. These models replace the quadratic attention mechanism with a combination of window-based attention, random attention, and selective global attention, resulting in linear memory requirements [24].

In conclusion, it is not only feasible but also advantageous to prefer models that not only predict accurately but also operate more efficiently in terms of training and inference times. Another crucial factor to consider is the memory required for both the parameters of a model and its computations. Models that demand less memory and exhibit faster processing contribute to decreasing the overall cost of resources. In this context, convolutional neural networks rely more on the size of kernels than on the size of input features. As a result, they can utilize fewer parameters. On the contrary, transformers necessitate a considerable number of parameters and involve numerous computational steps. With enhanced models, such as DeepConv-DTI, we can efficiently screen more compounds against disease-relevant targets in a shorter timeframe than classical molecular docking workflows.

## Methods

### Datasets

In this study, four datasets were utilized to compare the performance of various models, namely, KiBA, DrugBank, Davis, and D3R datasets.

**KiBA**: The Kinase Inhibitor Bioactivity dataset (KiBA) dataset [25] consists of 52,498 chemical compounds and 467 kinase targets. It utilizes IC50 values to determine the binding affinity between compounds and targets. The strength of the binding is represented by the KiBA score, where a higher score indicates a lower binding affinity. In this study, we adopted a threshold of 3 for the KiBA score, categorizing compound-target pairs with scores less than or equal to 3 as “*hits*” and those above 3 as “*non-binds*”. Overall, the KiBA dataset comprises 243,251 compound-target records, with 79,787 positive samples indicating binding and 163,464 negative samples representing non-binding interactions.

**DrugBank**: The DrugBank dataset (version 28.03.2022) is a comprehensive collection of data that includes various information such as the molecular weight of compounds, signal regions of targets, transmembrane regions of targets, adverse effects, compound-target interactions, compound-compound interactions, and more. The version used in this study encompasses 14,624 compounds and 4,889 targets. The dataset consists of a total of 21,243 positive compound-target interactions, indicating binding between compounds and targets. However, it does not contain any negative samples, representing non-binding interactions.

**Davis**: The Davis dataset [26] is used to measure the binding affinity of compound-target pairs using the equilibrium dissociation constant (Kd). In this dataset, a higher Kd value indicates a lower binding affinity between the compound and target. For this study, we set a threshold of $10 \mu M$ to classify the compound-target pairs. Compound-target pairs with a Kd value less than $10 \mu M$ are considered as hits, indicating a binding interaction, while those with a Kd value greater than or equal to $10 \mu M$ are classified as ’non-bind’, representing a lack of binding. The Davis dataset consists of 72 chemical compounds and 442 kinase targets. Overall, the dataset comprises 31,824 compound-target pairs, with 9,424 classified as positive samples (hits) and 22,400 as negative samples (non-binds).

**D3R**: The Drug Design Data Resource (D3R) dataset comprises data from four grand challenges. The dataset contains a total of 2,200 compound-target pairs, consisting of 1,769 positive samples and 431 negative samples. The D3R dataset is available at https://drugdesigndata.org/about/datasets.

#### Data preprocessing

Supplementary Fig. S18 presents the histogram depicting the distribution of compounds and targets based on the length of the SMILES notations of compounds and the number of amino acids, respectively. To ensure a fair comparison of models, we excluded targets with more than 1400 amino acids and compounds with more than 350 SMILES characters, based on the observed distribution. Additionally, it is crucial to exclude targets that do not have a valid 3D structure predicted by AlphaFold, as well as compounds for which E3FP is unable to obtain their 3D fingerprints.

After applying all the aforementioned limitations and removing duplicate records, we obtained a final dataset consisting of 337,526 unique records, including 240,055 negative samples and 97,471 positive samples. This combined dataset encompasses 4700 targets and 58,122 compounds.

The preprocessed Davis dataset consists of two subsets: one involving mutated targets and the other involving wild-type targets. The dataset contains a total of 3,168 records, with 1351 positive samples and 1,817 negative samples, for the mutated targets subset. For the wild-type targets subset, there are 25,698 records, with 5,471 positive samples and 20,227 negative samples. In the dataset, there are 335 wild-type kinase targets and 44 mutated targets. Additionally, the dataset includes a total of 72 compounds. The preprocessed D3R dataset comprises 467 records of compound-mutated target interactions, with 461 positive samples and 6 negative samples. Additionally, the dataset includes 1733 records involving wild-type targets, with 1308 positive samples and 425 negative samples. The preprocessed KiBA dataset comprises 233,911 samples, including 157,295 negative samples and 76,616 positive samples. Finally, the preprocessed DrugBank dataset contains 17,207 samples, but it lacks negative samples. Therefore, in the next part, we will discuss how we generated negative samples for this dataset.

### Gold-standard Datasets

We assessed different models using our gold-standard datasets, including the large aggregated datasets, mutation-aware dataset, rotational bonds-aware dataset, and separate datasets with varying sizes. Each gold-standard dataset is described in the following sections separately. The datasets are derived from four widely used databases: KiBA, DrugBank, Davis, and D3R. The details about each dataset can be found in "Datasets" section. Additionally, since DrugBank lacks negative samples, we have developed an approach to generate them using the Tanimoto distance between compounds, as described in the following section.

#### Negative samples generation

Most previous studies have attempted to randomly assign compound-target pairs that have not been observed in the positive samples as negative samples. In this study, we aim to select compound-target pairs that do not exist in the DrugBank dataset and are as dissimilar as possible to the positive pairs.

**Fig. 13:**
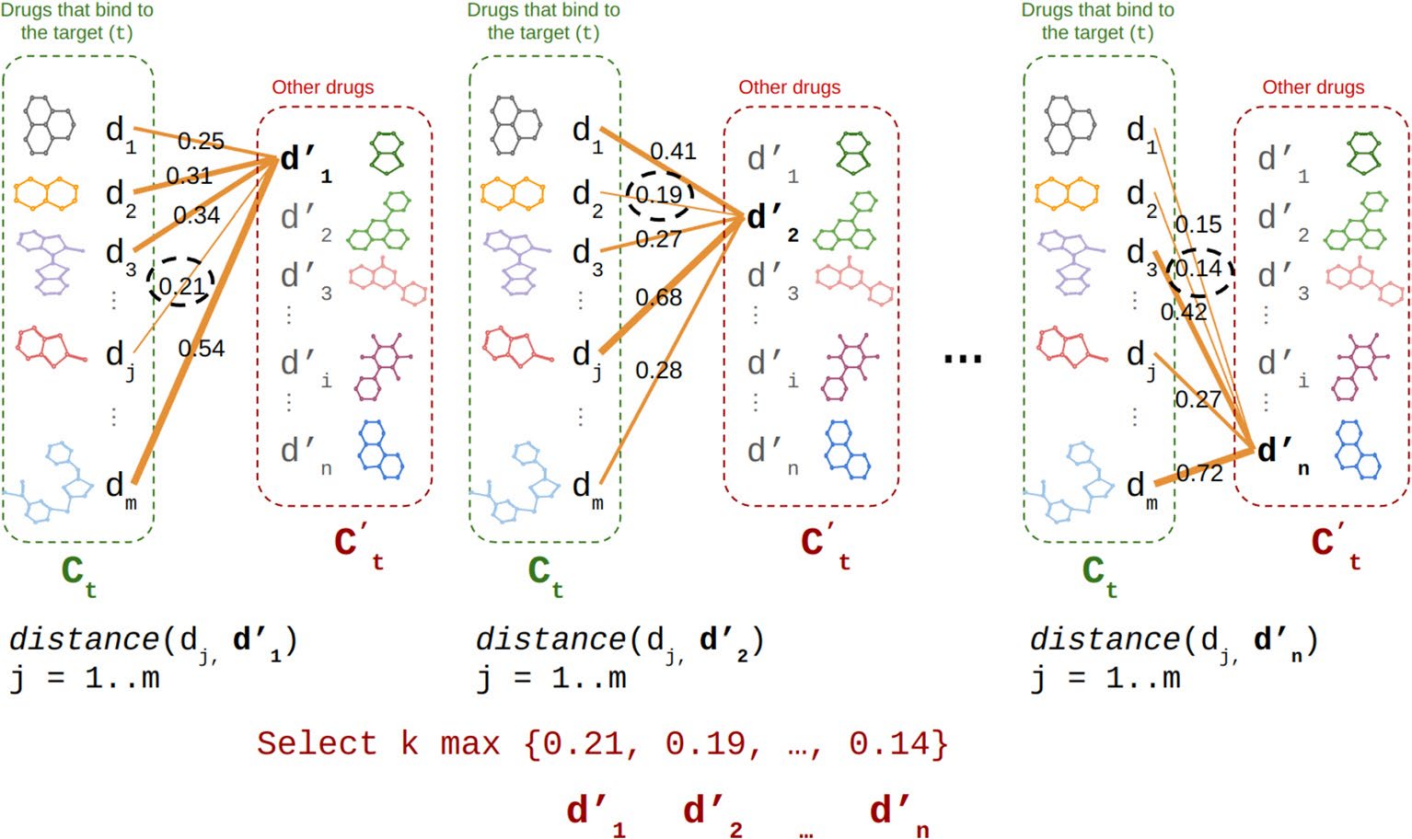
The process of generating negative samples for the DrugBank dataset

Let’s assume that a set of *m* compounds, denoted as $C_t:\left( d_1, d_2, \cdots , d_m \right)$, bind to a given target *t*, while the remaining compounds are included in another set, $C^\prime _t:\left( d^\prime _1, d^\prime _2, \cdots , d^\prime _n \right)$. For each compound $d^\prime _i$ in the $C^\prime _t$ set, we calculated its Tanimoto distance with all the compounds in the $C_t$ set. The minimum distance value obtained represents the distance of $d^\prime _i$ from the $C_t$ set. Ultimately, we select *k* compounds from the $C^\prime _t$ set that have the maximum distance to the $C_t$ set. Finally, we form pairs of target *t* with each of these *k* compounds to create a negative set. We then repeat the aforementioned steps for all targets in the DrugBank dataset (Fig. 13).

1 $$\begin{aligned} k \max _{i=1..n} \left( \min _{j=1..m} \left[ 1-Tanimoto \left( d_j,d^\prime _i \right) \right] \right) . \end{aligned}$$

#### Large-aggregated datasets

**Fig. 14:**
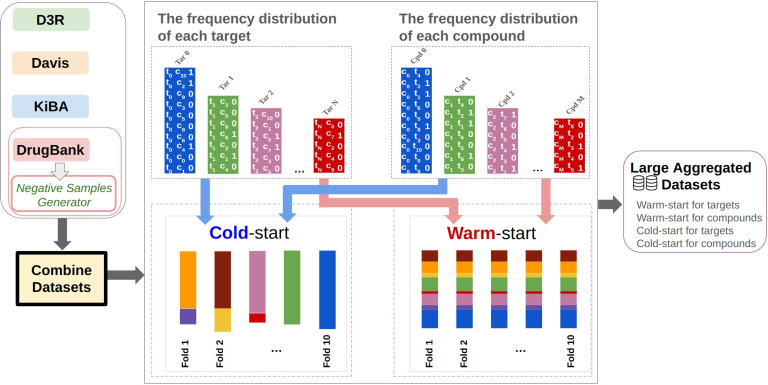
The process of cold-start and warm-start splitting for compounds and targets

The large aggregated dataset was created by combining the KiBA, DrugBank, Davis, and D3R datasets. We then applied warm-start splitting scenarios for targets and compounds to ensure consistent distribution between the training and test sets. Additionally, we employed cold-start splitting scenarios to challenge the models with new targets or compounds that they had not encountered in the training phase. Figure 14 illustrates the process of generating the cold-start and warm-start datasets for compounds and targets, with detailed information provided in Evaluation approaches section.

**Fig. 15:**
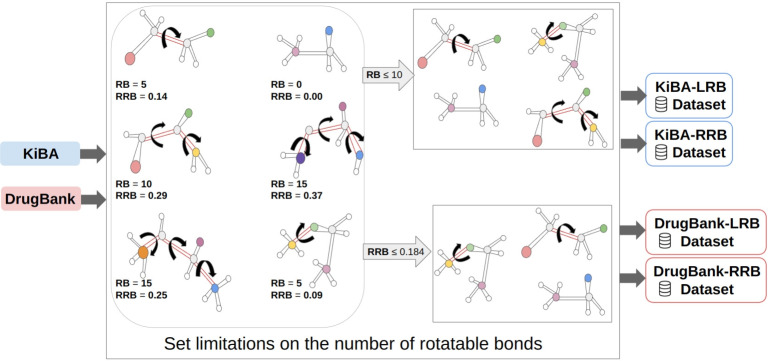
The process of generating the LRB and RRB datasets

#### Rotational bonds-aware datasets

A compound can possess zero or more rotatable bonds, which involve the overlapping of atomic orbitals. The rotation of these bonds is facilitated by the continuous rearrangement of electron density within the bonding region. Given that compounds with 10 or fewer rotatable bonds are highly likely to demonstrate favorable oral bioavailability in rats [27], we established a threshold by considering a maximum of 10 rotatable bonds to filter our datasets. Subsequently, we retained compounds that adhered to this criterion while excluding those that surpassed it. After applying this filtering process, our dataset is reduced to 282,971 records, forming what we refer to as the Limited-Rotatable-Bonds (LRB) dataset. The frequency distribution of compounds based on their number of rotatable bonds is presented in Supplementary Fig. S1. Notably, the majority of compounds within the dataset possessed between 0 and 10 rotatable bonds, with the highest recorded number of rotatable bonds reaching 95. It is plausible that compounds with 10 rotatable bonds out of a total of 19 may exhibit fewer interaction points and a more constrained conformational space compared to compounds with 10 rotatable bonds out of a total of 100 bonds. Based on the explanation mentioned earlier, in addition to the LRB dataset, we constructed the Ratio-based Rotatable Bonds (RRB) dataset. The RRB dataset is derived from the rotatable bond fraction (RBF) of the compounds. The RBF value is computed by dividing the number of rotatable bonds by the total number of bonds in a compound. To ensure a comparable dataset to the LRB dataset, we adjusted the number of records in the RRB dataset to match that of the LRB dataset. By setting the threshold of the RBF value to 0.184, we obtained a dataset consisting of 282,445 records (Supplementary Fig. S2). Figure 15 illustrates the process of creating LRB and RRB datasets from the DrugBank and KiBA datasets.

**Fig. 16:**
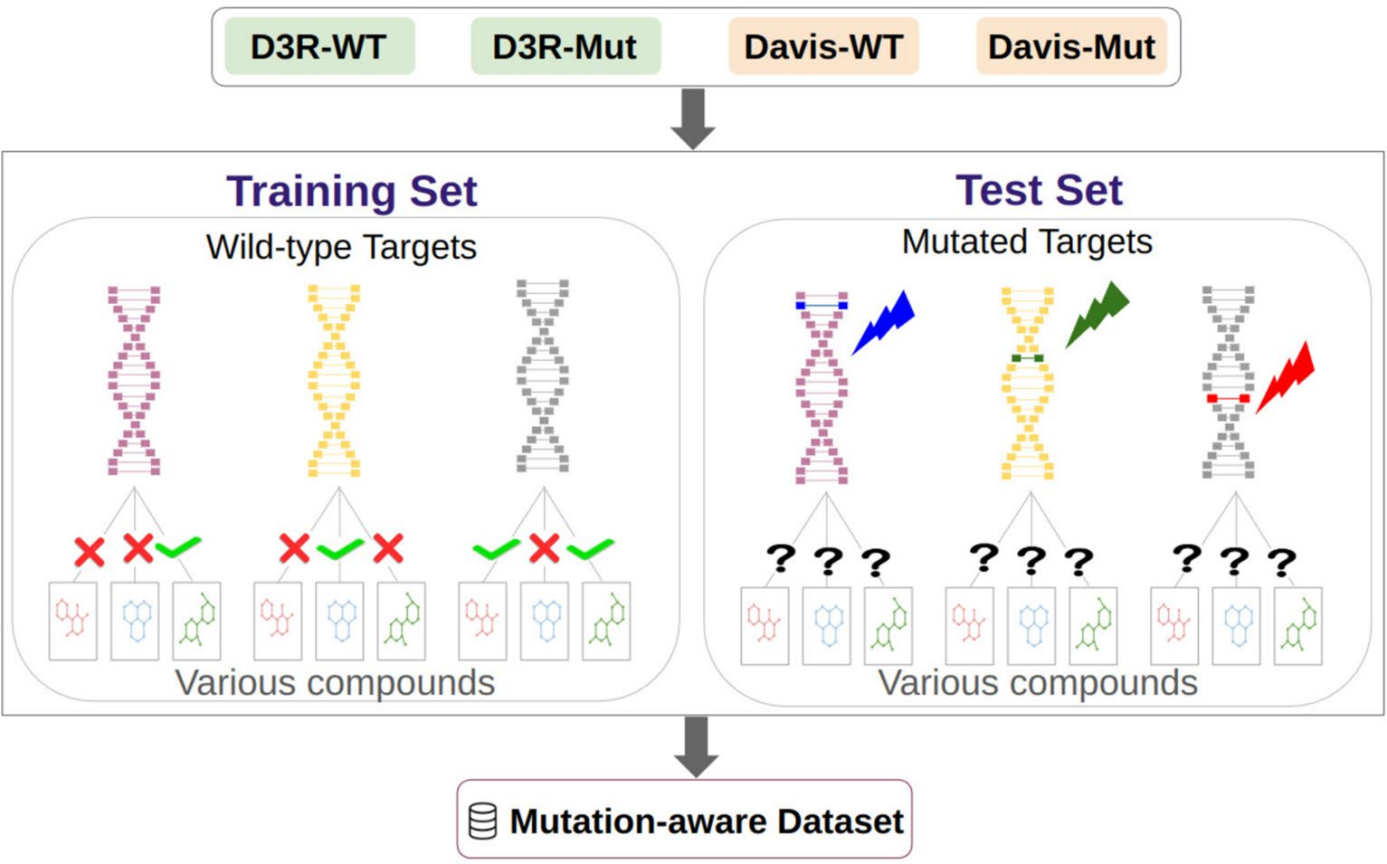
The mutation-aware dataset: the models are trained on interactions between compounds and wild-type targets, and evaluated on interactions between compounds and mutated targets

#### Mutation-aware dataset

One of the most predominant mechanisms underlying the development of treatment resistance in cancer cells and viruses is the emergence of mutations in the target protein [28]. Regardless of the type of mutation, a single point mutation in the amino acid sequence of a target protein can be sufficient to provoke drug resistance. Additionally, the relevance of these findings extends to genetic diseases, many of which lack effective therapies [29]. The discovery of new molecules that can restore the impaired protein function due to mutations is critically needed. Hence, accurate learning of the structure and functions of the wild-type proteins is of paramount importance to effectively measure and predict the binding status of compounds towards mutated versions of sequences. By employing this approach, we have appropriately configured the dataset to include training data with records of wild-type targets and test data with records of mutant targets (Fig. 16). We are interested in identifying models that can effectively capture informative features of targets and determining which target input features can serve as representative vectors for predicting compound-mutated target interactions.

**Fig. 17:**
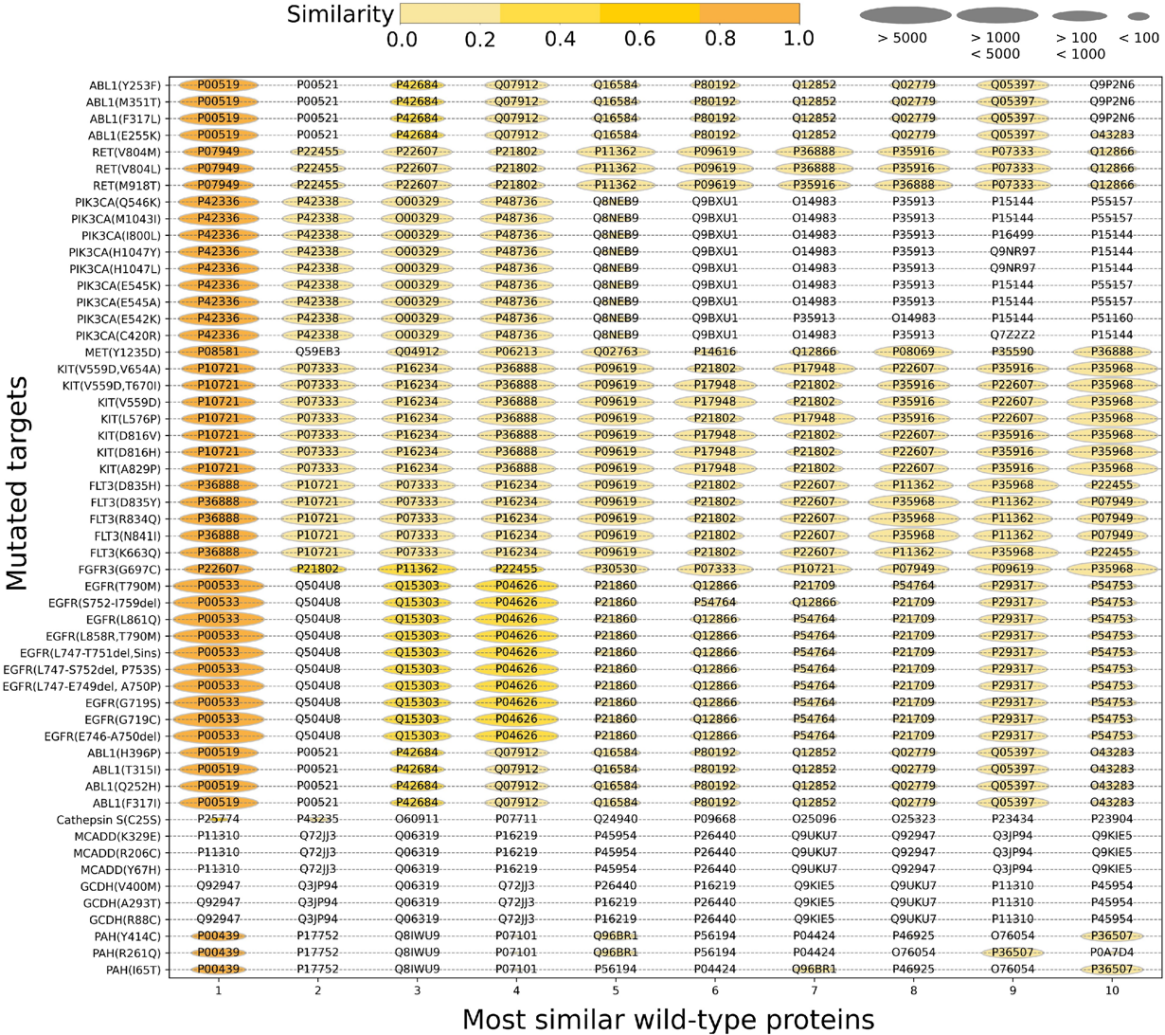
The mutation-aware dataset includes 54 mutated targets. Each row of the bubble plot represents a mutated protein, while the columns correspond to the 10 most similar wild-type proteins in the dataset, sorted in descending order of similarity. The size of the bubbles indicates the prevalence of the wild-type proteins in the training set, and the color intensity reflects the similarity score

To assess the similarity between the wild-type targets in the training set and the mutated targets in the test set, we employed a normalized alignment-based similarity score based on the Needleman-Wunsch algorithm. The bubble chart in Fig. 17 illustrates the 10 wild-type proteins most similar to 54 mutated targets in the mutation-aware dataset. Each row displays the UniProt ID of the wild-type proteins that are most similar to a specific mutated protein. The size of the ovals indicates the frequency of a wild-type protein’s occurrence in the training set, while the color of the ovals represents the degree of similarity between the wild-type and mutated proteins, with darker colors indicating higher similarity. The columns in the figure are sorted in descending order of similarity. As depicted, the first column of the bubble chart shows that for each mutated target in the test set, there is at least one wild-type target with a similarity score greater than 0.95. The training set contains a total of 65,611 compound-target records, representing 20% of the data, where the wild-type targets share more than 25% similarity with the mutated targets in the test set.

### Input features

#### Input features of compounds

**One-hot vectors**: The one-hot vector representation is a widely used method for encoding each character of the SMILES notation. Given that the SMILES notation consists of 98 unique characters, we construct a 98-dimensional vector for each character. Each dimension in the vector corresponds to a specific SMILES character, with a value of 1 indicating the presence of that character and 0 for all other dimensions. In our datasets, the maximum length of a SMILES sequence is 348 characters. To handle sequences of shorter lengths, we employ zero-padding techniques. As a result, we obtain a matrix of size (348, 98), which is then fed into convolutional neural network (CNN) layers and FC layers to produce a 128-dimensional representative vector for compounds.

**Trained embeddings**: Smi2Vec generates embeddings by first encoding each symbol in the SMILES notation as a one-hot representation, and then training a linear classifier to predict the original symbol, similar to the Word2Vec approach. One key advantage of Smi2Vec is its broader perspective - it doesn’t just treat sequences as language sentences, but also treats other symbols like ’ @ ’, ’[’, ’]’, and others as “separate biological terms”. This unique approach enables Smi2Vec to create meaningful embeddings even for special cases like chirality. Therefore, the Smi2Vec [30] technique captures the specific characteristics of each letter in a SMILES notation, generating a matrix for a given sequence. The rows of this resulting matrix then serve as a lookup table for embeddings. The IIFDTI model utilizes the SMILES notation of a compound to generate a matrix of 100-dimensional Smi2Vec embeddings, which serves as one of the representations for compounds.

**2D drug fingerprints (2DFP)**: We employed the concatenation of four widely used 2D drug fingerprints, namely Morgan, MACCS, RDKit-2D, and AtomPair fingerprints, with lengths of 1024, 167, 1024, and 1024, respectively. These fingerprints capture various aspects of the compounds, including atom pair information, encoded circular radius-2 substructure information, and global pharmacophore information. Consequently, our compound input features comprise a total of 3239 dimensions. To extract informative features, the 2DFP-based features underwent FC layers, resulting in a 128-dimensional vector as a representative compound vector. The DeepConv-DTI model also employs a Morgan drug fingerprint vector with a dimensionality of 2048.

**3D drug fingerprints (E3FP)**: E3FP [31] captures the 3D conformers of compounds by defining spheres of varying radii. These spheres not only consider bond types and connectivity details but also incorporate the 3D positions of atoms. The process begins with small spheres containing a single atom and gradually expands them to include neighboring positions and connectivity details. This iterative process continues until the sphere encompasses the entire compound. Consequently, the E3FP drug fingerprint generates multiple binary vectors that describe the structure and atom descriptors of various conformers. Dimensionality reduction can be applied to the 3D drug fingerprints using bitwise operators if desired. In this study, we generated a 2048-dimensional vector for each conformer. We utilized the default configuration of the E3FP model, which allows a maximum of 3 conformers per compound. This choice is recommended in the literature for improved accuracy. Therefore, for each compound, we obtained a matrix of size (3, 2048), which was subsequently fed into a CNN layer and a linear layer to obtain a 128-dimensional representative compound vector.

**2D atoms interaction network**: The 2D atoms interaction network was constructed using the RDKit tool, which allowed us to extract the adjacency matrix representing the graph of atom interactions for each compound. To utilize graph-based neural networks such as GCN and GAT layers, it is necessary to assign an initial embedding vector to each node (atom) in the graph. These initial embeddings can be represented as one-hot vectors or atom characteristic vectors. For this study, we utilized 34-dimensional vectors as initial embeddings for each atom in the interaction graph. These dimensions capture various features of the atoms, including (i) atom type (*C*, *N*, *O*, *F*, *P*, *S*, *Cl*, *Br*, *I*,  other), represented as a one-hot vector with 10 dimensions, (ii) atom degree, which indicates the number of carbons attached to the atom (ranging from 0 to 6), represented as a 7-dimensional one-hot vector, (iii) Formal charge represents the electric charge associated with an atom and is determined by considering the number of shared and non-bonding valence electrons. It is represented as a binary value, indicating the presence or absence of an electronic charge, (iv) hybridization type (*SP*, *SP*2, *SP*3, *SP*3*D*, *SP*3*D*2,  other), which describes the molecular geometry, angles, etc., represented as a 6-dimensional one-hot vector, (v) chirality, with a value of 1 indicating a possible chiral center and 0 otherwise, (vi) radical atoms, with a value of 1 indicating the presence of at least one unpaired electron and 0 otherwise, (vii) aromatic, with a value of 1 indicating membership in an aromatic ring and 0 otherwise, (viii) absolute configuration (*R* or *S*), describing the spatial arrangement of atoms, represented as a two-dimensional one-hot vector, and (ix) the number of hydrogen atoms attached (ranging from 0 to 4), represented as a 5-dimensional one-hot vector. By concatenating all the one-hot vectors and binary values, we obtained a final 34-dimensional vector representation for each atom in the interaction graph. The TransformerCPI model utilizes a GCN and we utilize Graph Attention v2 (GATv2) [32] and FC layers to extract a 128-dimensional compound representative vector in the AlphaFoldGrAtts and PhyGrAtt models.

#### Input features of targets

**One-hot vectors**: Since we have 20 unique amino acids, we construct a 20-dimensional vector for each amino acid. Each dimension in the vector corresponds to a specific amino acid, with a value of 1 indicating the presence of that amino acid and 0 for all other dimensions. In our datasets, the maximum number of amino acids in a target sequence is 1400. To handle sequences of shorter lengths, we employ zero-padding techniques. As a result, we obtain a matrix of size (1400, 20), which is then fed into convolutional and FC layers to produce a 128-dimensional representative vector for the target.

**Physicochemical properties**: The physicochemical properties of amino acids are represented as 512-dimensional vectors obtained from AAindex [33]. Prior studies have demonstrated that incorporating AAindex features as physicochemical properties of amino acids can effectively predict cancer subtypes [18] and protein-protein interactions [34] with promising performances. To reduce the dimensionality, we employed Principal Component Analysis (PCA), reducing it to 20 dimensions. Given that the maximum number of amino acids for target sequences is 1400, we obtained a (1400, 20) matrix as the target input feature. These features were then fed into convolutional and FC layers to further reduce the dimensionality to a 128-dimensional vector, serving as the representative target vector.

**K-mers representation**: One of the most popular methods for analyzing sequence-based input features is extracting K-mers using a sliding window approach over the amino acid sequence. Once the K-mers are extracted, a language model such as Skip-gram can be employed to obtain Word2Vec embeddings for the K-mers. To obtain the representative features for the target, TransformerCPI utilizes a positional transformer-encoder block.

**Trained embeddings**: Prot2Vec [35] generates embedding vectors through the training of a Skip-gram neural network on non-overlapping sequences of 3-mers within each protein. The obtained embeddings for each 3-mer subsequently serve as an embedding lookup table. These embeddings have proven effective in the classification of protein families. A protein family consists of proteins sharing similar functions or structures, often linked through evolutionary relationships. Within the IIFDTI model, the amino acid sequence is employed to construct 100-dimensional Prot2Vec embeddings. This matrix acts as one of the representations for proteins within the encoder-decoder block.

**UniRep-based features**: The UniRep-based input features are derived from a multiplicative long short-term memory (mLSTM) model [36], which has been trained on a vast dataset comprising approximately 25 million amino acid sequences from the UniRef50 clusters of UniProt. The primary objective of the mLSTM model is to predict the next amino acid in a sequence based on the preceding context. Alley et al. conducted an evaluation of their model on various tasks, including protein secondary structure, evolutionary information, semantic similarity, functional annotations, and stability predictions. In this study, a UniRep-based embedding with 1900 dimensions is processed through a series of FC layers to extract a 128-dimensional representative vector for the targets.

**BERT-based features**: The BERT-based input features are obtained from a semi-supervised learning model called TAPE [37], which has been specifically designed and evaluated for predicting a wide range of tasks in protein analysis. These tasks include protein secondary structure prediction, contact prediction, remote homology detection, stability estimation, and fluorescence landscape prediction. The TAPE model has been trained on the extensive Pfam database, which comprises 31 million protein domains. The Pfam database organizes amino acid sequences into families based on their evolutionary relationships. For each input amino acid sequence, the TAPE model generates a 768-dimensional embedding, which is then processed through a series of FC layers to obtain a 128-dimensional representative vector for the target.

**3D structures**: The 3D structures of targets can be extracted using the AlphaFold model [38]. Recent research has demonstrated that the 3D structures of proteins obtained from AlphaFold provide informative features for various aspects of protein functionality and stability [39–41]. One of the main advantages of AlphaFold is its ability to predict the 3D structure of proteins for which the structure is unknown. However, this is not the only advantage. AlphaFold can also be used for proteins with available 3D structures in the Protein Data Bank (PDB). The PDB files can occasionally exhibit missing amino acids. There are multiple reasons why a PDB file may contain missing amino acids.

In some cases, certain regions of proteins may not be resolved with sufficient clarity using X-ray crystallography or cryo-electron microscopy techniques. Additionally, incomplete sequencing can lead to missing amino acids. Therefore, AlphaFold predictions can help in predicting the structure of these missing amino acids. To illustrate this benefit of the AlphaFold model, we compared the recorded 3D structure of the Phenylalanine-4-hydroxylase (PH4H) protein (RCSB PDB ID: 6HYC) with its predicted 3D structure obtained from AlphaFold. Supplementary Fig. S19-a shows the amino acid sequence of the protein, with the red bold characters representing the missing amino acids in the 6HYC PDB file. As shown in supplementary Fig. S19-b, the predicted and actual 3D structures of the protein overlap, demonstrating the accuracy of the AlphaFold prediction. The blue part of the figure represents the AlphaFold prediction, the brown part represents the actual structure, and the gray parts represent the predicted structures of the missing amino acids. The high accuracy of the AlphaFold model in predicting protein 3D structures has motivated us to explore its utility in the field of CTI prediction. Based on the 3D structure of proteins obtained from AlphaFold, we extracted the coordinates of atoms and amino acids. Our focus in this case was on identifying the chemical contacts between the amino acids. To achieve this, we utilized the Residue Interaction Network Generator (RING 3.0) tool [42], which is capable of generating a weighted graph of residue interactions based on the angstrom distances between atoms in three-dimensional space. Supplementary Fig. S19-c displays the residue interaction network of the PH4H protein, which has been generated using RING. The edges of the graph are assigned weights that reflect the strength of the interactions. RING identifies and assigns dissociation energies to various types of bonds, including disulfide bridges, ionic bonds, hydrogen bonds, $\pi$-Cation, $\pi$-$\pi$ stack, and Van der Waals interactions. The dissociation energies assigned are 167, 20, 17, 9.6, 9.4, and 6, respectively. The different bond types have varying dissociation energies, hence the weighted graph representation. We utilized a two-layer GATv2 model to learn the weighted graph features of a target. The learned features were then concatenated and passed through FC layers to obtain a 128-dimensional representative for the target in the AlphaFoldGrAtts model.

### Models

In this study, our objective is to conduct a comprehensive comparison of different models based on structure (or sequence) for CTI prediction. Given our focus on the efficacy of protein and compound input features, we have excluded relational-based models that rely on diverse data sources such as compound-compound similarity networks and protein-protein interactions. These models do not align with the primary objective of our study.

There exist several state-of-the-art models for predicting CTIs, including DeepDTA, DeepCAT, TransformerCPI, IIFDTI, and DeepConv-DTI. These models employ varied input features for compounds and targets, in addition to utilizing distinct types of neural network blocks. DeepDTA [7] employs a character-level embedding layer to generate unique embeddings for each character in the SMILES notation. Subsequently, these embeddings are passed through three convolutional layers, mirroring a similar process for proteins. Ultimately, the model processes these captured representations through FC layers (Supplementary Fig. S20). DeepCAT [18] represents another model designed specifically for predicting cancer subtypes. In this research, we leverage the identical input features involving amino acid sequences and the CNN block of DeepCAT to handle protein representation. However, for the compound component, we adopt a similar approach, utilizing one-hot vector representation for the characters within the SMILES notation (Supplementary Fig. S21). TransformerCPI [6] represents a model that extracts the atom interaction network from compounds, acquiring atom input features through GCN. Additionally, it captures target input features by extracting k-mers and employing the Word2Vec technique. Ultimately, it deploys encoder and decoder blocks within transformers to effectively capture the interwoven features of compounds and proteins (Supplementary Fig. S22). IIFDTI [5] constructs a molecular graph from the SMILES string, anchoring initial feature vectors, which encompass physicochemical properties, to each atom within the graph. This model comprises two distinct feature extraction blocks and a consolidated bidirectional encoder-decoder block. The autonomous feature extractors, manifested as a multi-layer GAT network and a CNN, take the molecular graph and the protein sequence’s Word2Vec-embedded 3-mers, respectively. The bidirectional encoder-decoder block follows an architecture similar to the original Transformer proposed by Vaswani et al. [43]. However, in this scenario, each block encompasses a one-dimensional convolutional layer and a gated linear unit. The input features encompass SMILES notations, enriched through a pre-trained Smi2Vec model [30], alongside the sequence of overlapping 3-mers for the amino acid sequence, bolstered by a pre-trained Prot2Vec model [35] (Supplementary Fig. S23). DeepConv-DTI [8] employs the amino acid sequence and the binary Morgan drug fingerprint with a radius of 2 as its input features. This model integrates two distinct feature extraction blocks: one for compounds and another for targets. The outcomes of these blocks are joined together, subsequently traversing final FC layers to predict interactions. The target’s feature extractor adopts a trainable embedding for every amino acid in the protein sequence, followed by global one-dimensional convolutional layers. These global convolutional layers capture enlightening features using windows of assorted scales (Supplementary Fig. S24).

In addition to the five aforementioned state-of-the-art models, we devised seven additional models, each grounded on distinct input features. For each of these models, we selected the most fitting neural network components. To determine the optimal hyperparameters for the models, we employed a subset comprising $10\%$ of the entire dataset as the validation set. In the subsequent subsections, we will elucidate each of these models individually. For each layer of the models, dropout and batch normalization techniques are employed to mitigate issues such as exploding gradients and overfitting. Table 1 and supplementary Fig. S20-S29 provide a comprehensive overview of the model’s architecture.

#### Random Forest

We generated one-hot vectors to represent the atoms of compounds. Simultaneously, for the protein side, we depicted each amino acid using its corresponding physicochemical properties. Afterward, the vectors are concatenated. Lastly, we applied PCA to extract 32 principal components from the merged vectors. These resultant vectors were then fed into a random forest classifier.

#### AlphaFoldGrAtts

The AlphaFoldGrAtts model operates by taking the 2D atoms interaction network of compounds and assigning a 34-dimensional vector to each atom (as described in "Input features of compounds" subsection). Simultaneously, it extracts the weighted residue interaction graph utilizing AlphaFold and RING methods and subsequently assigns 20-dimensional vectors to each amino acid. These vectors represent the amino acid’s physicochemical properties derived from PCA (as described in "Input features of targets" subsection).

A graph $G = \left( V, E, F \right)$ consists of nodes $V = \{ 1, \cdots , n \},$ edges $E \subseteq V \times V,$ where $\left( i, j \right) \in E$ denotes an edge from a node *i* to a node *j*, and features $F = \{ f_1, \cdots , f_n \},$ where $f_i \in {\mathbb {R}}^{34}$ ($f_i \in {\mathbb {R}}^{20}$) is the initial features vector of atom (amino acid) *i*. The significance of the features of the neighbor node *j* with respect to node *i* is defined by a scoring function:

2 $$\begin{aligned} e(f_i,f_j)=a^T LeakyRELU(W.\left[ f_i \Vert f_j \right] ), \end{aligned}$$

where *a* and *W* are trainable matrices and $\parallel$ denotes concatenation. Then, the attention function will be calculated based on the normalization across all neighbors $k \in N_i$ using the softmax function:

3 $$\begin{aligned} \alpha _{ij}=softmax(e(f_i,f_j))=\frac{e(f_i,f_j)}{\sum _{k \in N_i} exp(e(f_i,f_k))}. \end{aligned}$$

Then, every node updates its feature vector by receiving messages from its neighboring nodes.

4 $$\begin{aligned} f^\prime _i = \sigma \left( \sum _{j \in N_i} \alpha _{ij}.Wf_j\right) . \end{aligned}$$

The phase of updating the feature vectors involves not only updating the vector members but also adjusting the vector dimensions. The entire process of updating the feature vectors, as described in Eq. 2-4, is referred to as the GATv2 model. GATv2 represents a novel graph attention mechanism that addresses the limitation of static attention and extends the attention mechanism to align with the dynamic context. Hence, the feature vectors will be updated using GATv2.

5 $$\begin{aligned} \left( V, E, F^\prime \right) = GATv2 \left( V, E, F \right) , \end{aligned}$$

where $F^\prime = \{ f^\prime _1, \cdots , f^\prime _n \}$ is the updated feature vectors, $f^\prime _i \in {\mathbb {R}}^{8},$ and $n=1400$ denotes the maximum number of amino acids in a protein sequence. To further update the features based on the most recent version of the updated feature vectors, we employ an additional layer of GATv2.

6 $$\begin{aligned} \left( V, E, F^{\prime \prime } \right) = GATv2 \left( V, E, F^\prime \right) , \end{aligned}$$

where $F^{\prime \prime } = \{ f^{\prime \prime }_1, \cdots , f^{\prime \prime }_n \}$ and $f^{\prime \prime }_i \in {\mathbb {R}}^{4}$. Subsequently, the updated feature vectors are concatenated to form a 5600-dimensional vector. This vector is then fed through multiple FC layers to generate a 128-dimensional target representative vector. Similarly, for the compound portion, AlphaFoldGrAtts applies a similar process, utilizing two GATv2 layers and several FC layers to extract a compound representative vector. Ultimately, these two representative vectors are concatenated and further processed through additional FC layers. The output layer comprises two neurons that represent the probability of the compound-target interaction. Subsequently, a cross-entropy loss function is defined.

7 $$\begin{aligned} {\mathcal {L}} \left( y^\prime ,y \right) = - \left[ y \log y^\prime + \left( 1 - y \right) \log \left( 1 - y^\prime \right) \right] , \end{aligned}$$

where *y* and $y^\prime$ represent the actual and predicted output values, respectively. The AlphaFoldGrAtts model minimizes this loss function using a stochastic gradient descent (SGD) optimizer.

#### PhyGrAtt

Similar to the AlphaFoldGrAtts model, PhyGrAtt employs a process to extract 34-dimensional feature vectors for compounds. It accomplishes this by utilizing two GATv2 layers (as described in Eq. 5 and 6) and multiple fully connected layers to capture the representative vectors. However, in contrast to the protein side that uses GATv2 layers, the PhyGrAtt model attributes physicochemical properties ($f_i \in {\mathbb {R}}^{20}$) to each amino acid. These features are then directed through a 2D convolutional layer followed by a max pooling layer for further processing.

8 $$\begin{aligned} F^\prime =MaxPool2D \left( Conv2D \left( F,k_{c_1}, p\right) , k_{{MP}_1},s \right) , \end{aligned}$$

where $F = \{ f_1, \cdots , f_n \}$ denotes the input features, $p=1$ and $s=1$ denote the amount of padding and stride, and $K_{c_1}=(603,8)$ and $k_{{MP}_1}=(201,4)$ are the kernel size of the convolutional and the max pool layers, respectively. To further reduce the dimensionality of the feature matrix ($F^\prime \in {\mathbb {R}}^{600 \times 12}$), we incorporated additional 2D convolutional and max pooling layers with kernel sizes of $K_{c_2}=(303,7)$ and $k_{{MP}_2}=(101,4)$, respectively.

9 $$\begin{aligned} F^{\prime \prime }=MaxPool2D \left( Conv2D \left( F^\prime ,k_{c_2}, p\right) , k_{{MP}_2},s \right) . \end{aligned}$$

Passing the $F^\prime$ features through the above 2D convolutional and max pooling layers yields a matrix $F^{\prime \prime } \in {\mathbb {R}}^{200 \times 5}$. This matrix is subsequently flattened and transformed into a 1000-dimensional vector, which is then propagated through several FC layers to derive the representative features of the target. Lastly, the representative vectors of targets and compounds are merged and inputted into multiple FC layers. PhyGrAtt formulates a cross-entropy loss function and aims to minimize it using an SGD optimizer.

#### The E3FP-based model

The process of extracting the target representative vector closely resembles that of the PhyGrAtt model. It employs two sets of 2D-CNN blocks coupled with max pooling layers. Additionally, as elaborated in "Input features of compounds" subsection, the E3FP-based model extracts drug fingerprints from various potential conformers. These drug fingerprints are then subjected to a 2D CNN and max pooling layers.

10 $$\begin{aligned} F^\prime =MaxPool2D \left( Conv2D \left( F,k_{c}, p\right) , k_{MP},s \right) , \end{aligned}$$

where $F \in {\mathbb {R}}^{3 \times 2048}$ is the 3D drug fingerprints matrix, $p=1$ and $s=1$ denote the amount of padding and stride, and $K_c=(3, 501)$ and $K_{MP}=(3, 5)$ are the kernel size of the convolutional and the max pool layers, respectively. Subsequently, the output of the max pooling layer, denoted as $F^\prime \in {\mathbb {R}}^{310}$, is inputted into an FC layer to extract the 128-dimensional compound vector. Ultimately, the representative vectors of both targets and compounds are concatenated and passed through multiple FC layers. The model employs an SGD optimizer to minimize a cross-entropy loss function.

#### The 2DFP-based model

This model accepts a 3239-dimensional vector composed of four different drug fingerprints: Morgan, MACCS, RDKit-2D, and AtomPair. The vector is then passed through three FC layers to obtain the compound representative vector ($F^{\prime \prime \prime } \in {\mathbb {R}}^{128}$).

11 $$\begin{aligned} \begin{aligned} F^\prime = RELU \left( W_{c_1}F+b_{c_1} \right) , W_{c_1} \in {\mathbb {R}}^{2048 \times 3239}, b_{c_1} \in {\mathbb {R}}^{2048}, \\ F^{\prime \prime } = RELU \left( W_{c_2}F^\prime +b_{c_2} \right) , W_{c_2} \in {\mathbb {R}}^{512 \times 2048}, b_{c_2} \in {\mathbb {R}}^{512}, \\ F^{\prime \prime \prime } = W_{c_3}F^{\prime \prime }+b_{c_3}, W_{c_3} \in {\mathbb {R}}^{128 \times 512}, b_{c_3} \in {\mathbb {R}}^{128}, \end{aligned} \end{aligned}$$

where $W_{c_i}$ and $b_{c_i}$ are the weight matrices and biases, respectively. Similar to PhyGrAtt and the E3FP-based models, the 2DFP-based model utilizes the physicochemical properties of amino acids as input features and feeds them into convolutional and max pooling layers to derive a 128-dimensional target representative vector. The combined vector resulting from the concatenation of target and compound representative vectors is then passed through FC layers. The weight matrices and trainable parameters are updated using an SGD optimizer to minimize a cross-entropy loss function.

#### The BERT (and UniRep)-based models

The compound side of both models, similar to the 2DFP-based model, relies on a 3239-dimensional vector obtained by concatenating four distinct drug fingerprints. The process of obtaining a 128-dimensional compound representative vector is identical to that of the 2DFP-based model. Regarding the protein side, we employed a transfer learning-based approach named TAPE to extract features from amino acid sequences. As implied by their names, the UniRep-based model employs a language model, UniRep, trained on 25 million sequences, while the BERT-based model employs a language model, BERT, trained on 31 million sequences. The TAPE tool yields 1900- and 768-dimensional embeddings for the UniRep-based and BERT-based models, respectively. These embeddings are subsequently passed through a series of FC layers. Similar to the other models, we employed the SGD optimization algorithm to minimize the cross-entropy loss function.

#### PhyChemDG

This model is an adapted version of TransformerCPI. In this instance, we substituted the Word2Vec-based embeddings of the extracted k-mers with 20-dimensional physicochemical properties of amino acids. As a result, all other configurations, such as the GCN block for compounds and the encoder-decoders of the transformer, remain consistent with the TransformerCPI model.

### Evaluation approaches

**Warm-start for compounds**: Considering the high cost of drug development and the need for increased safety, it is valuable to explore the utilization of existing compounds that have already received FDA approval for new diseases [1]. This approach, known as drug repurposing, allows for the repurposing of existing compounds for different targets. In such cases, the training and test sets consist of shared compounds. To maintain consistent compound distribution between the training and test sets, we partitioned the compound-target records into 10 equal-sized groups (Fig. 14). During each iteration, one group is utilized as the test set, while the remaining groups are concatenated to form the training set. This systematic approach guarantees that the distribution of compounds remains unaltered between the training and test sets.

**Warm-start for targets**: In this experimental setting, the distribution of targets in the test set aligns with that of the training set. Consequently, the models are exposed to target features that have already been encountered during the training phase. Hence, the models become familiar with the structure and characteristics of the targets during the prediction phase.

**Cold-start for compounds**: Finding models capable of identifying potential targets that may interact with newly developed compounds is of paramount importance. In the cold-start scenario for compounds, the dataset is divided into training and test sets, with the test set containing compounds that did not appear during the training phase. To achieve this, we initially sorted the dataset in descending order based on the number of records for each compound. We then create 10 empty groups and begin assigning the compound-target records to the groups. We start by assigning the compound-target records of the most frequent compound to the first group, followed by the second most frequent compound to the second group, and so on until we reach the 10th group. Once the compound-target records of the 10th most frequent compound are assigned to the 10th group, we start the assignment process again from the first group (Fig. 14). Since the total dataset size is 337,526, we set a threshold of 34,000 for each group. Therefore, we fill each group until the threshold of 34,000 records is reached.

**Cold-start for targets**: The technique employed for the cold-start for targets scenario is similar to the cold-start for compounds. However, in this case, we utilize records of targets instead of compounds. The goal is to determine which models are capable of capturing target features effectively, enabling accurate prediction of compound-target interactions for unseen targets. The concept of employing diverse splitting scenarios has been applied in various studies, including T-cell receptor specificity [44] and knowledge graph-based CTI [16] prediction models. In cold-start splitting scenarios, it’s important to consider the frequency of targets and compounds in the dataset. We utilized 10-fold cross-validation to evaluate the models, and with over 300,000 compound-target records, each fold’s size needed to be approximately 30,000 to maintain equal fold sizes. Supplementary Fig. S30 displays histograms depicting the frequency of different compounds and targets. As shown in the figure, the highest occurrence of a target and a compound in the large aggregated dataset is 6,174 and 3,933, respectively.

## Supplementary Information

**Additional file 1.** Supplementary information.

## Data Availability

All data are freely available at https://imsb.s3.baiome.org/pub/Datasets.zip.

## Code availability

The source code of this study is available at https://github.com/sabdollahi/CTI_Comparisons.
