# Supplementary material for: A comprehensive comparison of deep learning-based compound-target interaction prediction models to unveil guiding design principles
Source: J Cheminform. 2024 Oct 28;16:118. doi: 10.1186/s13321-024-00913-1 (PMC11520803; doi:10.1186/s13321-024-00913-1)
Supplement: Supplementary file 1 — Additional file 1. Supplementary information. [file 13321_2024_913_MOESM1_ESM.pdf]

Supplementary Figures and Tables: A comprehensive comparison of deep learning-based compound-target interaction prediction models to unveil guiding design principles

Sina Abdollahi[1], Darius P. Schaub[1,2], Madalena Barroso[3†], Nora C. Laubach[3†], Wiebke Hutwelker[3†], Ulf Panzer[2,4], Søren W. Gersting[3*], Stefan Bonn[1,4,5*]

[1] Institute of Medical Systems Biology, University Medical Center Hamburg-Eppendorf, Hamburg, 20251, Germany.
[2] III. Department of Medicine, University Medical Center Hamburg-Eppendorf, Hamburg, 20251, Germany.
[3]University Children's Research, UCR@Kinder-UKE, University Medical Center Hamburg-Eppendorf, Hamburg, 20251, Germany.
[4]Hamburg Center for Translational Immunology (HCTI), University Medical Center Hamburg-Eppendorf, Hamburg, 20251, Germany.
[5]Center for Biomedical AI, University Medical Center Hamburg-Eppendorf, Hamburg, 20251, Germany.

*Corresponding author(s). E-mail(s): gersting@uke.de; stefan.bonn@zmnh.uni-hamburg.de;
†These authors contributed equally to this work.

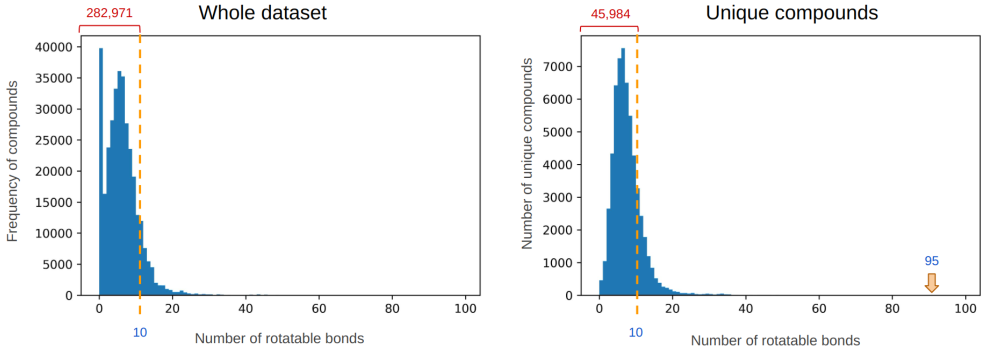

Supplementary Figure 1: The frequency distribution of compounds based on their number of rotatable bonds. The number of rotatable bonds in the compounds within the large aggregated dataset ranges from 0 to 95. The Limited-Rotatable-Bonds (LRB) dataset is created by retaining compounds with fewer than 10 rotatable bonds. As a result, 282,971 compound-target samples out of 337,526 meet this criterion. The LRB dataset includes 45,984 unique compounds, representing approximately 79% of the compounds in the main dataset. Additionally, there are 460 compounds without any rotatable bonds, which are associated with 39,796 compound-target interaction samples in the main dataset. There are approximately 400 compounds with zero rotational bonds. The entire dataset comprises over 37,000 positive and negative CTIs involving these compounds. On average, each compound with zero rotational bonds appears 92 times across the dataset.

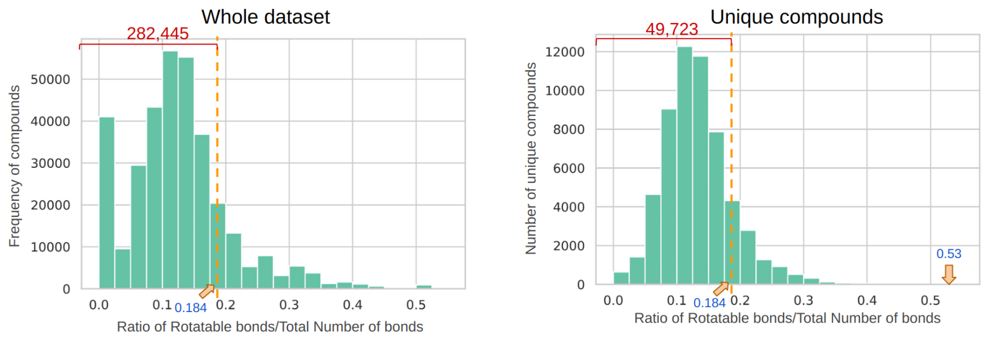

Supplementary Figure 2: The frequency distribution of compounds based on the ratio of the number of rotatable bonds over the total number of bonds. The ratio is referred to as the Rotatable Bond Fraction (RBF), and the Ratio-based Rotatable Bonds (RRB) dataset is created by retaining compounds with an RBF of less than 0.184. The RRB dataset includes 49,723 unique compounds, representing approximately 85% of the compounds in the large aggregated dataset.

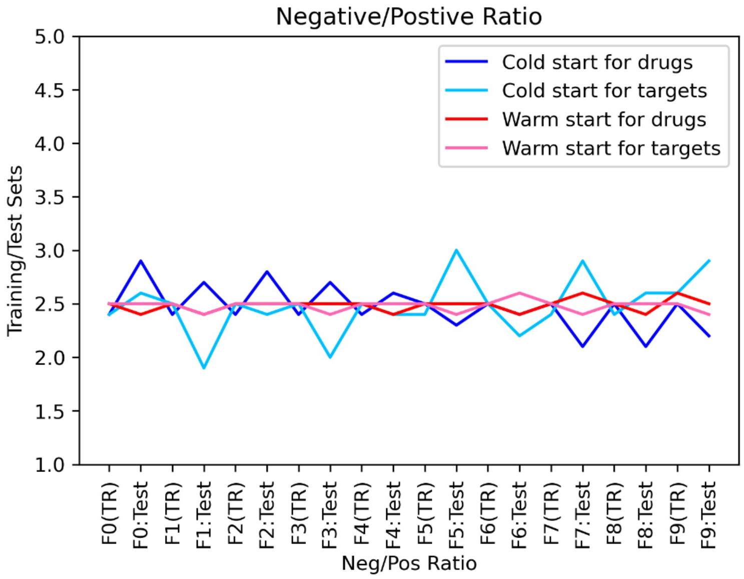

Supplementary Figure 3: The ratio of negative samples to positive samples varies across different folds. 'F0(TR)' represents the combination of all folds except fold 0, while 'F0: Test' represents fold 0 itself.

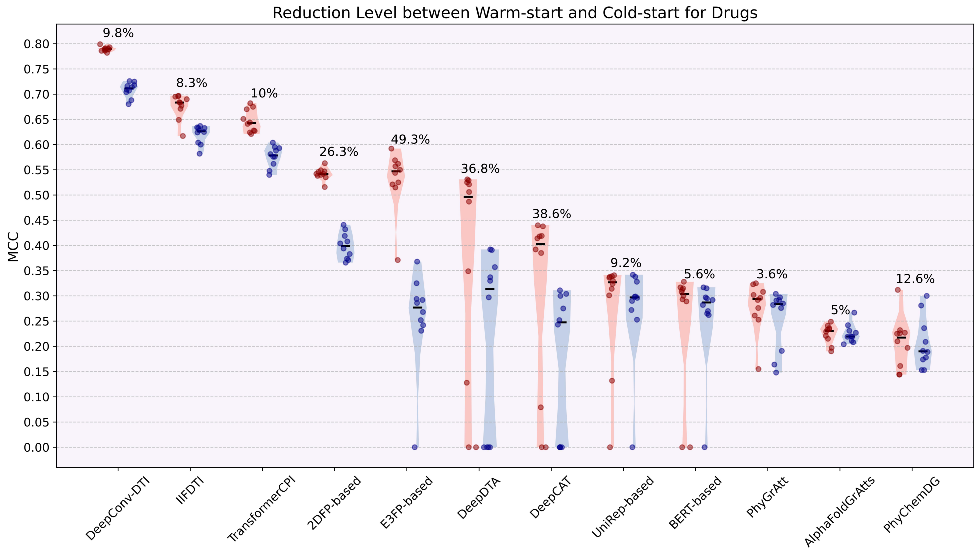

Supplementary Figure 4: The reduction disparity between warm-start and cold-start for compounds. The reduction level in performance for compound-based models, such as 2DF-based and E3FP-based models, is quite significant due to their reliance on the compounds' 2D or 3D structures.

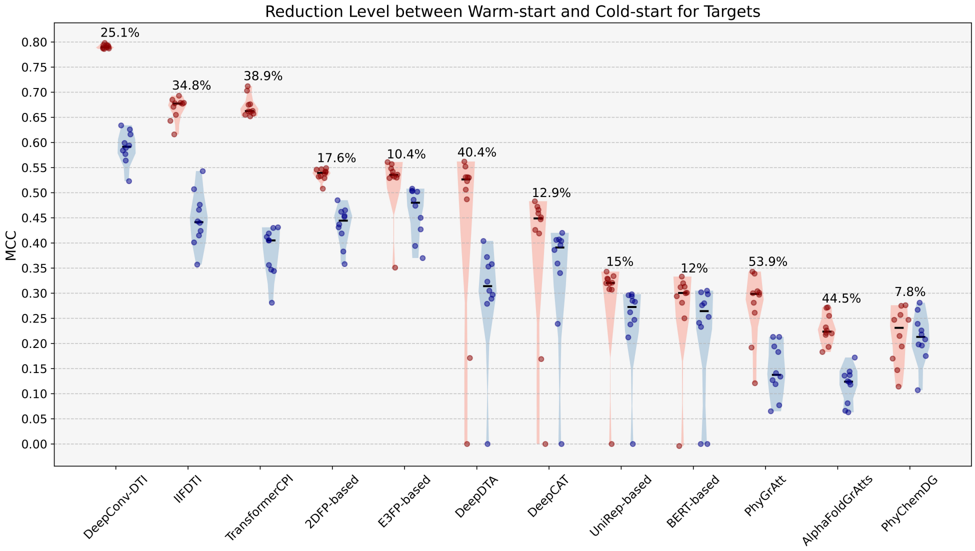

Supplementary Figure 5: The reduction disparity between warm-start and cold-start for targets. The reduction level in performance for protein structure-based models, such as AlphaFoldGrAtts and PhyGrAtt, is quite significant due to their reliance on the proteins' structures.

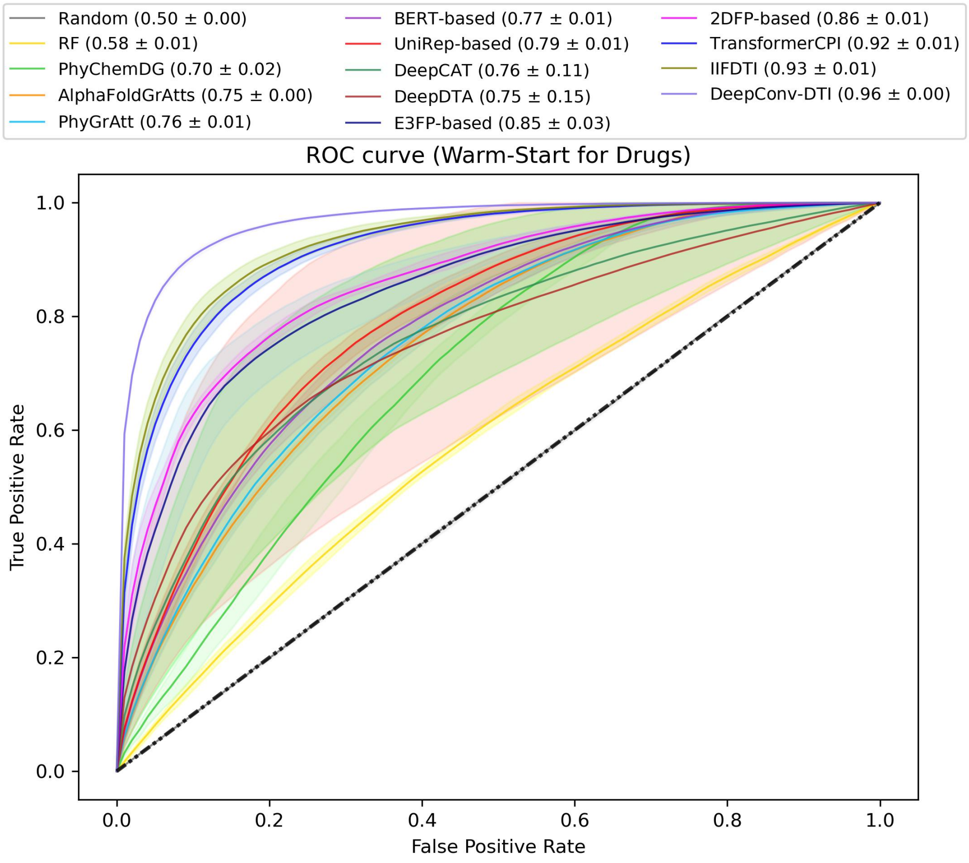

Supplementary Figure 6: Comparison of ROC curves for various models on the large aggregated dataset, split using the warm-start splitting scenarios for compounds.

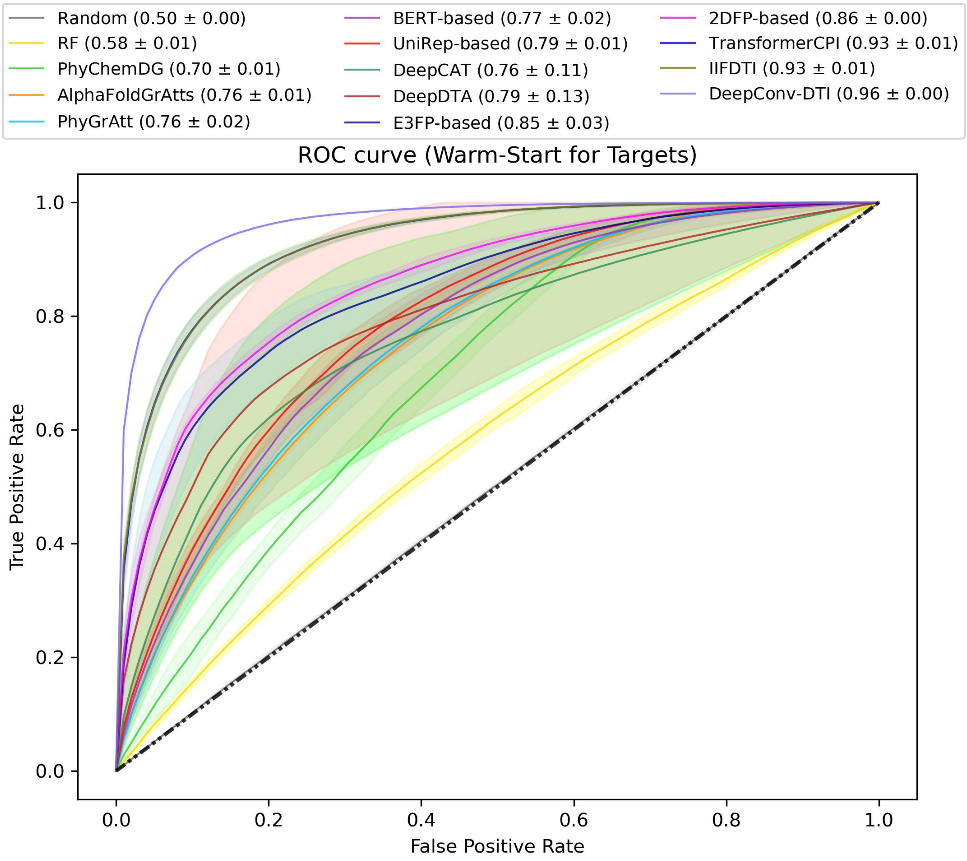

Supplementary Figure 7: Comparison of ROC curves for various models on the large aggregated dataset, split using the warm-start splitting scenarios for targets.

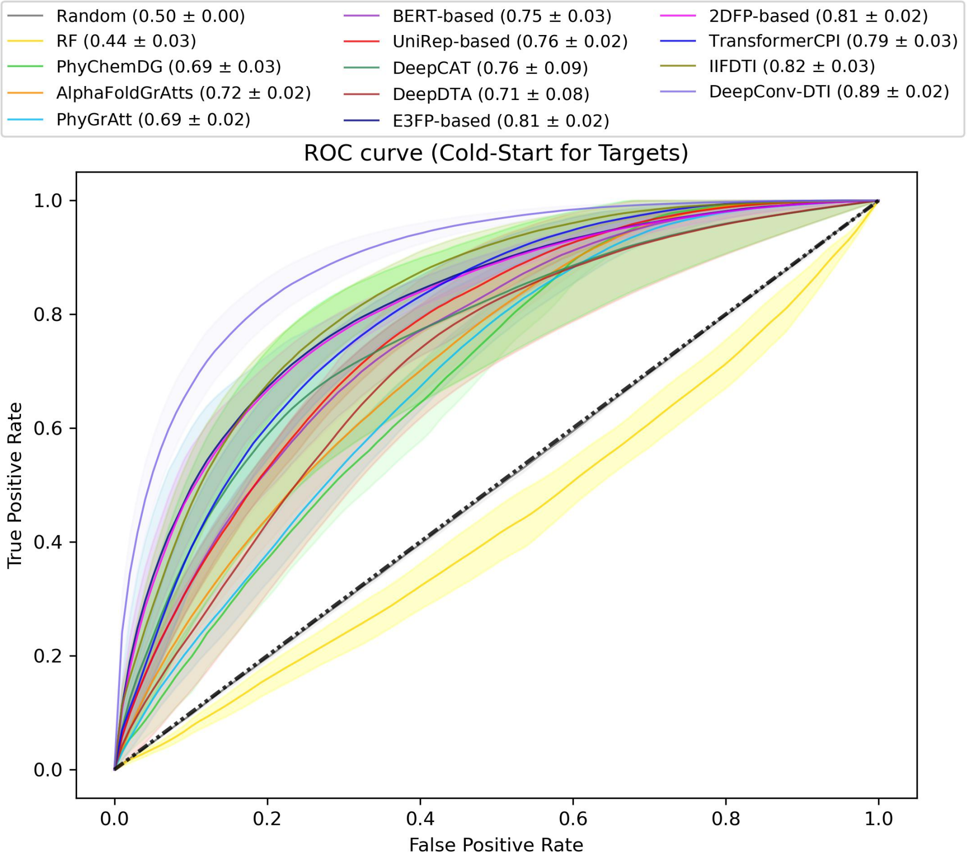

Supplementary Figure 8: Comparison of ROC curves for various models on the large aggregated dataset, split using the cold-start splitting scenarios for targets.

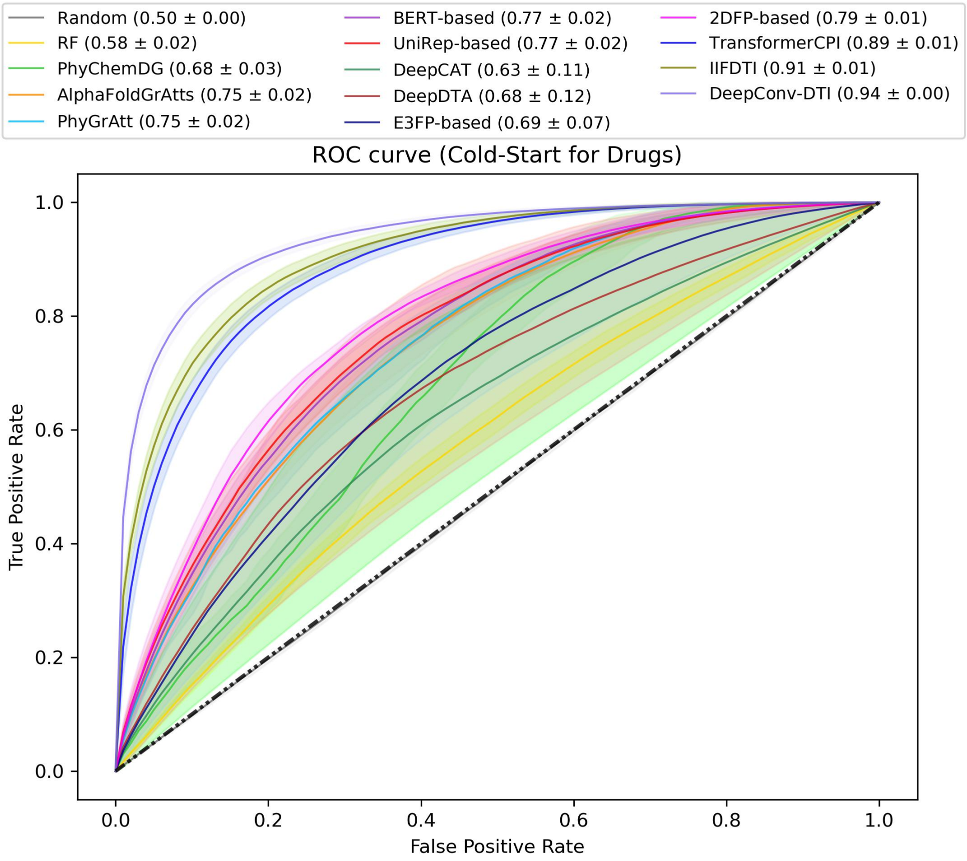

Supplementary Figure 9: Comparison of ROC curves for various models on the large aggregated dataset, split using the cold-start splitting scenarios for compounds.

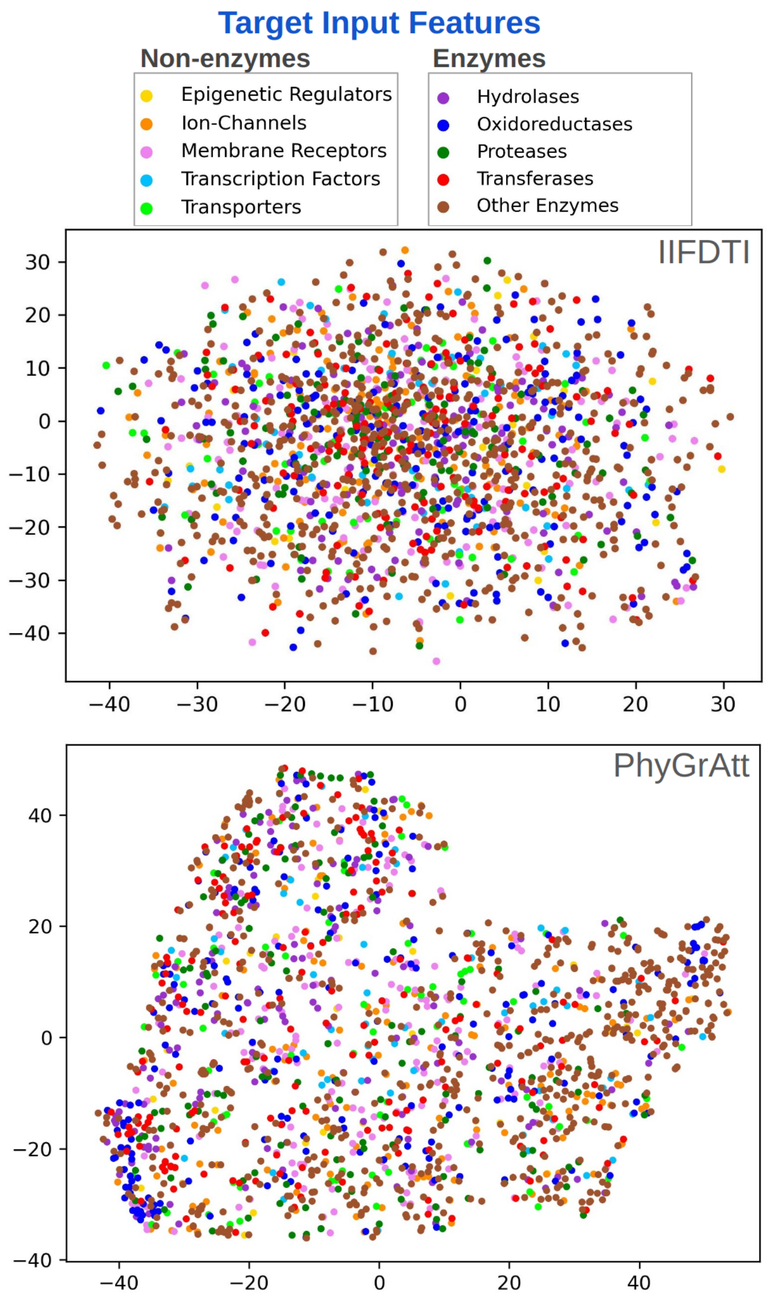

Supplementary Figure 10: The t-SNE plots of the target representative vectors obtained from the IIFDTI and PhyGrAtt models after training across the large aggregated dataset.

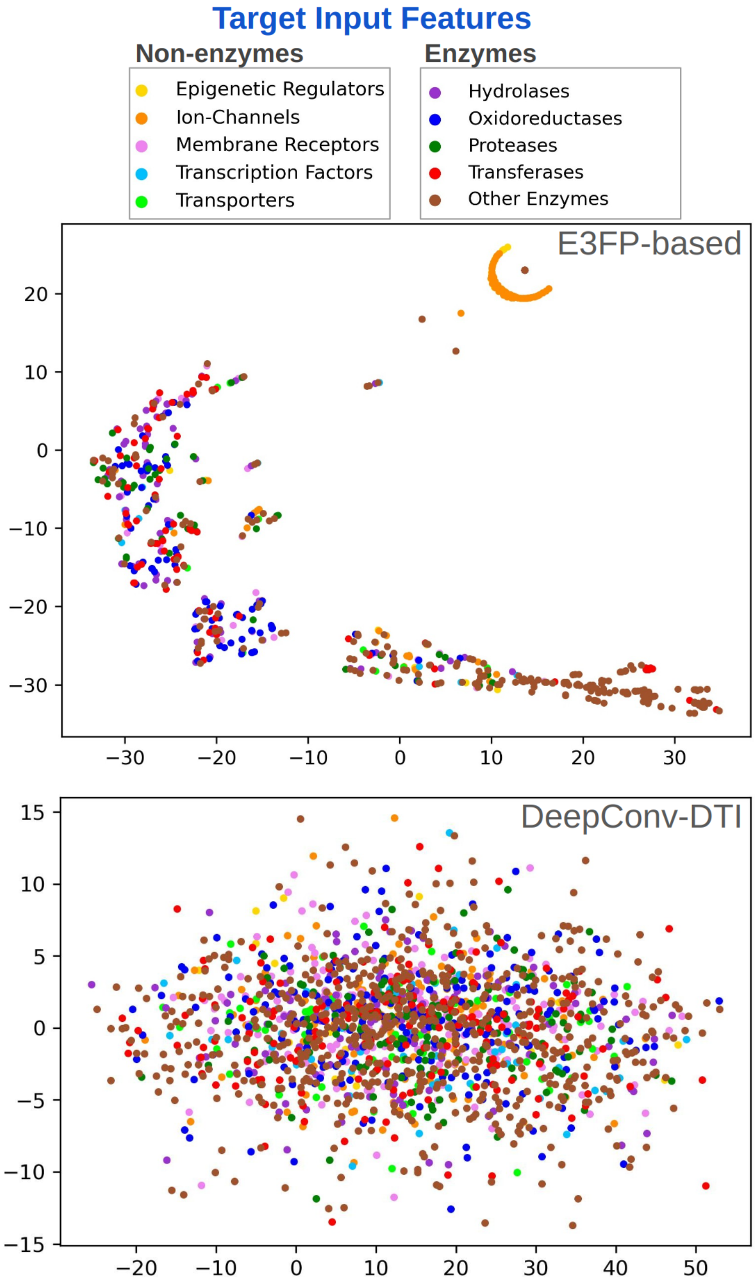

Supplementary Figure 11: The t-SNE plots of the target representative vectors obtained from the E3FP-based and DeepConv-DTI models after training across the large aggregated dataset.

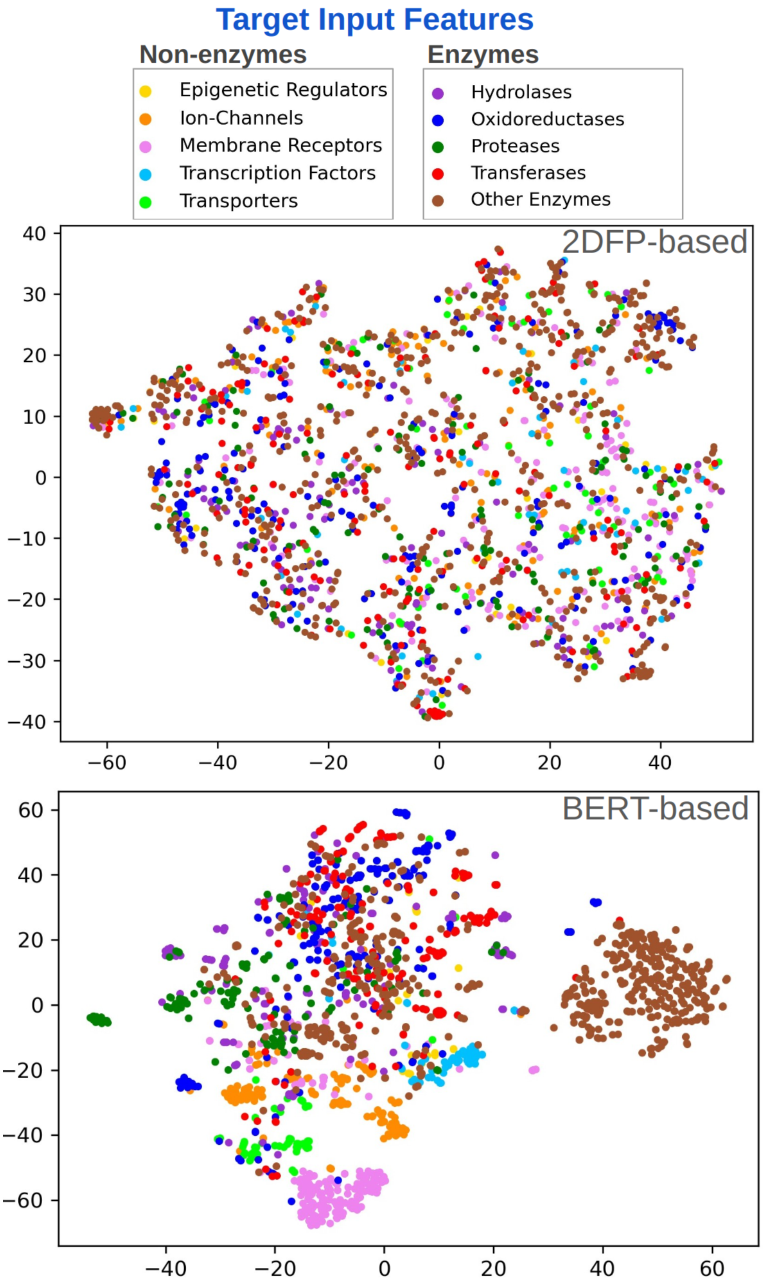

Supplementary Figure 12: The t-SNE plots of the target representative vectors obtained from the 2DFP-based and BERT-based models after training across the large aggregated dataset.

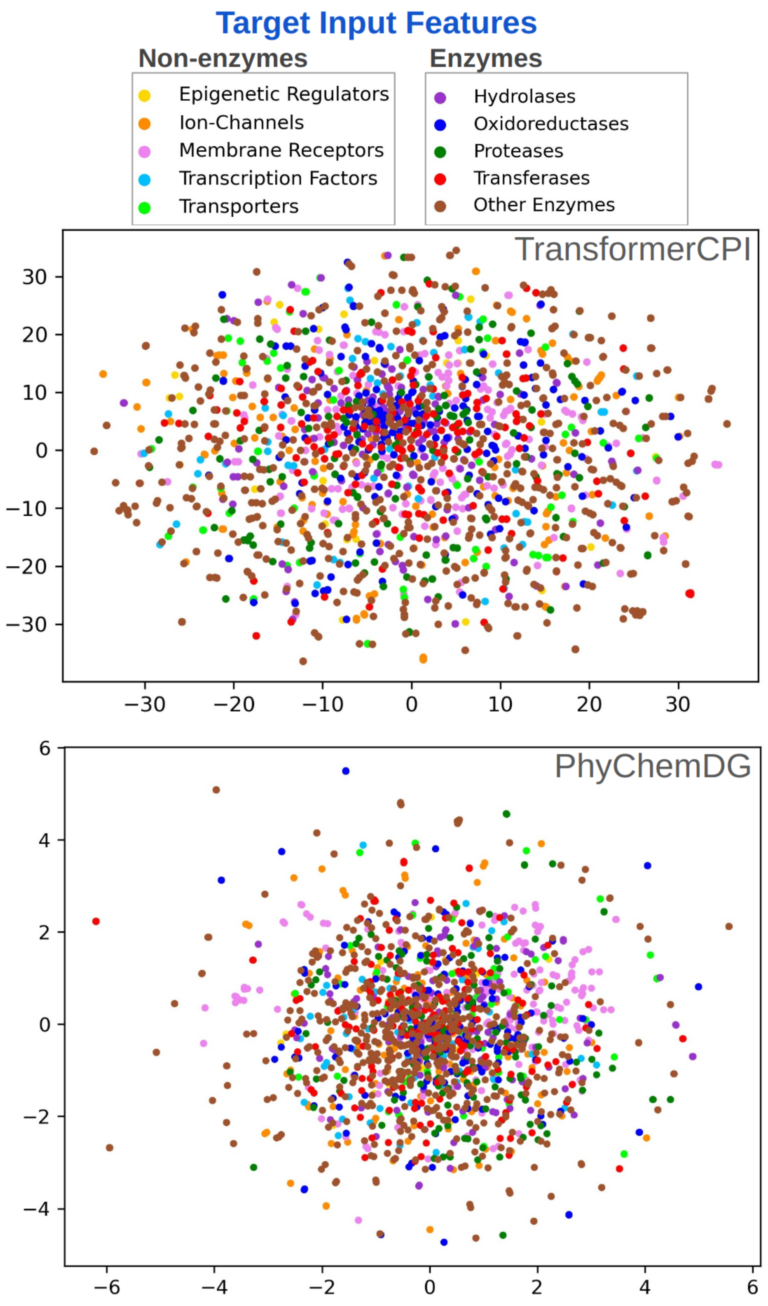

Supplementary Figure 13: The t-SNE plots of the target representative vectors obtained from the TransformerCPI and PhyChemDG models after training across the large aggregated dataset.

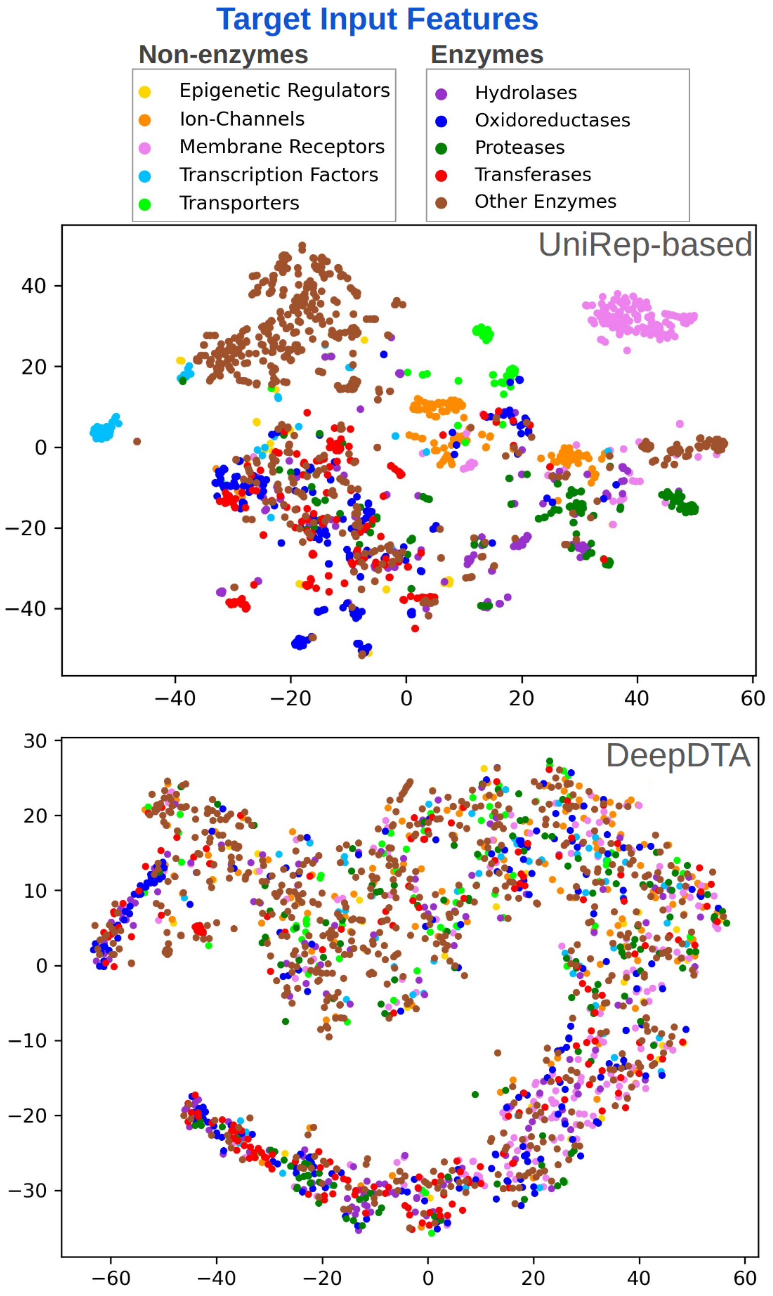

Supplementary Figure 14: The t-SNE plots of the target representative vectors obtained from the UniRep-based and DeepDTA models after training across the large aggregated dataset.

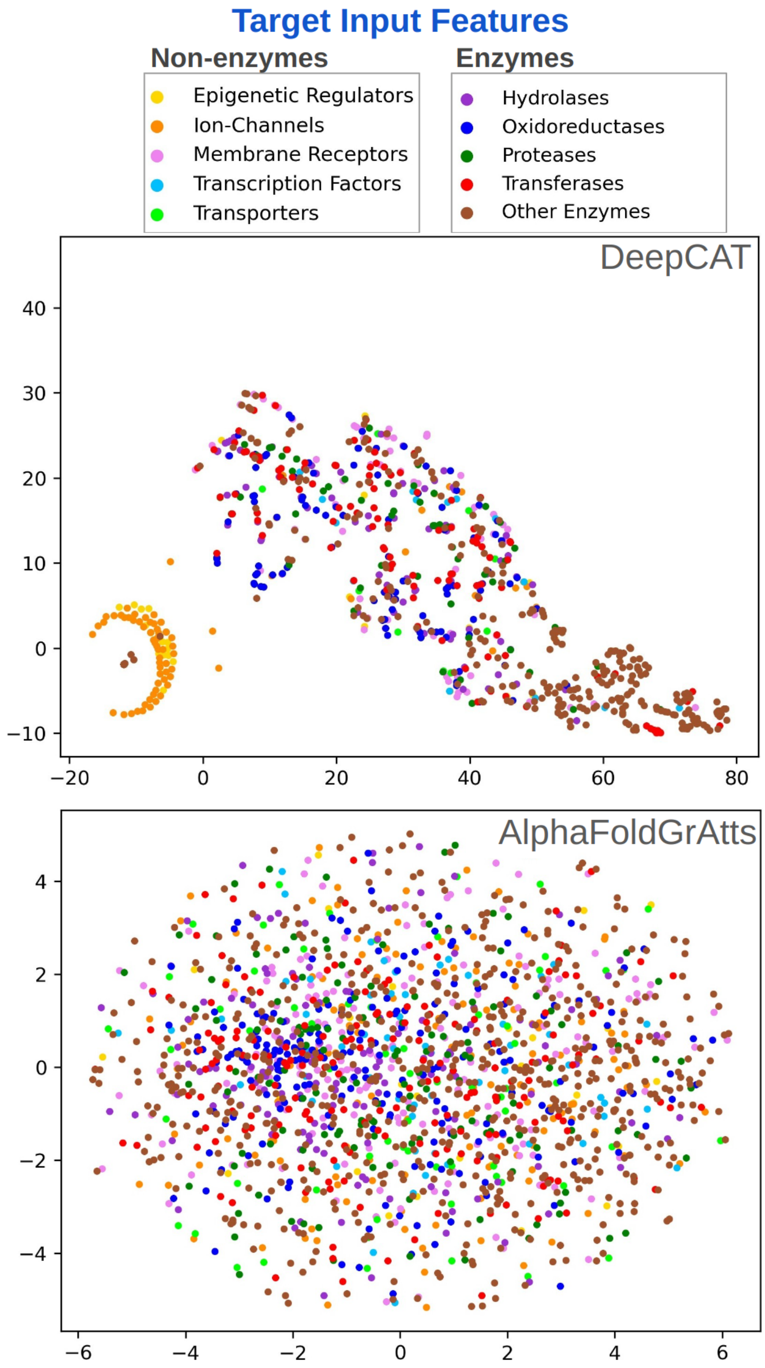

Supplementary Figure 15: The t-SNE plots of the target representative vectors obtained from the DeepCAT and AlphaFoldGrAtts models after training across the large aggregated dataset.

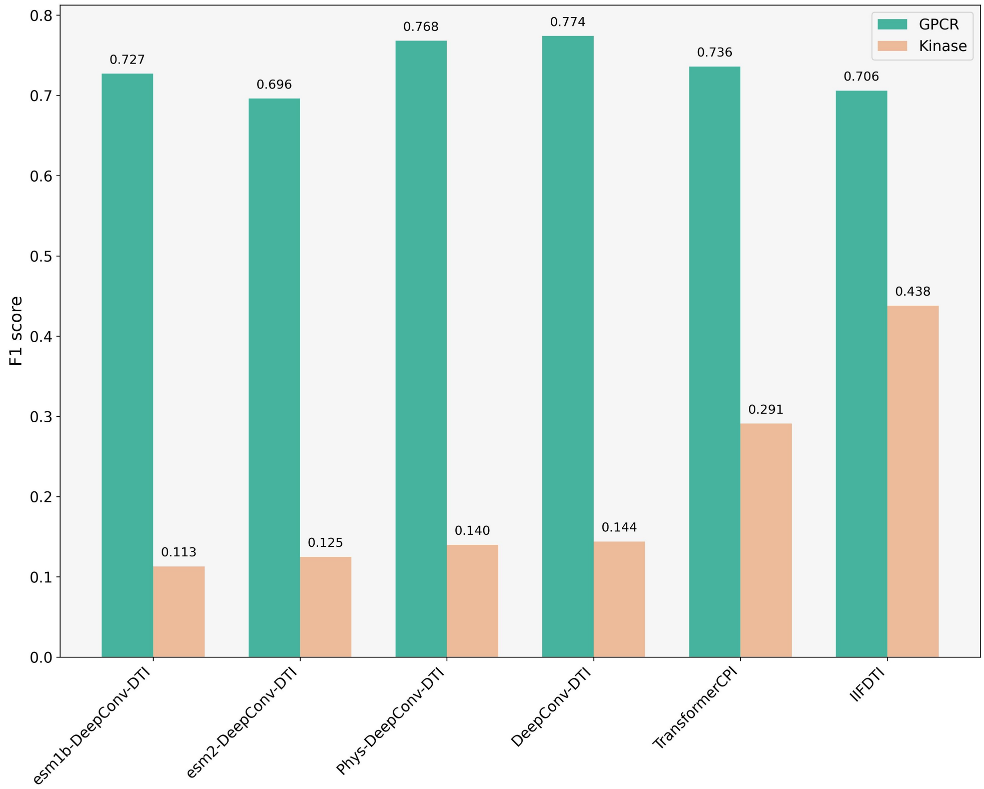

Supplementary Figure 16: Comparison of models across the label reversal dataset in terms of F1 score.

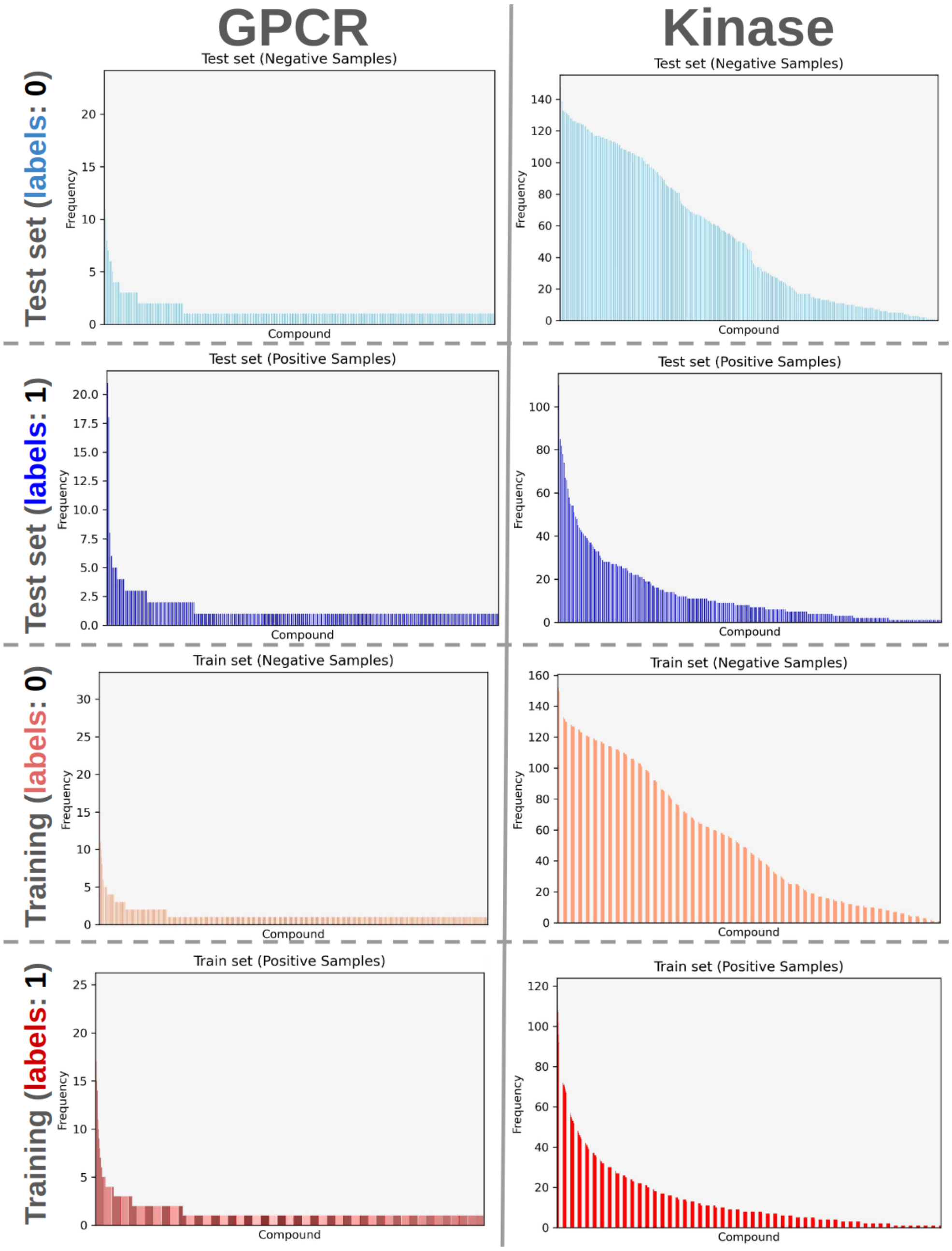

Supplementary Figure 17: Distribution of compound frequencies contributing to negative and positive CTIs in the GPCR and Kinase label reversal training and test sets.

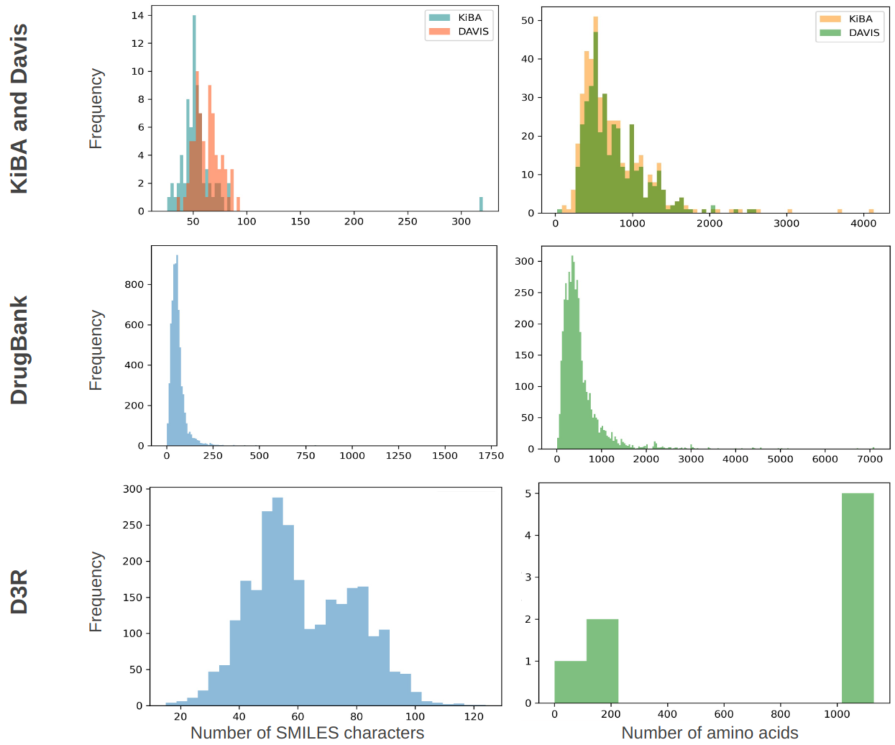

Supplementary Figure 18: The frequency distribution of compounds and targets in different datasets based on the number of SMILES characters and the number of amino acids, respectively.

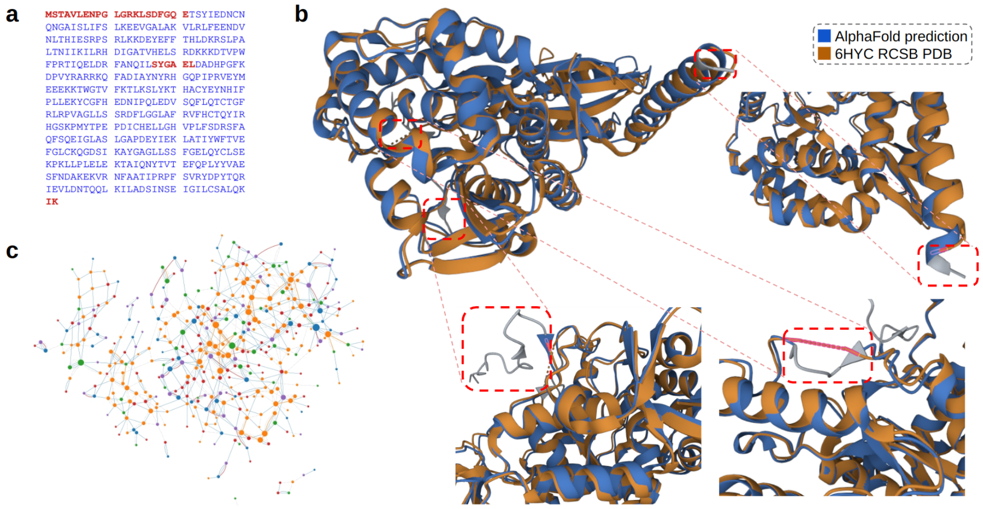

Supplementary Figure 19: (a) The amino acid sequence of the PH4H protein. Amino acids that do not appear in the 6HYC PDB file are highlighted in red. (b) The real (orange) and AlphaFold-predicted (blue) 3D structure of the PH4H protein. Missing amino acids, predicted by AlphaFold, are shown in gray. (c) The residue interaction network of the PH4H protein extracted using RINGv3.0.

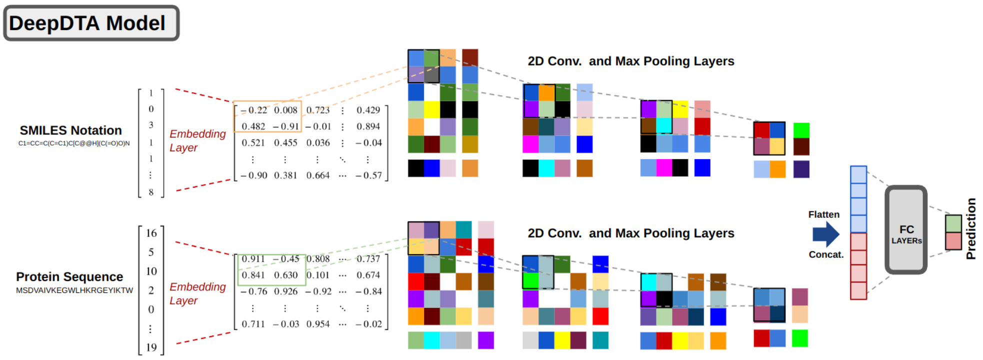

Supplementary Figure 20: DeepDTA: Utilizing learnable embeddings (targets and compounds).

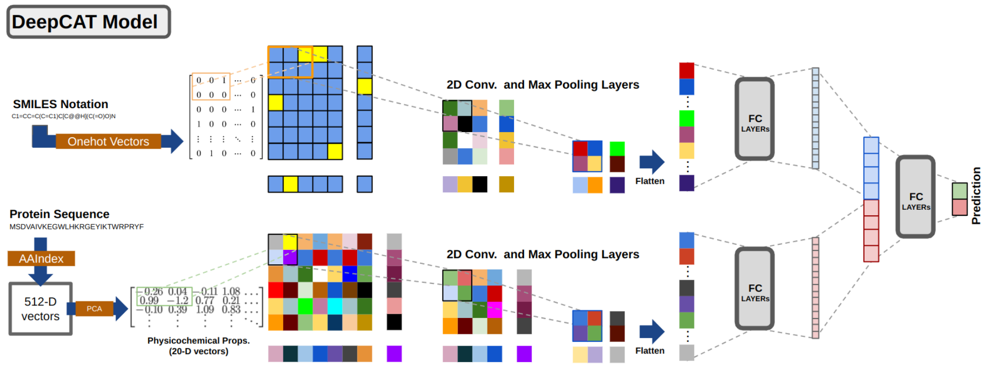

Supplementary Figure 21: DeepCAT: Utilizing physicochemical properties (targets) and onehot vectors (compounds).

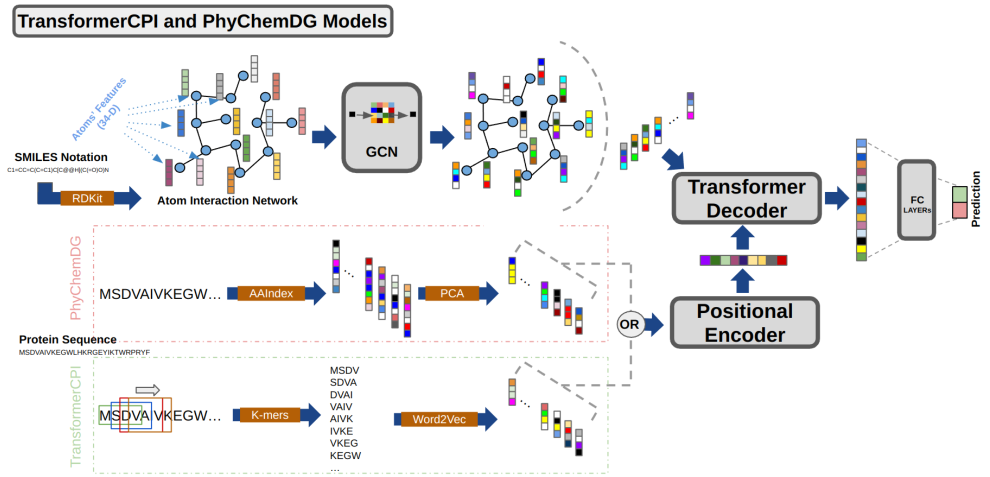

Supplementary Figure 22: TransformerCPI: Utilizing k-mers and Word2Vec (targets) and atoms' interaction network (compounds). PhyChemDG: Utilizing physicochemical properties (target) and atoms' interaction network (compounds)

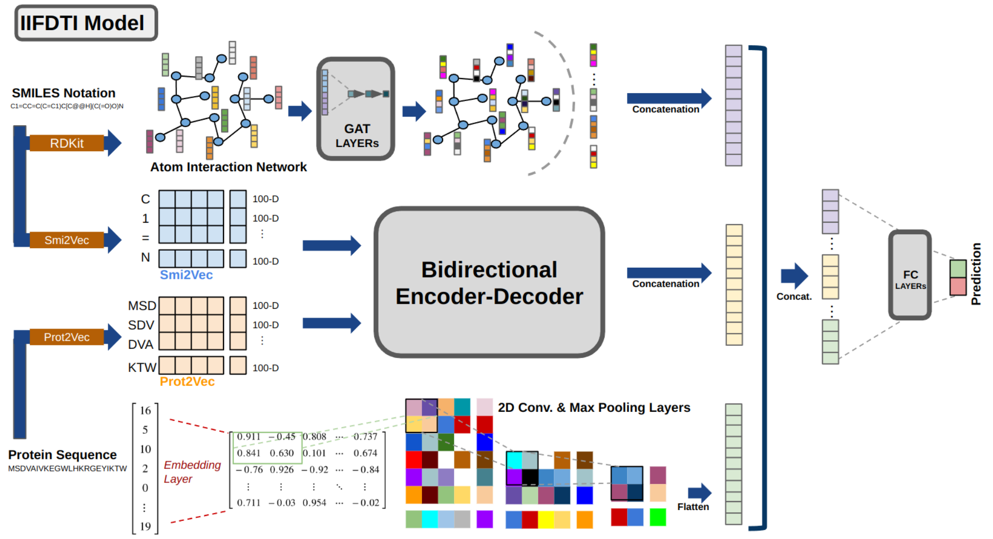

Supplementary Figure 23: IIFDTI: Utilizing learnable embeddings and Prot2Vec (targets) and atoms' interaction network and Smi2Vec (compounds)

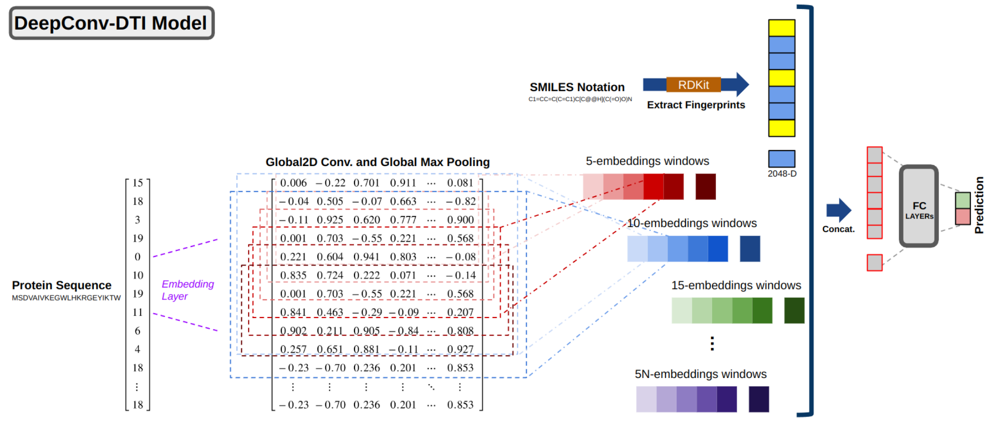

Supplementary Figure 24: DeepConv-DTI: Utilizing learnable embeddings (targets) and drug fingerprints (compounds)

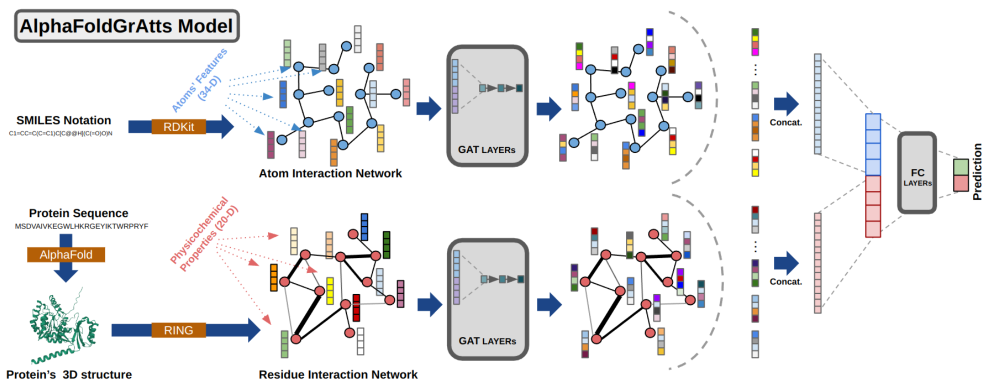

Supplementary Figure 25: AlphaFoldGrAtts: Utilizing residue interaction network (targets) and atoms' interaction network (compounds)

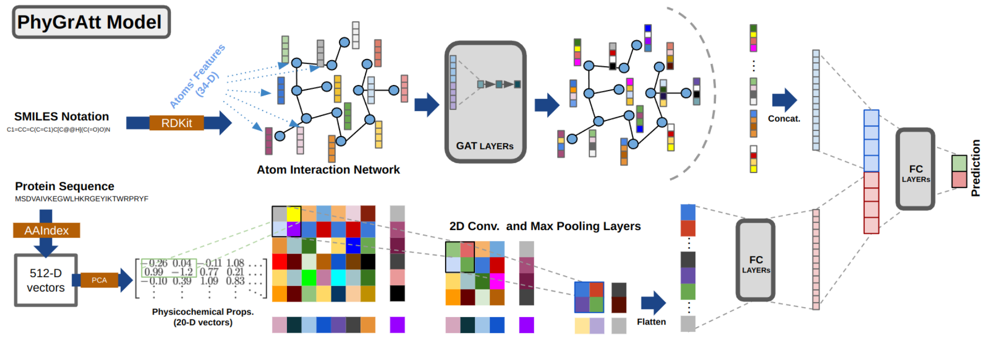

Supplementary Figure 26: PhyGrAtt: Utilizing physicochemical properties (targets) and atoms' interaction network (compounds)

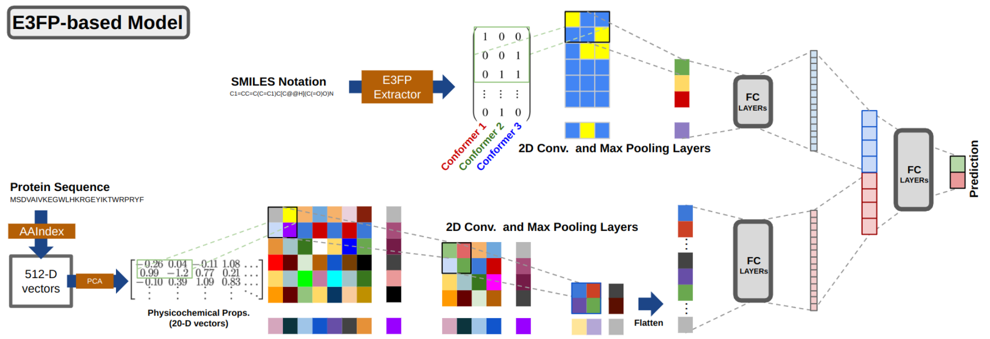

Supplementary Figure 27: The E3FP-based model: Utilizing physicochemical properties (targets) and 3D drug fingerprints (compounds)

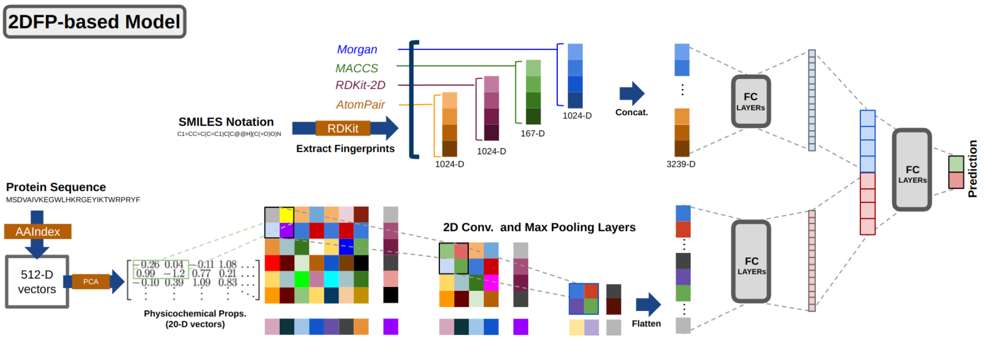

Supplementary Figure 28: The 2DFP-based model: Utilizing physicochemical properties (targets) and 2D drug fingerprints (compounds)

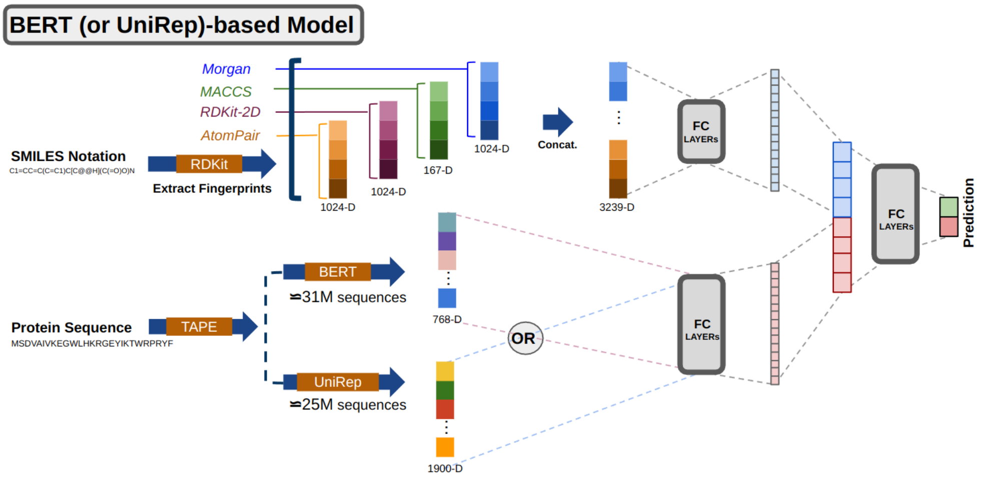

Supplementary Figure 29: The BERT (and UniRep)-based models: Utilizing learned, BERT-based or UniRep-based, embeddings (targets) and 2D drug fingerprints (compounds)

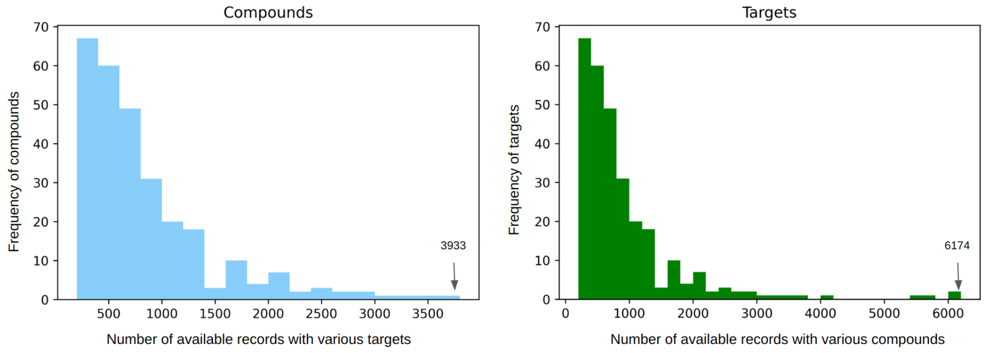

Supplementary Figure 30: The frequency distribution of compounds and target in the large aggregated dataset.

Supplementary Table 1: Warm-start for compounds splitting

Folds	Dataset	Negative	Positive	Ratio N/P	Avg No. Rot. bonds
**Fold 0**	Training Set	216274	87499	2.5	7.2
	Test Set	23781	9972	2.4	7.1
**Fold 1**	Training Set	216200	87573	2.5	7.2
	Test Set	23855	9898	2.4	7.0
**Fold 2**	Training Set	215997	87776	2.5	7.2
	Test Set	24058	9695	2.5	7.1
**Fold 3**	Training Set	215880	87893	2.5	7.2
	Test Set	24175	9578	2.5	7.1
**Fold 4**	Training Set	216145	87628	2.5	7.2
	Test Set	23910	9843	2.4	7.1
**Fold 5**	Training Set	215908	87865	2.5	7.2
	Test Set	24147	9606	2.5	7.1
**Fold 6**	Training Set	216306	87468	2.5	7.2
	Test Set	23749	10003	2.4	7.1
**Fold 7**	Training Set	215741	88033	2.5	7.2
	Test Set	24314	9438	2.6	7.1
**Fold 8**	Training Set	216093	87681	2.5	7.2
	Test Set	23962	9790	2.4	7.1
**Fold 9**	Training Set	215951	87823	2.6	7.2
	Test Set	24104	9648	2.5	7.1

Supplementary Table 2: Warm-start for targets splitting

Folds	Dataset	Negative	Positive	Ratio N/P	Avg No. Rot. bonds
**Fold 0**	Training Set	215967	87806	2.5	7.2
	Test Set	24088	9665	2.5	7.1
**Fold 1**	Training Set	216279	87494	2.5	7.2
	Test Set	23776	9977	2.4	7.1
**Fold 2**	Training Set	215951	87822	2.5	7.2
	Test Set	24104	9649	2.5	7.0
**Fold 3**	Training Set	216259	87514	2.5	7.2
	Test Set	23796	9957	2.4	7.1
**Fold 4**	Training Set	215812	87961	2.5	7.2
	Test Set	24243	9510	2.5	7.0
**Fold 5**	Training Set	216116	87657	2.5	7.2
	Test Set	23939	9814	2.4	7.0
**Fold 6**	Training Set	215808	87966	2.5	7.2
	Test Set	24247	9505	2.6	7.1
**Fold 7**	Training Set	216146	87628	2.5	7.2
	Test Set	23909	9843	2.4	7.1
**Fold 8**	Training Set	215859	87915	2.5	7.2
	Test Set	24196	9556	2.5	7.1
**Fold 9**	Training Set	216298	87476	2.5	7.2
	Test Set	23757	9995	2.4	7.1

Supplementary Table 3: Cold-start for compounds splitting

Folds	Dataset	Negative	Positive	Ratio N/P	Avg No. Rot. bonds
**Fold 0**	Training Set	214805	88968	2.4	7.2
	Test Set	25250	8503	2.9	7.6
**Fold 1**	Training Set	215316	88457	2.4	7.2
	Test Set	24739	9014	2.7	7.8
**Fold 2**	Training Set	215139	88634	2.4	7.2
	Test Set	24916	8837	2.8	7.7
**Fold 3**	Training Set	215452	88321	2.4	7.2
	Test Set	24603	9150	2.7	7.6
**Fold 4**	Training Set	215689	88084	2.4	7.2
	Test Set	24366	9387	2.6	7.5
**Fold 5**	Training Set	216459	87314	2.5	7.2
	Test Set	23596	10157	2.3	7.2
**Fold 6**	Training Set	216354	87419	2.5	7.2
	Test Set	23701	10052	2.4	7.0
**Fold 7**	Training Set	217028	86745	2.5	7.3
	Test Set	23027	10726	2.1	7.0
**Fold 8**	Training Set	217270	86503	2.5	7.3
	Test Set	22785	10968	2.1	6.9
**Fold 9**	Training Set	216983	86794	2.5	7.3
	Test Set	23072	10677	2.2	6.7

Supplementary Table 4: Cold-start for targets splitting

Folds	Dataset	Negative	Positive	Ratio N/P	Avg No. Rot. bonds
**Fold 0**	Training Set	215564	88209	2.4	7.3
	Test Set	24491	9262	2.6	6.9
**Fold 1**	Training Set	217813	85960	2.5	7.3
	Test Set	22242	11511	1.9	6.9
**Fold 2**	Training Set	216296	87477	2.5	7.2
	Test Set	23759	9994	2.4	6.9
**Fold 3**	Training Set	217464	86309	2.5	7.3
	Test Set	22591	11162	2.0	6.9
**Fold 4**	Training Set	216258	87515	2.5	7.1
	Test Set	23797	9956	2.4	7.4
**Fold 5**	Training Set	214611	89162	2.4	7.2
	Test Set	25444	8309	3.0	7.3
**Fold 6**	Training Set	216959	86814	2.5	7.2
	Test Set	23096	10657	2.2	7.1
**Fold 7**	Training Set	214874	88899	2.4	7.1
	Test Set	25181	8572	2.9	7.8
**Fold 8**	Training Set	215640	88133	2.4	7.3
	Test Set	24415	9338	2.6	6.7
**Fold 9**	Training Set	215016	88761	2.6	7.2
	Test Set	25039	8710	2.9	7.0

Supplementary Table 5: Warm-start for compounds: performance details in various folds

**Fold-0**	Accuracy	AUC ROC	AUC PR	F1 score	MCC
TransformerCPI	0.862	0.928	0.849	0.780	0.682
RF	0.658	0.581	0.351	0.292	0.084
Random	0.501	0.501	0.284	0.362	0.002
PhyChemDG	0.597	0.709	0.447	0.552	0.312
PhyGrAtt	0.740	0.775	0.554	0.481	0.325
AlphaFoldGrAtts	0.727	0.757	0.527	0.330	0.234
E3FP-based	0.751	0.772	0.522	0.541	0.371
2DFP-based	0.827	0.866	0.757	0.647	0.547
DeepCAT	0.772	0.813	0.616	0.510	0.385
DeepDTA	0.723	0.581	0.393	0.128	0.128
BERT-based	0.744	0.749	0.561	0.390	0.300
UniRep-based	0.753	0.797	0.598	0.453	0.339
DeepConv-DTI	0.914	0.967	0.933	0.850	0.791
IIFDTI	0.876	0.937	0.869	0.781	0.695
**Fold-1**	Accuracy	AUC ROC	AUC PR	F1 score	MCC
TransformerCPI	0.856	0.930	0.859	0.775	0.675
RF	0.654	0.583	0.350	0.322	0.099
PhyChemDG	0.609	0.659	0.394	0.502	0.232
Random	0.497	0.497	0.265	0.347	-0.003
PhyGrAtt	0.729	0.757	0.528	0.371	0.253
E3FP-based	0.838	0.883	0.756	0.703	0.592
2DFP-based	0.830	0.858	0.725	0.641	0.540
DeepCAT	0.780	0.829	0.617	0.520	0.392
DeepDTA	0.814	0.859	0.705	0.589	0.487
AlphaFoldGrAtts	0.731	0.754	0.535	0.281	0.229
UniRep-based	0.707	0.790	0.547	0.000	0.000
BERT-based	0.742	0.770	0.568	0.387	0.289
DeepConv-DTI	0.912	0.965	0.929	0.847	0.786
IIFDTI	0.875	0.934	0.866	0.783	0.696
**Fold-2**	Accuracy	AUC ROC	AUC PR	F1 score	MCC
TransformerCPI	0.832	0.915	0.817	0.740	0.627
RF	0.660	0.587	0.345	0.305	0.091
PhyChemDG	0.660	0.680	0.409	0.376	0.144
Random	0.497	0.498	0.288	0.361	-0.007
PhyGrAtt	0.738	0.760	0.536	0.361	0.261
E3FP-based	0.829	0.857	0.741	0.678	0.569
2DFP-based	0.819	0.851	0.677	0.673	0.549
DeepCAT	0.784	0.818	0.665	0.514	0.419
DeepDTA	0.763	0.794	0.607	0.434	0.349

AlphaFoldGrAtts	0.737	0.755	0.522	0.315	0.241
BERT-based	0.752	0.784	0.575	0.425	0.317
UniRep-based	0.746	0.796	0.568	0.422	0.301
DeepConv-DTI	0.914	0.967	0.929	0.848	0.788
IIFDTI	0.869	0.929	0.853	0.767	0.677
**Fold-3**	Accuracy	AUC ROC	AUC PR	F1 score	MCC
TransformerCPI	0.850	0.918	0.817	0.755	0.651
RF	0.659	0.576	0.339	0.308	0.091
PhyChemDG	0.675	0.710	0.431	0.457	0.227
Random	0.497	0.498	0.269	0.350	-0.004
PhyGrAtt	0.742	0.765	0.530	0.449	0.302
E3FP-based	0.819	0.854	0.716	0.630	0.525
2DFP-based	0.823	0.859	0.716	0.654	0.538
DeepCAT	0.795	0.820	0.647	0.555	0.438
DeepDTA	0.815	0.859	0.710	0.623	0.506
AlphaFoldGrAtts	0.734	0.743	0.503	0.277	0.215
UniRep-based	0.758	0.787	0.586	0.440	0.330
BERT-based	0.749	0.765	0.553	0.391	0.293
DeepConv-DTI	0.914	0.965	0.926	0.849	0.789
IIFDTI	0.860	0.920	0.838	0.743	0.649
**Fold-4**	Accuracy	AUC ROC	AUC PR	F1 score	MCC
TransformerCPI	0.859	0.925	0.842	0.770	0.670
RF	0.663	0.589	0.351	0.306	0.099
PhyChemDG	0.713	0.732	0.473	0.387	0.226
Random	0.500	0.505	0.300	0.375	0.004
PhyGrAtt	0.737	0.765	0.552	0.389	0.276
E3FP-based	0.825	0.864	0.759	0.658	0.557
2DFP-based	0.825	0.867	0.756	0.677	0.563
DeepCAT	0.784	0.826	0.674	0.559	0.440
AlphaFoldGrAtts	0.731	0.752	0.524	0.316	0.233
BERT-based	0.748	0.781	0.582	0.401	0.308
UniRep-based	0.752	0.804	0.591	0.467	0.336
DeepDTA	0.704	0.500	0.296	0.000	0.000
DeepConv-DTI	0.916	0.966	0.931	0.852	0.793
IIFDTI	0.871	0.934	0.863	0.781	0.690
**Fold-5**	Accuracy	AUC ROC	AUC PR	F1 score	MCC
TransformerCPI	0.845	0.912	0.809	0.737	0.628
RF	0.659	0.587	0.343	0.307	0.090
PhyChemDG	0.714	0.723	0.468	0.341	0.197
Random	0.503	0.502	0.272	0.355	0.007
PhyGrAtt	0.726	0.721	0.476	0.147	0.155
E3FP-based	0.827	0.866	0.724	0.680	0.562
2DFP-based	0.819	0.853	0.709	0.627	0.516

AlphaFoldGrAtts	0.740	0.757	0.528	0.277	0.236
UniRep-based	0.761	0.780	0.582	0.448	0.341
BERT-based	0.753	0.788	0.566	0.429	0.315
DeepCAT	0.729	0.500	0.271	0.000	0.000
DeepDTA	0.729	0.503	0.272	0.000	0.000
DeepConv-DTI	0.915	0.966	0.928	0.849	0.790
IIFDTI	0.847	0.908	0.808	0.722	0.617
**Fold-6**	Accuracy	AUC ROC	AUC PR	F1 score	MCC
TransformerCPI	0.831	0.913	0.825	0.742	0.624
RF	0.655	0.581	0.356	0.309	0.093
PhyChemDG	0.687	0.697	0.442	0.315	0.144
Random	0.503	0.502	0.285	0.365	0.006
PhyGrAtt	0.741	0.769	0.561	0.475	0.323
E3FP-based	0.824	0.862	0.750	0.654	0.550
2DFP-based	0.824	0.867	0.751	0.644	0.542
DeepCAT	0.784	0.837	0.674	0.528	0.418
DeepDTA	0.820	0.870	0.746	0.632	0.529
AlphaFoldGrAtts	0.727	0.755	0.535	0.265	0.221
BERT-based	0.704	0.788	0.546	0.000	0.000
UniRep-based	0.753	0.795	0.607	0.442	0.338
DeepConv-DTI	0.917	0.969	0.935	0.857	0.799
IIFDTI	0.870	0.933	0.864	0.774	0.684
**Fold-7**	Accuracy	AUC ROC	AUC PR	F1 score	MCC
TransformerCPI	0.851	0.916	0.818	0.745	0.641
RF	0.672	0.586	0.349	0.328	0.119
PhyChemDG	0.700	0.709	0.432	0.330	0.161
Random	0.502	0.502	0.267	0.350	0.004
PhyGrAtt	0.737	0.752	0.516	0.477	0.308
E3FP-based	0.815	0.843	0.706	0.631	0.521
2DFP-based	0.827	0.863	0.716	0.661	0.546
DeepCAT	0.735	0.657	0.391	0.059	0.079
DeepDTA	0.806	0.848	0.618	0.654	0.521
AlphaFoldGrAtts	0.735	0.754	0.505	0.224	0.190
UniRep-based	0.726	0.782	0.520	0.138	0.132
BERT-based	0.757	0.771	0.556	0.411	0.312
DeepConv-DTI	0.912	0.964	0.922	0.843	0.782
IIFDTI	0.874	0.934	0.857	0.769	0.683
**Fold-8**	Accuracy	AUC ROC	AUC PR	F1 score	MCC
TransformerCPI	0.839	0.922	0.839	0.753	0.644
RF	0.663	0.591	0.357	0.322	0.109
PhyChemDG	0.682	0.710	0.443	0.429	0.210
Random	0.495	0.496	0.287	0.362	-0.009
PhyGrAtt	0.740	0.762	0.539	0.423	0.292

E3FP-based	0.812	0.839	0.709	0.619	0.515
2DFP-based	0.822	0.866	0.760	0.643	0.542
DeepDTA	0.816	0.867	0.747	0.630	0.525
AlphaFoldGrAtts	0.736	0.752	0.519	0.325	0.249
BERT-based	0.755	0.768	0.574	0.426	0.328
UniRep-based	0.753	0.787	0.585	0.430	0.324
DeepCAT	0.710	0.652	0.395	0.000	0.000
DeepConv-DTI	0.914	0.967	0.931	0.849	0.790
IIFDTI	0.877	0.935	0.861	0.783	0.697

**Fold-9**	Accuracy	AUC ROC	AUC PR	F1 score	MCC
TransformerCPI	0.829	0.911	0.818	0.736	0.621
RF	0.653	0.569	0.327	0.278	0.062
PhyChemDG	0.675	0.707	0.430	0.456	0.225
Random	0.498	0.499	0.275	0.352	-0.004
PhyGrAtt	0.737	0.754	0.521	0.450	0.296
E3FP-based	0.810	0.835	0.628	0.678	0.544
2DFP-based	0.824	0.858	0.726	0.644	0.535
DeepDTA	0.821	0.866	0.724	0.647	0.531
DeepCAT	0.787	0.837	0.657	0.529	0.414
AlphaFoldGrAtts	0.731	0.753	0.516	0.235	0.197
UniRep-based	0.752	0.791	0.575	0.427	0.314
BERT-based	0.714	0.777	0.516	0.000	0.000
DeepConv-DTI	0.914	0.966	0.927	0.846	0.786
IIFDTI	0.868	0.928	0.851	0.762	0.671

Supplementary Table 6: Average performance of the models using warm-start splitting scenario for compounds.

**MODELS**	**Accuracy**	**AUC ROC**	**AUC PR**	**F1 score**	**MCC**
**UniRep-based**	0.746	0.791	0.576	0.367	0.276
**DeepConv-DTI**	0.914	0.966	0.929	0.849	0.789
**IIFDTI**	0.869	0.929	0.853	0.766	0.676
**TransformerCPI**	0.845	0.919	0.829	0.753	0.646
**BERT-based**	0.742	0.774	0.560	0.326	0.246
**AlphaFoldGrAtts**	0.733	0.753	0.521	0.285	0.225
**PhyGrAtt**	0.737	0.758	0.531	0.402	0.279
**E3FP-based**	0.815	0.847	0.701	0.647	0.531
**2DFP-based**	0.824	0.861	0.729	0.651	0.542
**DeepCAT**	0.766	0.759	0.561	0.377	0.298
**DeepDTA**	0.781	0.755	0.582	0.434	0.358
**Random**	0.500	0.500	0.279	0.358	0.000
**RF**	0.660	0.583	0.347	0.308	0.094
**PhyChemDG**	0.671	0.704	0.437	0.415	0.208

Supplementary Table 7: Warm-start for targets: performance details in various folds

**Fold-0**	Accuracy	AUC ROC	AUC PR	F1 score	MCC
TransformerCPI	0.878	0.937	0.868	0.789	0.703
RF	0.672	0.580	0.345	0.289	0.096
Random	0.500	0.503	0.291	0.368	0.004
PhyChemDG	0.666	0.680	0.393	0.325	0.114
PhyGrAtt	0.744	0.776	0.549	0.511	0.343
E3FP-based	0.820	0.853	0.732	0.635	0.535
2DFP-based	0.812	0.848	0.723	0.609	0.508
DeepDTA	0.821	0.868	0.733	0.687	0.562
DeepCAT	0.784	0.815	0.583	0.623	0.472
AlphaFoldGrAtts	0.735	0.763	0.532	0.256	0.220
UniRep-based	0.754	0.800	0.583	0.449	0.329
BERT-based	0.756	0.787	0.585	0.449	0.333
DeepConv-DTI	0.916	0.967	0.931	0.853	0.794
IIFDTI	0.868	0.931	0.851	0.770	0.677
**Fold-1**	Accuracy	AUC ROC	AUC PR	F1 score	MCC
TransformerCPI	0.880	0.940	0.881	0.797	0.712
RF	0.657	0.569	0.340	0.282	0.076
PhyChemDG	0.673	0.682	0.448	0.420	0.194
Random	0.503	0.506	0.274	0.354	0.005
PhyGrAtt	0.738	0.766	0.545	0.438	0.300
E3FP-based	0.825	0.858	0.732	0.642	0.542
2DFP-based	0.830	0.861	0.728	0.656	0.549
DeepDTA	0.821	0.866	0.709	0.675	0.552
DeepCAT	0.785	0.805	0.551	0.631	0.483
AlphaFoldGrAtts	0.727	0.754	0.528	0.290	0.226
UniRep-based	0.753	0.789	0.604	0.437	0.334
BERT-based	0.704	0.770	0.511	0.000	-0.004
DeepConv-DTI	0.914	0.967	0.934	0.855	0.794
IIFDTI	0.873	0.936	0.873	0.782	0.693
**Fold-2**	Accuracy	AUC ROC	AUC PR	F1 score	MCC
TransformerCPI	0.858	0.924	0.841	0.757	0.657
RF	0.655	0.588	0.347	0.325	0.099
PhyChemDG	0.687	0.715	0.444	0.430	0.215
Random	0.503	0.508	0.291	0.369	0.010
PhyGrAtt	0.744	0.774	0.544	0.444	0.304
E3FP-based	0.821	0.860	0.743	0.657	0.548
2DFP-based	0.821	0.859	0.739	0.639	0.532
DeepDTA	0.716	0.500	0.284	0.000	0.000
DeepCAT	0.784	0.836	0.670	0.534	0.419
AlphaFoldGrAtts	0.730	0.759	0.521	0.186	0.183

UniRep-based	0.759	0.805	0.603	0.463	0.343
BERT-based	0.738	0.756	0.518	0.335	0.250
DeepConv-DTI	0.918	0.967	0.932	0.855	0.798
IIFDTI	0.848	0.910	0.816	0.717	0.616
**Fold-3**	Accuracy	AUC ROC	AUC PR	F1 score	MCC
TransformerCPI	0.864	0.926	0.847	0.772	0.675
RF	0.656	0.579	0.354	0.308	0.092
PhyChemDG	0.656	0.704	0.453	0.496	0.247
Random	0.502	0.503	0.274	0.354	0.005
PhyGrAtt	0.737	0.757	0.550	0.432	0.297
E3FP-based	0.827	0.865	0.729	0.677	0.561
2DFP-based	0.824	0.863	0.718	0.658	0.541
DeepDTA	0.814	0.858	0.707	0.624	0.506
DeepCAT	0.801	0.839	0.677	0.543	0.447
AlphaFoldGrAtts	0.727	0.748	0.524	0.274	0.217
UniRep-based	0.747	0.795	0.590	0.435	0.319
BERT-based	0.747	0.737	0.559	0.406	0.312
DeepConv-DTI	0.914	0.966	0.932	0.850	0.790
IIFDTI	0.871	0.932	0.864	0.776	0.685
**Fold-4**	Accuracy	AUC ROC	AUC PR	F1 score	MCC
TransformerCPI	0.859	0.922	0.829	0.750	0.652
RF	0.670	0.601	0.352	0.319	0.111
PhyChemDG	0.528	0.686	0.407	0.518	0.276
Random	0.499	0.498	0.284	0.361	-0.002
PhyGrAtt	0.733	0.731	0.493	0.211	0.192
E3FP-based	0.826	0.858	0.752	0.655	0.557
2DFP-based	0.818	0.854	0.679	0.666	0.542
DeepDTA	0.810	0.858	0.719	0.653	0.523
DeepCAT	0.717	0.500	0.283	0.000	0.000
AlphaFoldGrAtts	0.743	0.759	0.525	0.384	0.272
UniRep-based	0.759	0.780	0.578	0.433	0.328
BERT-based	0.753	0.786	0.562	0.429	0.313
DeepConv-DTI	0.915	0.965	0.926	0.844	0.786
IIFDTI	0.870	0.929	0.852	0.769	0.678
**Fold-5**	Accuracy	AUC ROC	AUC PR	F1 score	MCC
TransformerCPI	0.862	0.925	0.842	0.759	0.663
RF	0.653	0.575	0.350	0.333	0.104
PhyChemDG	0.687	0.696	0.422	0.364	0.170
Random	0.500	0.498	0.269	0.350	-0.001
PhyGrAtt	0.737	0.758	0.540	0.347	0.261
E3FP-based	0.821	0.844	0.720	0.632	0.530
2DFP-based	0.825	0.865	0.731	0.632	0.529
DeepDTA	0.813	0.857	0.691	0.658	0.530

DeepCAT	0.799	0.824	0.641	0.582	0.459
AlphaFoldGrAtts	0.733	0.755	0.527	0.292	0.232
UniRep-based	0.747	0.786	0.565	0.420	0.307
BERT-based	0.746	0.761	0.559	0.400	0.300
DeepConv-DTI	0.915	0.967	0.931	0.852	0.793
IIFDTI	0.864	0.926	0.850	0.766	0.671
**Fold-6**	Accuracy	AUC ROC	AUC PR	F1 score	MCC
TransformerCPI	0.862	0.926	0.843	0.758	0.661
RF	0.666	0.593	0.343	0.321	0.107
PhyChemDG	0.600	0.689	0.414	0.504	0.247
Random	0.498	0.497	0.284	0.364	-0.002
PhyGrAtt	0.756	0.782	0.553	0.478	0.339
E3FP-based	0.749	0.771	0.531	0.510	0.351
2DFP-based	0.822	0.860	0.737	0.664	0.547
DeepDTA	0.816	0.859	0.729	0.650	0.531
DeepCAT	0.790	0.794	0.638	0.577	0.451
AlphaFoldGrAtts	0.738	0.764	0.521	0.274	0.221
UniRep-based	0.757	0.776	0.562	0.428	0.321
BERT-based	0.755	0.787	0.561	0.437	0.320
DeepConv-DTI	0.915	0.965	0.926	0.848	0.789
IIFDTI	0.873	0.933	0.856	0.765	0.679
**Fold-7**	Accuracy	AUC ROC	AUC PR	F1 score	MCC
TransformerCPI	0.866	0.928	0.848	0.771	0.676
RF	0.668	0.580	0.351	0.298	0.101
PhyChemDG	0.644	0.717	0.466	0.509	0.257
Random	0.501	0.500	0.277	0.357	0.004
PhyGrAtt	0.735	0.762	0.521	0.417	0.281
E3FP-based	0.822	0.851	0.717	0.634	0.532
2DFP-based	0.825	0.861	0.727	0.635	0.533
DeepDTA	0.733	0.582	0.387	0.218	0.171
DeepCAT	0.731	0.576	0.339	0.234	0.169
AlphaFoldGrAtts	0.735	0.755	0.529	0.350	0.255
UniRep-based	0.751	0.781	0.587	0.422	0.320
BERT-based	0.741	0.789	0.559	0.416	0.294
DeepConv-DTI	0.914	0.966	0.930	0.850	0.789
IIFDTI	0.861	0.926	0.851	0.748	0.655
**Fold-8**	Accuracy	AUC ROC	AUC PR	F1 score	MCC
TransformerCPI	0.862	0.923	0.832	0.759	0.662
RF	0.662	0.594	0.345	0.308	0.093
PhyChemDG	0.648	0.717	0.448	0.515	0.275
Random	0.504	0.505	0.289	0.371	0.010
PhyGrAtt	0.746	0.777	0.545	0.435	0.301
E3FP-based	0.815	0.845	0.720	0.645	0.529

2DFP-based	0.824	0.864	0.750	0.650	0.546
DeepDTA	0.801	0.825	0.673	0.612	0.487
DeepCAT	0.794	0.825	0.668	0.590	0.466
AlphaFoldGrAtts	0.743	0.764	0.526	0.379	0.271
UniRep-based	0.750	0.800	0.564	0.430	0.308
BERT-based	0.745	0.781	0.551	0.388	0.281
DeepConv-DTI	0.915	0.967	0.928	0.844	0.787
IIFDTI	0.859	0.923	0.838	0.738	0.643
**Fold-9**	Accuracy	AUC ROC	AUC PR	F1 score	MCC
TransformerCPI	0.858	0.922	0.843	0.756	0.655
RF	0.651	0.568	0.344	0.300	0.080
PhyChemDG	0.686	0.708	0.441	0.319	0.147
Random	0.501	0.505	0.280	0.355	0.000
PhyGrAtt	0.711	0.713	0.478	0.099	0.121
E3FP-based	0.824	0.850	0.721	0.638	0.536
2DFP-based	0.823	0.859	0.722	0.653	0.538
DeepDTA	0.825	0.876	0.750	0.630	0.531
DeepCAT	0.789	0.818	0.648	0.547	0.426
AlphaFoldGrAtts	0.722	0.751	0.530	0.213	0.193
UniRep-based	0.704	0.780	0.531	0.000	0.000
BERT-based	0.743	0.783	0.574	0.400	0.301
DeepConv-DTI	0.912	0.966	0.930	0.849	0.787
IIFDTI	0.866	0.929	0.861	0.774	0.679

Supplementary Table 8: Average performance of the models using warm-start splitting scenario for targets.

MODELS	Accuracy	AUC ROC	AUC PR	F1 score	MCC
**UniRep-based**	0.748	0.789	0.577	0.392	0.291
**DeepConv-DTI**	0.915	0.966	0.930	0.850	0.791
**IIFDTI**	0.865	0.928	0.851	0.760	0.668
**TransformerCPI**	0.865	0.927	0.847	0.767	0.672
**BERT-based**	0.743	0.774	0.554	0.366	0.270
**AlphaFoldGrAtts**	0.733	0.757	0.526	0.290	0.229
**PhyGrAtt**	0.738	0.760	0.532	0.381	0.274
**E3FP-based**	0.815	0.845	0.710	0.632	0.522
**2DFP-based**	0.822	0.859	0.725	0.646	0.537
**DeepCAT**	0.777	0.763	0.570	0.486	0.379
**DeepDTA**	0.797	0.795	0.638	0.541	0.439
**Random**	0.501	0.502	0.281	0.360	0.003
**RF**	0.661	0.583	0.347	0.308	0.096
**PhyChemDG**	0.648	0.699	0.434	0.440	0.214

Supplementary Table 9: Cold-start for targets: performance details in various folds

**Fold-0**	Accuracy	AUC ROC	AUC PR	F1 score	MCC
TransformerCPI	0.732	0.766	0.502	0.531	0.344
RF	0.535	0.429	0.241	0.239	-0.091
Random	0.505	0.501	0.277	0.358	0.005
PhyChemDG	0.649	0.645	0.362	0.347	0.107
PhyGrAtt	0.707	0.699	0.419	0.400	0.213
DeepCAT	0.720	0.728	0.455	0.536	0.340
DeepDTA	0.723	0.500	0.277	0.000	0.000
2DFP-based	0.771	0.773	0.598	0.521	0.383
E3FP-based	0.782	0.798	0.641	0.594	0.450
AlphaFoldGrAtts	0.728	0.704	0.439	0.153	0.125
BERT-based	0.738	0.736	0.483	0.385	0.253
UniRep-based	0.750	0.758	0.509	0.424	0.296
DeepConv-DTI	0.821	0.878	0.762	0.688	0.564
IIFDTI	0.788	0.816	0.644	0.581	0.443
**Fold-1**	Accuracy	AUC ROC	AUC PR	F1 score	MCC
TransformerCPI	0.729	0.787	0.606	0.613	0.405
RF	0.494	0.401	0.292	0.273	-0.115
PhyChemDG	0.628	0.675	0.447	0.511	0.218
Random	0.498	0.498	0.323	0.393	-0.003
PhyGrAtt	0.659	0.669	0.461	0.288	0.134
DeepCAT	0.734	0.784	0.571	0.532	0.359
DeepDTA	0.659	0.732	0.507	0.596	0.353
2DFP-based	0.759	0.805	0.638	0.567	0.419
E3FP-based	0.776	0.807	0.687	0.615	0.474
AlphaFoldGrAtts	0.662	0.722	0.538	0.040	0.066
UniRep-based	0.699	0.756	0.573	0.401	0.262
BERT-based	0.659	0.747	0.545	0.000	0.000
DeepConv-DTI	0.809	0.884	0.772	0.722	0.577
IIFDTI	0.743	0.808	0.631	0.603	0.415
**Fold-2**	Accuracy	AUC ROC	AUC PR	F1 score	MCC
TransformerCPI	0.720	0.739	0.489	0.460	0.281
RF	0.544	0.441	0.269	0.240	-0.086
PhyChemDG	0.647	0.693	0.409	0.494	0.239
Random	0.495	0.492	0.275	0.354	-0.010
PhyGrAtt	0.678	0.655	0.396	0.225	0.077
DeepCAT	0.703	0.711	0.424	0.439	0.239
DeepDTA	0.632	0.698	0.417	0.535	0.305
2DFP-based	0.738	0.766	0.495	0.541	0.358
E3FP-based	0.741	0.778	0.541	0.582	0.394
AlphaFoldGrAtts	0.706	0.668	0.423	0.161	0.118

BERT-based	0.726	0.740	0.518	0.349	0.241
UniRep-based	0.726	0.749	0.524	0.368	0.247
DeepConv-DTI	0.829	0.891	0.754	0.703	0.584
IIFDTI	0.742	0.779	0.563	0.532	0.357
**Fold-3**	Accuracy	AUC ROC	AUC PR	F1 score	MCC
TransformerCPI	0.708	0.761	0.528	0.567	0.347
RF	0.548	0.395	0.284	0.202	-0.104
PhyChemDG	0.580	0.650	0.414	0.535	0.226
Random	0.495	0.496	0.336	0.397	-0.013
PhyGrAtt	0.672	0.700	0.490	0.373	0.183
DeepCAT	0.660	0.510	0.353	0.000	0.000
DeepDTA	0.630	0.705	0.482	0.592	0.323
2DFP-based	0.759	0.810	0.676	0.573	0.431
E3FP-based	0.791	0.833	0.734	0.623	0.502
AlphaFoldGrAtts	0.677	0.747	0.541	0.053	0.081
UniRep-based	0.699	0.729	0.546	0.368	0.238
BERT-based	0.700	0.688	0.526	0.330	0.233
DeepConv-DTI	0.785	0.857	0.737	0.685	0.523
IIFDTI	0.735	0.787	0.603	0.599	0.401
**Fold-4**	Accuracy	AUC ROC	AUC PR	F1 score	MCC
TransformerCPI	0.763	0.802	0.607	0.583	0.419
RF	0.533	0.411	0.250	0.229	-0.106
PhyChemDG	0.686	0.708	0.455	0.405	0.198
Random	0.497	0.496	0.271	0.351	-0.005
PhyGrAtt	0.695	0.669	0.425	0.240	0.119
DeepCAT	0.780	0.791	0.577	0.521	0.396
DeepDTA	0.653	0.703	0.396	0.517	0.289
2DFP-based	0.787	0.811	0.592	0.599	0.454
E3FP-based	0.718	0.772	0.478	0.552	0.370
AlphaFoldGrAtts	0.710	0.706	0.454	0.146	0.123
BERT-based	0.736	0.769	0.549	0.384	0.276
UniRep-based	0.725	0.778	0.538	0.259	0.212
DeepConv-DTI	0.837	0.894	0.801	0.702	0.594
IIFDTI	0.770	0.814	0.625	0.579	0.424
**Fold-5**	Accuracy	AUC ROC	AUC PR	F1 score	MCC
TransformerCPI	0.780	0.812	0.540	0.550	0.405
RF	0.554	0.467	0.231	0.279	-0.026
PhyChemDG	0.541	0.662	0.379	0.435	0.175
Random	0.499	0.498	0.266	0.346	-0.003
PhyGrAtt	0.685	0.672	0.361	0.332	0.127
DeepCAT	0.785	0.806	0.613	0.534	0.407
DeepDTA	0.776	0.798	0.611	0.551	0.404
2DFP-based	0.804	0.829	0.659	0.575	0.462

E3FP-based	0.776	0.804	0.563	0.579	0.427
AlphaFoldGrAtts	0.757	0.723	0.422	0.222	0.172
UniRep-based	0.771	0.770	0.505	0.384	0.283
BERT-based	0.773	0.777	0.516	0.405	0.298
DeepConv-DTI	0.864	0.918	0.801	0.714	0.626
IIFDTI	0.830	0.876	0.676	0.657	0.543
**Fold-6**	Accuracy	AUC ROC	AUC PR	F1 score	MCC
TransformerCPI	0.736	0.791	0.609	0.531	0.356
RF	0.561	0.422	0.278	0.224	-0.077
PhyChemDG	0.573	0.658	0.415	0.506	0.196
Random	0.502	0.497	0.270	0.352	0.000
PhyGrAtt	0.688	0.721	0.467	0.232	0.141
DeepCAT	0.787	0.807	0.616	0.507	0.406
DeepDTA	0.694	0.736	0.471	0.493	0.279
2DFP-based	0.801	0.823	0.656	0.584	0.465
E3FP-based	0.809	0.835	0.695	0.598	0.503
AlphaFoldGrAtts	0.693	0.705	0.447	0.070	0.063
BERT-based	0.723	0.772	0.580	0.390	0.280
UniRep-based	0.684	0.753	0.504	0.000	0.000
DeepConv-DTI	0.820	0.891	0.788	0.722	0.589
IIFDTI	0.747	0.822	0.634	0.627	0.440
**Fold-7**	Accuracy	AUC ROC	AUC PR	F1 score	MCC
TransformerCPI	0.782	0.824	0.589	0.577	0.430
RF	0.524	0.494	0.255	0.321	-0.004
PhyChemDG	0.698	0.707	0.426	0.412	0.208
Random	0.495	0.494	0.221	0.305	-0.009
PhyGrAtt	0.726	0.706	0.432	0.381	0.213
DeepCAT	0.806	0.801	0.542	0.492	0.386
DeepDTA	0.773	0.785	0.496	0.520	0.372
2DFP-based	0.811	0.821	0.583	0.556	0.437
E3FP-based	0.807	0.823	0.638	0.615	0.486
AlphaFoldGrAtts	0.748	0.731	0.444	0.155	0.136
UniRep-based	0.763	0.770	0.508	0.418	0.294
BERT-based	0.767	0.766	0.517	0.419	0.302
DeepConv-DTI	0.860	0.910	0.787	0.729	0.634
IIFDTI	0.807	0.831	0.640	0.588	0.466
**Fold-8**	Accuracy	AUC ROC	AUC PR	F1 score	MCC
TransformerCPI	0.765	0.822	0.619	0.596	0.431
RF	0.532	0.435	0.250	0.251	-0.085
PhyChemDG	0.692	0.742	0.484	0.497	0.281
Random	0.500	0.502	0.261	0.345	0.003
PhyGrAtt	0.735	0.734	0.484	0.243	0.194
DeepCAT	0.800	0.828	0.623	0.507	0.420

DeepDTA	0.709	0.756	0.437	0.548	0.358
2DFP-based	0.808	0.839	0.640	0.550	0.452
E3FP-based	0.818	0.830	0.661	0.616	0.504
AlphaFoldGrAtts	0.731	0.745	0.492	0.134	0.144
BERT-based	0.755	0.764	0.544	0.415	0.305
UniRep-based	0.749	0.768	0.541	0.398	0.285
DeepConv-DTI	0.842	0.901	0.790	0.707	0.599
IIFDTI	0.805	0.855	0.671	0.639	0.507
**Fold-9**	Accuracy	AUC ROC	AUC PR	F1 score	MCC
TransformerCPI	0.777	0.822	0.609	0.561	0.412
RF	0.579	0.470	0.248	0.284	-0.008
PhyChemDG	0.692	0.732	0.455	0.476	0.267
Random	0.496	0.497	0.267	0.345	-0.008
PhyGrAtt	0.723	0.697	0.373	0.152	0.065
DeepCAT	0.785	0.822	0.611	0.524	0.404
DeepDTA	0.638	0.707	0.401	0.520	0.297
2DFP-based	0.811	0.820	0.636	0.596	0.485
E3FP-based	0.823	0.852	0.678	0.612	0.508
AlphaFoldGrAtts	0.748	0.722	0.452	0.109	0.137
UniRep-based	0.761	0.784	0.527	0.421	0.298
BERT-based	0.742	0.768	0.461	0.000	0.000
DeepConv-DTI	0.856	0.916	0.801	0.711	0.616
IIFDTI	0.801	0.851	0.657	0.609	0.476

Supplementary Table 10: Average performance of the models using cold-start splitting scenario for targets.

**MODELS**	**Accuracy**	**AUC ROC**	**AUC PR**	**F1 score**	**MCC**
**UniRep-based**	0.733	0.762	0.527	0.344	0.241
**DeepConv-DTI**	0.832	0.894	0.779	0.708	0.591
**IIFDTI**	0.777	0.824	0.634	0.601	0.447
**TransformerCPI**	0.749	0.792	0.570	0.557	0.383
**BERT-based**	0.732	0.753	0.524	0.308	0.219
**AlphaFoldGrAtts**	0.716	0.717	0.465	0.124	0.116
**PhyGrAtt**	0.697	0.692	0.431	0.286	0.147
**E3FP-based**	0.784	0.813	0.632	0.599	0.462
**2DFP-based**	0.785	0.810	0.617	0.566	0.435
**DeepCAT**	0.756	0.759	0.539	0.459	0.336
**DeepDTA**	0.689	0.712	0.450	0.487	0.298
**Random**	0.498	0.497	0.277	0.355	-0.004
**RF**	0.540	0.436	0.260	0.254	-0.070
**PhyChemDG**	0.638	0.687	0.425	0.462	0.211

Supplementary Table 11: Cold-start for compounds: performance details in various folds

**Fold-0**	Accuracy	AUC ROC	AUC PR	F1 score	MCC
TransformerCPI	0.841	0.904	0.784	0.710	0.604
RF	0.683	0.595	0.310	0.276	0.080
Random	0.496	0.493	0.255	0.336	-0.009
PhyChemDG	0.634	0.689	0.358	0.467	0.236
PhyGrAtt	0.765	0.772	0.507	0.377	0.276
E3FP-based	0.663	0.717	0.391	0.502	0.294
2DFP-based	0.754	0.774	0.485	0.533	0.366
DeepCAT	0.718	0.741	0.462	0.496	0.304
DeepDTA	0.741	0.500	0.259	0.000	0.000
AlphaFoldGrAtts	0.763	0.765	0.492	0.277	0.227
UniRep-based	0.775	0.788	0.532	0.440	0.328
BERT-based	0.772	0.776	0.523	0.434	0.317
DeepConv-DTI	0.891	0.943	0.865	0.782	0.709
IIFDTI	0.859	0.912	0.791	0.722	0.628
**Fold-1**	Accuracy	AUC ROC	AUC PR	F1 score	MCC
TransformerCPI	0.820	0.896	0.754	0.705	0.588
RF	0.671	0.602	0.335	0.316	0.104
PhyChemDG	0.580	0.697	0.382	0.519	0.300
Random	0.499	0.497	0.259	0.340	-0.004
PhyGrAtt	0.742	0.729	0.462	0.178	0.164
E3FP-based	0.745	0.500	0.255	0.000	0.000
2DFP-based	0.741	0.785	0.542	0.558	0.383
DeepCAT	0.740	0.500	0.261	0.000	0.000
DeepDTA	0.740	0.500	0.260	0.000	0.000
AlphaFoldGrAtts	0.749	0.748	0.492	0.251	0.211
BERT-based	0.756	0.771	0.525	0.410	0.292
UniRep-based	0.758	0.772	0.532	0.397	0.290
DeepConv-DTI	0.874	0.933	0.853	0.767	0.680
IIFDTI	0.845	0.902	0.790	0.710	0.604
**Fold-2**	Accuracy	AUC ROC	AUC PR	F1 score	MCC
TransformerCPI	0.808	0.896	0.762	0.694	0.576
RF	0.683	0.599	0.327	0.288	0.095
PhyChemDG	0.717	0.732	0.431	0.360	0.191
Random	0.499	0.497	0.260	0.343	-0.003
PhyGrAtt	0.755	0.767	0.501	0.425	0.291
E3FP-based	0.684	0.726	0.436	0.500	0.292
2DFP-based	0.723	0.779	0.514	0.556	0.371
DeepCAT	0.737	0.500	0.263	0.000	0.000
DeepDTA	0.737	0.500	0.263	0.000	0.000

AlphaFoldGrAtts	0.754	0.770	0.497	0.301	0.231
UniRep-based	0.771	0.788	0.552	0.454	0.338
BERT-based	0.763	0.766	0.518	0.401	0.295
DeepConv-DTI	0.895	0.939	0.876	0.795	0.725
IIFDTI	0.857	0.914	0.815	0.730	0.633
**Fold-3**	Accuracy	AUC ROC	AUC PR	F1 score	MCC
TransformerCPI	0.819	0.901	0.782	0.710	0.593
RF	0.675	0.593	0.332	0.322	0.115
PhyChemDG	0.589	0.668	0.388	0.449	0.174
Random	0.495	0.500	0.253	0.333	-0.005
PhyGrAtt	0.754	0.779	0.529	0.433	0.304
E3FP-based	0.703	0.746	0.512	0.538	0.325
2DFP-based	0.731	0.782	0.480	0.549	0.374
DeepCAT	0.748	0.500	0.252	0.000	0.000
DeepDTA	0.746	0.793	0.541	0.559	0.391
AlphaFoldGrAtts	0.747	0.759	0.504	0.261	0.219
BERT-based	0.759	0.786	0.539	0.436	0.315
UniRep-based	0.757	0.764	0.530	0.407	0.298
DeepConv-DTI	0.885	0.940	0.874	0.786	0.707
IIFDTI	0.854	0.912	0.814	0.721	0.624
**Fold-4**	Accuracy	AUC ROC	AUC PR	F1 score	MCC
TransformerCPI	0.814	0.893	0.752	0.705	0.581
RF	0.673	0.587	0.337	0.303	0.102
PhyChemDG	0.585	0.683	0.395	0.521	0.281
Random	0.500	0.503	0.274	0.356	0.003
PhyGrAtt	0.736	0.743	0.494	0.217	0.191
E3FP-based	0.666	0.700	0.470	0.471	0.231
2DFP-based	0.758	0.806	0.582	0.576	0.408
DeepCAT	0.726	0.737	0.491	0.454	0.275
DeepDTA	0.726	0.676	0.414	0.000	0.000
AlphaFoldGrAtts	0.749	0.775	0.538	0.343	0.267
UniRep-based	0.761	0.803	0.572	0.471	0.342
BERT-based	0.751	0.776	0.540	0.413	0.297
DeepConv-DTI	0.885	0.935	0.875	0.798	0.718
IIFDTI	0.852	0.916	0.815	0.738	0.634
**Fold-5**	Accuracy	AUC ROC	AUC PR	F1 score	MCC
TransformerCPI	0.804	0.886	0.779	0.712	0.576
RF	0.654	0.583	0.357	0.314	0.098
PhyChemDG	0.686	0.723	0.472	0.418	0.209
Random	0.501	0.503	0.290	0.367	0.003
PhyGrAtt	0.732	0.773	0.555	0.402	0.282
E3FP-based	0.669	0.702	0.478	0.531	0.286
2DFP-based	0.763	0.807	0.612	0.585	0.419

DeepCAT	0.676	0.717	0.484	0.475	0.243
DeepDTA	0.738	0.772	0.562	0.540	0.357
AlphaFoldGrAtts	0.722	0.753	0.527	0.288	0.219
BERT-based	0.733	0.791	0.557	0.397	0.282
UniRep-based	0.738	0.779	0.582	0.407	0.299
DeepConv-DTI	0.884	0.941	0.884	0.809	0.726
IIFDTI	0.853	0.916	0.817	0.757	0.652
**Fold-6**	Accuracy	AUC ROC	AUC PR	F1 score	MCC
TransformerCPI	0.776	0.874	0.748	0.687	0.540
RF	0.651	0.593	0.364	0.348	0.116
PhyChemDG	0.649	0.688	0.465	0.401	0.153
Random	0.503	0.498	0.291	0.369	0.003
PhyGrAtt	0.713	0.709	0.476	0.134	0.148
E3FP-based	0.749	0.767	0.525	0.538	0.368
2DFP-based	0.758	0.797	0.587	0.571	0.404
DeepCAT	0.642	0.669	0.406	0.540	0.300
DeepDTA	0.723	0.768	0.545	0.534	0.337
AlphaFoldGrAtts	0.729	0.751	0.531	0.323	0.242
UniRep-based	0.702	0.755	0.500	0.000	0.000
BERT-based	0.730	0.761	0.545	0.370	0.262
DeepConv-DTI	0.876	0.935	0.883	0.792	0.704
IIFDTI	0.827	0.886	0.792	0.703	0.582
**Fold-7**	Accuracy	AUC ROC	AUC PR	F1 score	MCC
TransformerCPI	0.805	0.894	0.803	0.732	0.595
RF	0.632	0.564	0.368	0.316	0.078
PhyChemDG	0.653	0.679	0.450	0.388	0.153
Random	0.498	0.492	0.295	0.370	-0.007
PhyGrAtt	0.720	0.755	0.560	0.426	0.285
E3FP-based	0.605	0.671	0.410	0.534	0.268
2DFP-based	0.749	0.776	0.596	0.571	0.394
DeepCAT	0.700	0.725	0.490	0.454	0.252
DeepDTA	0.717	0.749	0.501	0.533	0.330
AlphaFoldGrAtts	0.705	0.725	0.521	0.281	0.204
BERT-based	0.719	0.753	0.563	0.382	0.270
UniRep-based	0.720	0.747	0.569	0.373	0.272
DeepConv-DTI	0.875	0.932	0.886	0.805	0.714
IIFDTI	0.841	0.905	0.834	0.754	0.637
**Fold-8**	Accuracy	AUC ROC	AUC PR	F1 score	MCC
TransformerCPI	0.789	0.873	0.750	0.715	0.562
RF	0.629	0.559	0.366	0.317	0.078
PhyChemDG	0.608	0.646	0.409	0.489	0.189
Random	0.493	0.490	0.302	0.374	-0.014
PhyGrAtt	0.709	0.742	0.549	0.479	0.292

E3FP-based	0.635	0.684	0.459	0.508	0.242
2DFP-based	0.761	0.794	0.603	0.614	0.441
DeepCAT	0.712	0.736	0.557	0.515	0.311
DeepDTA	0.706	0.731	0.543	0.506	0.297
AlphaFoldGrAtts	0.704	0.726	0.544	0.282	0.220
UniRep-based	0.710	0.748	0.568	0.348	0.253
BERT-based	0.714	0.742	0.569	0.363	0.265
DeepConv-DTI	0.874	0.938	0.890	0.810	0.716
IIFDTI	0.836	0.906	0.831	0.756	0.633
**Fold-9**	Accuracy	AUC ROC	AUC PR	F1 score	MCC
TransformerCPI	0.779	0.879	0.781	0.703	0.548
RF	0.624	0.558	0.357	0.336	0.080
PhyChemDG	0.504	0.635	0.418	0.510	0.178
Random	0.501	0.499	0.296	0.371	0.002
PhyGrAtt	0.722	0.747	0.559	0.453	0.297
E3FP-based	0.647	0.691	0.503	0.519	0.252
2DFP-based	0.756	0.797	0.566	0.607	0.432
DeepCAT	0.706	0.500	0.294	0.000	0.000
DeepDTA	0.707	0.767	0.507	0.593	0.392
AlphaFoldGrAtts	0.707	0.725	0.526	0.278	0.208
BERT-based	0.684	0.732	0.496	0.000	0.000
UniRep-based	0.726	0.758	0.570	0.426	0.296
DeepConv-DTI	0.864	0.927	0.871	0.788	0.688
IIFDTI	0.820	0.894	0.802	0.732	0.600

Supplementary Table 12: Average performance of the models using cold-start splitting scenario for compounds.

MODELS	Accuracy	AUC ROC	AUC PR	F1 score	MCC
**UniRep-based**	0.742	0.770	0.551	0.372	0.271
**DeepConv-DTI**	0.880	0.936	0.876	0.793	0.709
**IIFDTI**	0.844	0.906	0.810	0.732	0.623
**TransformerCPI**	0.805	0.890	0.769	0.707	0.576
**BERT-based**	0.738	0.765	0.537	0.361	0.260
**AlphaFoldGrAtts**	0.733	0.750	0.517	0.288	0.225
**PhyGrAtt**	0.735	0.752	0.519	0.352	0.253
**E3FP-based**	0.677	0.690	0.444	0.464	0.256
**2DFP-based**	0.749	0.790	0.557	0.572	0.399
**DeepCAT**	0.710	0.632	0.396	0.293	0.168
**DeepDTA**	0.728	0.676	0.440	0.326	0.210
**Random**	0.499	0.497	0.277	0.356	-0.003
**RF**	0.657	0.583	0.345	0.314	0.094
**PhyChemDG**	0.621	0.684	0.417	0.452	0.206

Supplementary Table 13: Datasets

**Davis**	Positive Samples	Negative Samples	Neg/Pos	Size
Test Set	847	2,759	3.25	3,606
Training Set	5975	19,285	3.22	25,260
Dataset	6,822	22,044	3.23	28,866
**DrugBank**	Positive Samples	Negative Samples	Neg/Pos	Size
Test Set	2,168	10,778	4.97	12,946
Training Set	15,039	75,895	5.04	90,934
Dataset	17,207	86,673	5.03	103,880
**KiBA**	Positive Samples	Negative Samples	Neg/Pos	Size
Test Set	9,718	19,430	1.99	29,148
Training Set	66,898	137,865	2.06	204,763
Dataset	76,616	157,295	2.05	233,911
**WT-Mut**	Positive Samples	Negative Samples	Neg/Pos	Size
Test Set	1,995	3,824	1.91	5,819
Training Set	95,518	163,599	1.71	259,117
Dataset	97,513	167,423	1.71	264,936

Supplementary Table 14: Large-sized Dataset: KiBA

**KiBA: Without Limitation**					
Model	Accuracy	AUC ROC	AUC PR	F1 score	MCC
TransformerCPI	0.724	0.797	0.655	0.635	0.426
PhyChemDG	0.604	0.543	0.346	0.231	-0.005
PhyGrAtt	0.706	0.735	0.558	0.431	0.275
RF	0.572	0.596	0.406	0.474	0.139
BERT-based	0.682	0.677	0.495	0.242	0.166
DeepDTA	0.667	0.501	0.332	0.000	0.000
DeepCAT	0.667	0.703	0.499	0.000	-0.004
2DFP-based	0.668	0.584	0.399	0.046	0.053
E3FP-based	0.666	0.692	0.477	0.000	-0.003
AlphaFoldGrAtts	0.695	0.710	0.533	0.291	0.216
UniRep-based	0.667	0.682	0.479	0.000	-0.006
DeepConv-DTI	0.772	0.853	0.760	0.690	0.518
IIFDTI	0.743	0.795	0.662	0.621	0.426
**KiBA: Limited Rotatable Bonds**					
Model	Accuracy	AUC ROC	AUC PR	F1 score	MCC
TransformerCPI	0.738	0.802	0.662	0.637	0.439
PhyGrAtt	0.659	0.604	0.385	0.154	0.051
BERT-based	0.684	0.659	0.473	0.338	0.188
2DFP-based	0.671	0.597	0.410	0.149	0.084
E3FP-based	0.683	0.636	0.477	0.138	0.134
AlphaFoldGrAtts	0.703	0.704	0.519	0.373	0.243
DeepConv-DTI	0.788	0.862	0.772	0.700	0.542
IIFDTI	0.769	0.825	0.709	0.642	0.471
**KiBA: Ratio LRB**					
Model	Accuracy	AUC ROC	AUC PR	F1 score	MCC
TransformerCPI	0.726	0.803	0.670	0.638	0.432
PhyGrAtt	0.677	0.661	0.483	0.214	0.141
E3FP-based	0.668	0.703	0.492	0.000	0.000
2DFP-based	0.659	0.597	0.418	0.272	0.114
AlphaFoldGrAtts	0.702	0.710	0.532	0.436	0.267
BERT-based	0.679	0.667	0.482	0.297	0.171
DeepConv-DTI	0.785	0.852	0.763	0.686	0.523
IIFDTI	0.743	0.803	0.669	0.608	0.417

Supplementary Table 15: Medium-sized Dataset: DrugBank

**DrugBank: Without Limitation**					
Model	Accuracy	AUC ROC	AUC PR	F1 score	MCC
TransformerCPI	0.950	0.950	0.904	0.846	0.817
PhyChemDG	0.400	0.418	0.135	0.208	-0.108
PhyGrAtt	0.827	0.768	0.379	0.341	0.262
RF	0.760	0.452	0.146	0.062	-0.066
BERT-based	0.868	0.867	0.594	0.547	0.480
DeepDTA	0.833	0.494	0.165	0.000	0.000
DeepCAT	0.833	0.498	0.167	0.000	0.000
2DFP-based	0.833	0.784	0.331	0.000	0.000
E3FP-based	0.833	0.653	0.221	0.000	0.000
AlphaFoldGrAtts	0.813	0.771	0.357	0.344	0.244
UniRep-based	0.866	0.878	0.610	0.558	0.483
IIFDTI	0.967	0.984	0.958	0.901	0.881
DeepConv-DTI	0.956	0.969	0.927	0.868	0.842
**DrugBank: Limited Rotatable Bonds**					
Model	Accuracy	AUC ROC	AUC PR	F1 score	MCC
TransformerCPI	0.951	0.955	0.907	0.849	0.821
PhyGrAtt	0.844	0.819	0.452	0.254	0.247
BERT-based	0.837	0.879	0.565	0.000	0.000
AlphaFoldGrAtts	0.839	0.824	0.479	0.088	0.133
2DFP-based	0.871	0.803	0.562	0.435	0.428
E3FP-based	0.836	0.477	0.156	0.001	0.000
DeepConv-DTI	0.925	0.965	0.920	0.798	0.761
IIFDTI	0.969	0.979	0.946	0.901	0.883
**DrugBank: Ratio LRB**					
Model	Accuracy	AUC ROC	AUC PR	F1 score	MCC
TransformerCPI	0.952	0.960	0.904	0.841	0.813
PhyGrAtt	0.835	0.798	0.420	0.446	0.350
BERT-based	0.849	0.872	0.560	0.120	0.194
AlphaFoldGrAtts	0.850	0.826	0.439	0.255	0.253
E3FP-based	0.842	0.525	0.173	0.000	0.000
2DFP-based	0.842	0.883	0.557	0.000	0.000
DeepConv-DTI	0.957	0.967	0.927	0.865	0.840
IIFDTI	0.963	0.977	0.943	0.879	0.859

Supplementary Table 16: Small-sized Dataset: Davis

Model	Accuracy	AUC ROC	AUC PR	F1 score	MCC
TransformerCPI	0.704	0.674	0.370	0.416	0.221
PhyChemDG	0.765	0.689	0.414	0.000	0.000
PhyGrAtt	0.626	0.591	0.400	0.406	0.169
RF	0.638	0.489	0.229	0.224	-0.012
BERT-based	0.765	0.557	0.256	0.000	0.000
DeepDTA	0.765	0.479	0.223	0.000	0.000
DeepCAT	0.762	0.588	0.272	0.009	-0.002
2DFP-based	0.765	0.486	0.229	0.000	0.000
E3FP-based	0.765	0.500	0.235	0.000	0.000
AlphaFoldGrAtts	0.353	0.577	0.333	0.395	0.098
UniRep-based	0.765	0.538	0.254	0.000	0.000
IIFDTI	0.736	0.732	0.495	0.476	0.302
DeepConv-DTI	0.721	0.784	0.599	0.545	0.381

Supplementary Table 17: Training on Compound-Wildtype Target interactions and test on Compound-Mutated Target interactions

Model	Accuracy	AUC ROC	AUC PR	F1 score	MCC
TransformerCPI	0.795	0.833	0.790	0.691	0.538
PhyChemDG	0.661	0.649	0.506	0.330	0.157
PhyGrAtt	0.686	0.766	0.670	0.621	0.382
RF	0.619	0.571	0.418	0.188	0.004
2DFP-based	0.683	0.666	0.520	0.258	0.198
AlphaFoldGrAtts	0.651	0.670	0.434	0.502	0.257
BERT-based	0.728	0.703	0.638	0.463	0.348
DeepCAT	0.656	0.683	0.469	0.586	0.321
DeepDTA	0.657	0.509	0.344	0.000	0.000
E3FP-based	0.596	0.629	0.400	0.547	0.236
UniRep-based	0.669	0.634	0.514	0.456	0.228
DeepConv-DTI	0.816	0.831	0.800	0.654	0.587
IIFDTI	0.776	0.709	0.688	0.567	0.481
